# Quality of family relationships and outcomes of dementia: a systematic review

**DOI:** 10.1136/bmjopen-2016-015538

**Published:** 2018-01-21

**Authors:** Hannah B Edwards, Sharea Ijaz, Penny F Whiting, Verity Leach, Alison Richards, Sarah J Cullum, Richard IL Cheston, Jelena Savović

**Affiliations:** 1 Bristol Medical School, University of Bristol, Bristol, UK; 2 National Institute for Health Research (NIHR) Collaboration for Leadership in Applied Health Research and Care (CLAHRC) West, University Hospitals Bristol NHS Foundation Trust, Bristol, UK; 3 Psychological Medicine, School of Medicine, Faculty of Medical and Health Sciences, University of Auckland, Auckland, New Zealand; 4 Department of Health and Social Sciences, University of the West of England, Bristol, UK

**Keywords:** dementia, carers, family relationships, institutionalisation, challenging behaviour

## Abstract

**Objectives:**

To evaluate the association between the quality of relationship between a person with dementia and their family carer and outcomes for the person with dementia.

**Design:**

Systematic review.

**Eligibility criteria:**

Cohort studies of people with clinically diagnosed dementia and their main carers. Exposures of interest were any elements of relationship quality, for example, attachment style, expressed emotion and coping style. Our primary outcome was institutionalisation, and secondary outcomes were hospitalisation, death, quality of life and behavioural and psychiatric symptoms of dementia (‘challenging behaviour’).

**Data sources:**

MEDLINE, Embase, Web of Science, PsycInfo, the Cochrane Library and Opengrey were searched from inception to May 2017.

**Study appraisal and synthesis methods:**

The Newcastle-Ottawa Scale was used to assess risk of bias. A narrative synthesis of results was performed due to differences between studies.

**Results:**

Twenty studies were included. None of the studies controlled for all prespecified confounding factors (age, gender, socioeconomic status and severity of dementia). Reporting of results was inadequate with many studies simply reporting whether associations were ‘statistically significant’ without providing effect size estimates or CIs. There was a suggestion of an association between relationship factors and global challenging behaviour. All studies evaluating global challenging behaviour provided statistical evidence of an association (most P values below 0.02). There was no consistent evidence for an association for any other outcome assessed.

**Conclusions:**

There is currently no strong or consistent evidence on the effects of relationship factors on institutionalisation, hospitalisation, death or quality of life for people with dementia. There was a suggestion of an association between relationship factors and challenging behaviour, although the evidence for this was weak. To improve our ability to support those with dementia and their families, further robust studies are needed.

**PROSPERO registration number:**

CRD42015020518.

**Strengths and limitations of this study**

- Broad search strategy so unlikely to have missed relevant studies.
- Double screening minimises selection bias.
- We were not able to assess publication bias and the potential for selective reporting of outcomes within studies.

## Background

Dementia is a key public health concern in the UK^1–3^ with around 7% of all those over 65 affected, and the numbers of people with dementia predicted to double every 20 years.^4–6^ Institutionalisation (being placed in a full-time care/nursing home) is a key outcome for people with dementia, their families and the healthcare system. Although in many circumstances institutionalisation may be the best or only option for the person affected by dementia, most people report that they would prefer to stay living in their own home.^7^ Recent media attention to a few very poorly run care homes has also led to concerns about institutionalisation.^8–10^ Additionally, the financial cost of full-time care is very high, both for affected individuals and their families, and for the public, as public taxes are used to contribute to care home fees. Consequently, for some time it has been UK government policy to help families to continue supporting people with dementia at home, specifically to delay or avoid institutionalisation.^11^

The quality of relationship between the person with dementia and people who care for them has been linked with a range of outcomes including institutionalisation, cognitive and functional decline and quality of life (QoL).^12–21^ There is also growing interest in the potential for psychosocial interventions to improve outcomes by enhancing interactions within families.^22 23^ If families are better equipped to cope with the psychological and emotional challenges of dementia, then care at home may be sustained for longer. However, it is not clear which elements of the relationship are predictive of early institutionalisation or which lead to a faster decline. This evidence is necessary both to justify and to help to develop early psychosocial interventions, ideally at the point of diagnosis.

To address this issue, we performed a systematic review of the evidence on how elements of relationship quality between the person with dementia and their main informal (family) carer are associated with outcomes for the person with dementia.

## Methods

This study was a systematic review, registered with the PROSPERO International Prospective Register of Systematic Reviews, registration number CRD42015020518. The full protocol has been published.^24^

### Eligibility criteria

Only cohort studies (prospective and retrospective) were included in the review. Relevant systematic reviews were obtained and used as a means of identifying other original studies.^24^ Qualitative, case–control (unless nested in a prospective cohort), and cross-sectional studies were excluded.

The population of interest was people with dementia and their main informal caregiver (most commonly a spouse or child). Professional paid caregivers were excluded. People with all types of clinically diagnosed dementia were included.

The exposures of interest were factors that capture an element of relationship quality. We adopted a broad definition of ‘relationship quality’ as how happy or satisfied an individual is in their relationship.^25^ Attachment style, coping style, affection and expressed emotion (EE) were all identified at the design stage as key exposures of interest. While affection is a relatively straightforward term, attachment style, coping and EE relate to specific psychological constructs. ‘Attachment style’ is a term originally developed to understand the emotional relationship between children and parents but has since been extended to adult romantic relationships. Four main styles of attachment have been identified in adults: secure, anxious–preoccupied, dismissive–avoidant and fearful–avoidant.^26 27^ Coping is a wide-ranging construct that includes elements that are clearly measures of relationship quality (eg, ‘relationship-focused coping’) and those that are more individual in nature (eg, acceptance coping). However, as even individual coping styles are typically initiated in response to aspects of relationships, we felt that this was an appropriate exposure to capture. ‘EE’ is a measure of the family environment based on how the relatives of a psychiatric patient spontaneously talk about/to the patient. High levels of EE have been associated with worse prognosis in a number of mental illnesses including schizophrenia. This may be due to emotional overinvolvement, which can be experienced as hostility, criticism and intolerance.^28 29^

Two other notable factors emerged as key exposures when reviewing the literature: ‘mutuality’ and ‘boundary ambiguity’. Mutuality is a cluster concept capturing levels of positive engaging interaction, attachment and emotional support. Boundary ambiguity involves uncertainty about whether a person is in or out of the family group. This occurs as a result of significant changes in that person including those cognitive, functional, mood and personality changes that are indicative of dementia. Boundary ambiguity is associated with and taken as an indicator of emotional distancing and the withdrawal of the caregiver from the person with dementia. Other factors emerging from the literature were included if they captured an element of relationship quality, and this was assessed on a case-by-case basis through discussion with the study team. Carer abuse was excluded, as this was considered to be a different area of research. Overall measures of carer burden were also excluded as an exposure because they relate more to cognitive and functional levels in dementia than to relationship quality.

The primary outcome of interest was institutionalisation. This is a key event in the course of dementia, both socially and financially, from the perspectives of the individual, their family and the public. Secondary outcomes were hospitalisations, death (or time to death), QoL and challenging behaviour (also referred to as the behavioural and psychological symptoms of dementia or BPSD). Examples of challenging behaviour can include depression, anxiety, aggression, paranoia, hallucinations and delusions. Studies measuring QoL or challenging behaviour as the outcome had to use validated assessment tools to be included in the review.

### Search strategy

MEDLINE, Embase, Web of Science, PsycInfo, the Cochrane Library and Opengrey were searched from inception to May 2017 without any language restrictions. The full search strategy is available in the supplementary data of our published protocol.^24^ All results were imported into an Endnote X7 reference library and into a bespoke-built Microsoft Access 2013 database to manage screening.

### Selection of papers

The titles and abstracts of all identified papers were screened in duplicate by two reviewers working independently, and all potentially relevant papers were retrieved. All retrieved papers were read in full and assessed for eligibility using a standardised and piloted inclusion checklist, applied by two reviewers independently. Any discrepancies between the reviewers (at either stage of screening) were resolved through discussion.

### Data extraction

Data were extracted from included studies using a bespoke data collection form, which was piloted on six studies and amended as a result of the piloting. Data extracted included study characteristics, characteristics of the population of people with dementia and their carers, recruitment and response, the exposures and outcomes and how and when they were measured, and details of the analyses and results. The terminology for risk factors and outcomes was used as reported by the original study authors. Where numerical results were incompletely reported, where possible, relevant results (effect estimates, SE and 95% CIs) were calculated from the raw data. For continuous exposures, effect sizes were presented as change in the outcome for a one unit increase in the exposure. Multiple publications of the same dataset were counted as a single study. In the case where analyses of the same association was repeated in more than one report, our policy was either to include the result based on the largest sample size only, or if sample sizes were equivalent, then we would include the result from the most recent publication only. Data were extracted from the published reports only; we did not contact authors for additional unpublished information.

### Analysis

We planned to use meta-analysis to estimate summary effect sizes if there had been sufficient studies with similar populations, exposures and outcomes. As meta-analysis was not possible, a narrative synthesis of results is provided.^24^

### Assessment of methodological quality and risk of bias

The Newcastle-Ottawa Scale^30^ (NOS) was used to assess risk of bias for included reports. This is an eight-item questionnaire that assesses the following methodological criteria: representativeness of the exposed cohort; selection of the non-exposed cohort; ascertainment of exposure; demonstration that the outcome of interest was not present at the start of the study; comparability of cohorts (risk of confounding); assessment of exposure and outcome; and length and adequacy of follow-up. NOS allocates ‘stars’ for adequate methods but does not specifically advise calculating the sum of allocated stars to give an overall score.^30^ Empirical evidence also suggests that numerical quality scores are not helpful in differentiating between studies of high and low risk of bias.^31^ For this reason, we considered each of the eight criteria of the NOS tool separately and assessed the study as having adequate methods for that particular aspect of study conduct if the ‘star’ could be allocated for that NOS criterion. In our protocol, we considered 10 factors as potentially important confounding domains.^24^ During the piloting of the data extraction and risk of bias assessment, the team agreed a minimum number of essential confounders that all studies should have adjusted for. A study had to control for the following four prespecified factors to be at low risk of confounding: age, gender, socioeconomic status (SES) and dementia severity. We also recorded other confounders studies had adjusted for, in addition to the four main confounders used for risk of bias assessment.

## Results

**Figure 1:**
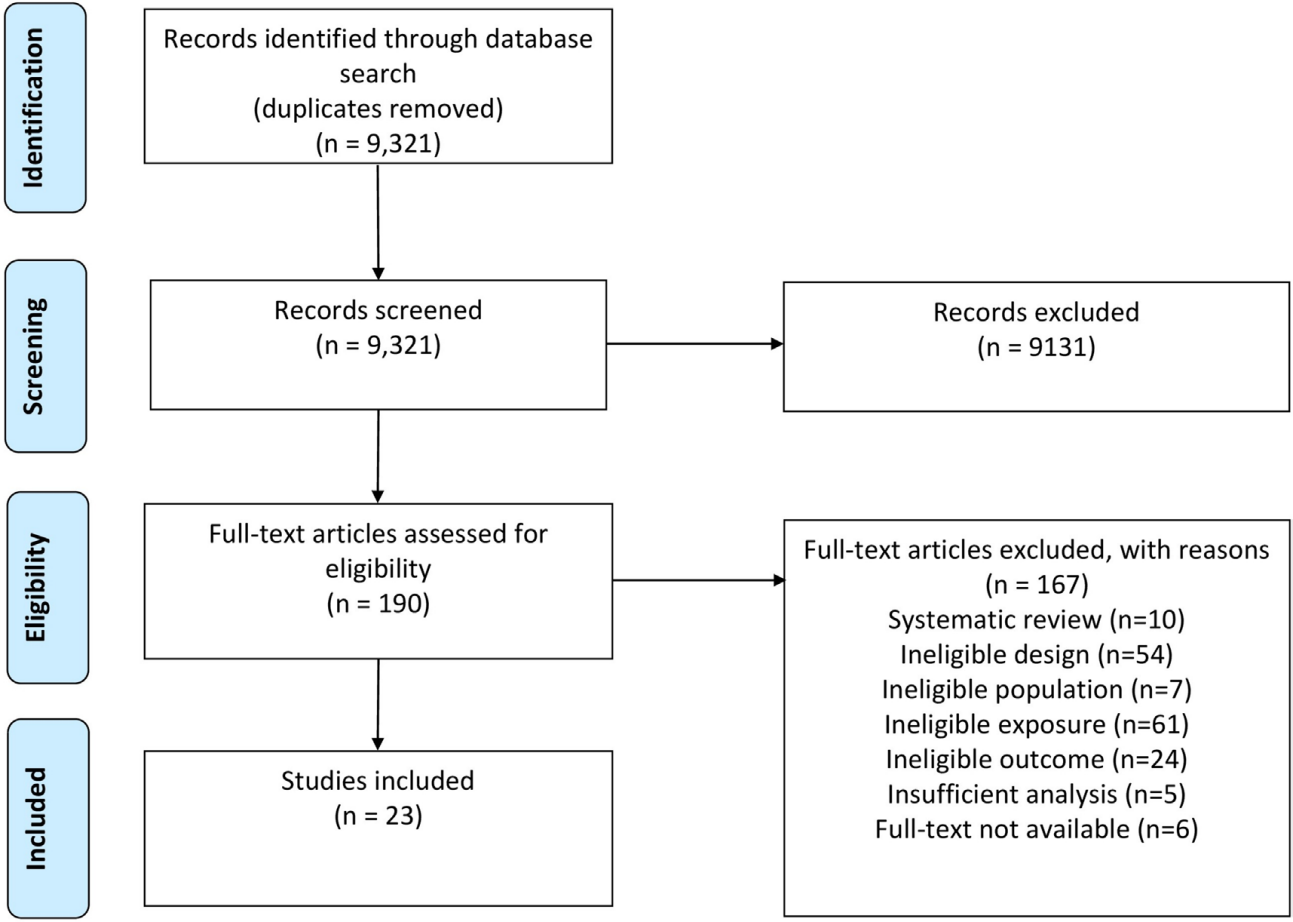
PRISMA flow diagram. PRISMA, Preferred Reporting Items for Systematic Review and Meta-Analysis.

The search identified 9321 potentially relevant papers. Of these, 190 papers were retrieved for full-text screening, 23 publications^20 32–53^ met the eligibility criteria (figure 1). Four of the 23 publications were based on data from one cohort study^32–35^ and so the total number of unique studies was 20 (2340 participants with dementia). As the four connected publications each contributed unique results, all were included in the review.

### Study characteristics

The majority of included unique studies (14 out of 20) came from the USA, and there was one each from the UK, Canada, Switzerland, Belgium, the Netherlands and Australia. Sample sizes in the relevant analyses ranged from 29 to 220 (mean 143). Years of publication ranged from 1990 to 2016, with most from the 1990s and early 2000s. The most frequently reported dementia type was Alzheimer’s disease, although in eight studies, the distribution of dementia types was not reported. Time since diagnosis ranged from 3 months to 6.5 years, although in six studies, this was not reported. The majority (13/20) did not report participants’ ethnicity, where this was reported, cohorts were predominantly Caucasian. The majority of caregivers included were spouses (100% in seven studies and more than 50% in another seven studies). Other carers were children of the person with dementia. The characteristics of included studies are presented in table 1.

**Table 1:** Characteristics of included studies

Study ID	Country	People with dementia						Carers					Exposures*	Outcomes*
		n	% Female	Dementia type	Dementia duration years (SD); range	Dementia severity Mean (SD); range	Age years (SD); range	n	% Female	% Spouse	% Child	Age years (SD); range		
Caron^36^	USA	60	22	AD	3.4 (2.5); 1–13	NR	68.26 (8.44); 53–90	60	79	86	8	63.57 (8.94); 30–84	Boundary ambiguity (caregiver closeout)	BPSD – depression; anxiety; paranoia; activity disturbance
Clare *et al*^37^	UK	51	50.98	61% AD, 21% VD, 17% mixed	NR	MMSE=24.5 (2.80); 18–30	76.75 (7.88); 55–91	51	70	57	31	65.06 (14.50); 33–89	Positive quality of relationship: Positive Affect Index	QoL Alzheimer’s Disease Scale
Kunik *et al* (four papers)^32–35^	USA	215	4.6	NR	≤1	NR	76 (6.2)	215	NR	NR	NR	NR	Mutuality, relationship strain	BPSD – aggression, depression, psychosis, hospitalisation
Spruytte *et al*^38^	Belgium	144	68.8	NR	≥0.25	GDS mean=6	82; 61–94	144	69.4	38.9	50.7	63; 38–90	Quality of relationship; criticism	Institutionalisation
Fisher and Lieberman^39^	USA	164	0.44	NR	6.5	MMSE=17 (6.25)	77 (7)	164	54% non-NHP, 47% NHP	0	100	45 (9)	Emotional closeness; boundary ambiguity; negative family feelings	Institutionalisation
Burgener and Twigg^20^	USA	96	57	NR	≤1	MMSE=21.52; 10–29	77.3 (7.8); 55–96	96	74	56	40	63.7 (12.2); 35–83	Quality of relationship	Institutionalisation
Stevens *et al*^40^	USA	215	71.2	NR	NR	MMSE=12.2 (8.7)	74.4 (8.2)	215	NR	47.9	40.5	NR	Coping strategies	Institutionalisation
Perren^41^	Switzerland	68	NR	60% AD, 25% VD, 15% other	NR	MMSE=21.6 (5.1)	74.8 (7.6)	68	NR	100	0	70.9 (10.0)	Attachment style	Overall care recipients problem behaviour
Wright^42^	USA	29		AD	4.8; 1–11	MMSE=18; 4–22, GDS=4.4; 2–6	67.5; 51–83	30	80	100	0	67.4; 51–81	Cohesion; tension; affection; present/past marital happiness	Institutionalisation
Wright *et al*^43^	USA	14	50	AD	2.8	MMSE of 21.29 (3.69); 16–26	65; 49–85	14	50	100	0	NR	Cohesion; tension; affection	BPSD – depression
Vitaliano *et al*^44^	USA	77	32	AD	4.3 (2.1)	NR	70.9 (6.9)	77	NR	100	0	67.2 (7.4)	Expressed emotion	Depression; negative behaviour
Bannister *et al*^45^	UK	116	72.4	72% probable AD, 17% VD, 10% LB, 3% unclear	NR	CAMCOG mean=45.2	79.8	116	59.5	14.7	NR	65	Coping strategies	Institutionalisation
de Vugt *et al*^46^	The Netherlands	99	56.6	74% AD, 19% VD, 2% frontal lobe, 3% Parkinson’s, 2% mixed	3.5 (31.1)	MMSE=18.2 (4.8)	78.2 (8.4)	99	66.6	55.6	44.4	61.9 (11.9)	Coping strategies	Psychosis; mood/apathy; hyperactivity; overall BPSD
Wells and Over^47^	Australia	93	42	NR	5.6 (4.2)	NR	76.1 (7.3) men; 74.6 (6.2) women	93	58	100	0	74.7 (6.2) men; 71.4 (7.9) women	Coping strategies	Institutionalisation
Torossian and Ruffins^48^	USA	197	60.9	AD	NR	GDS range=4–7	NR	197	39.1	100	0	70.7	Adaptability; cohesion	Institutionalisation
Markiewicz *et al*^49^	Canada	108	56.6	92% AD, 8% dementia+stroke	3.43 (2.57)	MMSE=12.86 (7.95)	74.42 (8.37)	108	68.1	65.5	34.5	62.24 (12.97)	Attachment styles	Institutionalisation
Pruchno *et al*^50^	USA	220	NR	AD	NR	NR	NR	220	67.9	100	0	70.2; 45–94	Quality of relationship	Institutionalisation
McClendon *et al*^51^	USA	141	45	71.5% probable AD, 28.5% possible AD	4.14 (2.47)	MMSE=17.28 (6.50)	72.46 (7.91)	141	NR	75	NR	NR	Caregiver coping	Time to death
Shroff^52^	USA	NR	NR	NR	NR	NR	84.78 (7.78)	83	85.5	54.3	NR	62.38 (18.21)	Family coping and coherence	PwD QoL
Snyder^53^	USA	233	56.7	NR	3.49 (1.80)	NR	86.08 (5.83)	233	78.5	37.3	53.6	66.27 (13.21)	Carer coping strategies	Incidence of severe dementia, time to institutionalisation, mortality

*The exposures and outcomes listed in the table refer only to the ones relevant to our review question.

Study design: all studies were prospective cohorts except for Godwin (randomised trial) and Scroff (controlled before and after intervention study), for these two studies we ignored the intervention status.

AD, Alzheimer’s dementia; BPSD, Behavioural and Psychological Symptoms of Dementia; CAMCOG, Cambridge Cognition score; GDS, Global Deterioration Scale; LB, Lewy body dementia; MMSE, Mini-Mental State Examination; n, numbers in analysis; NHP, nursing home placement; NR, not reported; PwD, person with dementia; QoL, quality of life; VD, vascular dementia.

### Risk of bias in included studies

None of the studies met all eight NOS criteria. All studies had adequate ascertainment of exposure as these were all based on structured interviews. All but one also had adequate ‘demonstration that the outcome of interest was not present at start of study’ and ‘follow up long enough for outcome to occur’. Blind independent outcome assessment was not possible in many studies with institutionalisation as the outcome, because this outcome tended to be self-reported by the carer table 2.

**Table 2:** Risk of bias assessment based on Newcastle-Ottawa Scale

Study Author Year	Country	Newcastle-Ottawa Scale items addressing different methodological components of the study and potential sources of bias							
		Representativeness of the exposed cohort	Selection of the non-exposed cohort	Ascertainment of exposure	Outcome of interest was not present at start of study	Comparability of cohorts on the basis of the design/analysis (confounding)	Assessment of outcome blind/record linkage	Follow-up long enough for outcome to occur	Adequacy of follow-up of cohorts (attrition)
Pruchno *et al* 1990^50^	USA	✓	✓	✓	✓	✗*	✗	✓	✗
Vitaliano *et al* 1993^44^	USA	✓	✓	✓	✓	✗	✗	✓	✓
Wright 1994^42^	USA	✓	✓	✓	✓	✗	✓	✓	✓
Markiewicz *et al* 1997^49^	Canada	✓	✓	✓	✓	✗	✓	✓	✓
Bannister *et al* 1998^45^	UK	✓	✓	✓	✓	✗	✗	✓	✓
Wells and Over 1998^47^	Australia	✓	✓	✓	✓	✗	✗	✓	✗
Wright *et al* 1998^43^	USA	✗	✗	✓	✓	✗	✗	✓	✓
Caron *et al* 1999^36^	USA	✗	✗	✓	**✓**	**✗**	**✗**	✓	✓
Fisher and Lieberman 1999^39^	USA	✓	✓	✓	✓	✗	✗	✓	✓
Torossian and Ruffins 1999^48^	USA	✗	✓	✓	✓	✗	✗	✓	✗
Spruytte *et al* 2001^38^	Belgium	✓	✓	✓	✓	✗	✗	✗	✓
Burgener and Twigg 2002^20^	USA	✓	✓	✓	✓	✗	✗	✓	✓
de Vugt *et al* 2004^46^	Netherlands	✓	✓	✓	✗	✗	✗	✓	✓
McClendon *et al* 2004^51^	USA	✗	✓	✓	✓	✗	✓	✓	✗
Stevens *et al* 2004^40^	USA	✓	✓	✓	✓	✗	✗	✓	✓
Perren *et al* 2007^41^	Switzerland	✓	✓	✓	✓	✗	✗	✓	✓
Kunik *et al* 2010 (four papers)^32–35^	USA	✓	✓	✓	✓	✗*	✗	✓	✓
Clare *et al* 2014^37^	UK	✗	✓	✓	✓	✗	✗	✓	✗
Shroff 2015^52^	USA	✓	✓	✓	✓	✗	✗	✗	✗
Snyder 2016^53^	USA	✓	✓	✓	✓	✗*	✓	✓	✗

✓=study dealt with this adequately. ✗=study was at risk of bias in this area, or provided no information to demonstrate otherwise.

*These studies adjusted for three out of the four key confounders.

In total, 60 separate analyses were included in this review, of which 40 (two-thirds) did not control for any potential confounding factors. In the 20 analyses that did include some adjustment, only a minority adjusted for any of the factors we identified as key potential confounders. None of the studies adjusted for all four key confounding factors. Three studies each adjusted for three out of the four key confounding factors.^33 50 53^ A further two studies adjusted for two of the four key confounders.^46 47^

Seven studies had inadequate follow-up of the cohort (loss to follow-up).^37 47 48 50–53^ Many studies presented insufficient information to make a clear judgement on some of the NOS criteria. In addition to potential risk of bias, many studies had reporting problems. Of the 60 included results, eight (13%) neither reported effect size estimates nor CIs, 40 (67%) reported an effect size with no CIs and 23 (38%) did not report specific P values. Only six analyses (10%) fully reported their results with effect size, CIs and P values.

### Primary outcome: risk factors for institutionalisation

Ten studies examined 25 different relationship quality factors as potential risk factors for institutionalisation. Follow-up ranged from 6 to 24 months. The majority of studies found no association between the risk factors investigated and the incidence of institutionalisation. Although some individual studies reported associations between relationship quality and institutionalisation, there were no consistent findings across risk factors, and the lack of appropriate adjustment for basic confounding factors makes interpreting the results very difficult table 3.

**Table 3:** Associations between relationship factors and institutionalisation (10 studies)

Risk factor	Study	n	Follow-up	Results (95% CI; P value)	Analysis adjusted for*
Factors relating to the interaction between the person with dementia and their caregiver					
Quality of relationship	Spruytte *et al*^38^	144	6–9 m	OR 0.92 (P=0.02)	NR
	Pruchno *et al*^50^	220	12 m	OR 1.31 (P>0.05)	a, g, s, r, t, c, bp, ADL, ci, m, hs, cb
Marital happiness At baseline	Wright^42^	29	24 m	PoV 0.313 (P<0.01)	NR
Before dementia onset				PoV 0.045 (P>0.05)	NR
Emotional closeness	Fisher and Lieberman^39^	164	24 m	OR 1.64 (1.09, 2.46; P=0.02)	d, ba, nf, oc, fe
Emotional distancing/boundary ambiguity	Fisher and Lieberman^39^	164	24 m	OR 1.25 (0.85, 1.83; P=0.26)	d, ba, nf, oc, fe
	Wells and Over^47^	93	12–18 m	OR 1.3 (P>0.05)	a, d, t
Cohesion	Torossian and Ruffins^48^	197	24 m	MD NR (P>0.05)	NR
	Wright^42^	29	24 m	PoV 0.472 (P<0.001)	NR
Affection	Wright	29	24 m	PoV 0.144 (P>0.05)	NR
Warmth†	Spruytte *et al*^38^	144	6–9 m	NR (P<0.05)	NR
Anxious-ambivalent attachment (carer)	Markiewicz *et al*^49^	108	24 m	OR 1.27 (P=0.36)	NR
Avoidant attachment (carer)	Markiewicz *et al*^49^	108	24 m	OR 2.39 (P<0.001)	NR
	Stevens *et al*^40^	215	24 m	HR 1.011 (P=0.37)	NR
High levels of tension	Wright^42^	29	24 m	PoV 0.162 (P>0.05)	NR
Excessive criticism*†	Spruytte *et al*^38^	144	6–9 m	NR (P>0.05)	NR
Factors mainly relating to the caregiver					
Limited adaptability	Torossian and Ruffins^48^	197	24 m	MD NR (P>0.05)	NR
Approach coping	Stevens *et al*^40^	215	24 m	HR 0.997 (P=0.77)	NR
Directing relative’s behaviour	Bannister *et al*^45^	116	12 m	MD 0.2 (P=0.40)	NR
Keeping relative busy	Bannister *et al*^45^	116	12 m	MD 0.5 (P=0.02)	NR
Learning about the illness	Bannister *et al*^45^	116	12 m	MD 0.3 (P=0.42)	NR
Prioritising	Bannister *et al*^45^	116	12 m	MD 0.1 (P=0.52)	NR
Reducing expectations	Bannister *et al*^45^	116	12 m	MD 0.4 (P=0.19)	NR
Consistent larger sense of the illness	Bannister *et al*^45^	116	12 m	MD 0.4 (P=0.35)	NR
Positivity	Bannister *et al*^45^	116	12 m	MD 0.5 (P=0.57)	NR
	Wells and Over^47^	93	12–18 m	OR 1.03 (P>0.05)	a,d
Seeking social support	Wells and Over^47^	93	12–18 m	OR 1.91 (P<0.05)	a,d
	Snyder^53^	233	NR	HR 1.159(0.718 to 1.87; P=0.55)	a, d, g, nc
Accepting responsibility	Wells and Over^47^	93	12–18 m	OR 0.28 (P<0.01)	a, d
Confrontational	Wells and Over^47^	93	12–18 m	OR 2.12 (P<0.05)	a, d
Negative feelings	Fisher and Lieberman^39^	164	24 m	OR 1.47 (0.99 to 2.19; P=0.05)	d, ba, oc, ec, fe

*Prespecified key confounders: a, age; d, dementia severity; g, gender; s, socioeconomic status. All other confounders: ADL, activities of daily living; ba, boundary ambiguity; bp, behaviour problems; c, number of children; cb, caregiver burden; ci, caregiver illness; ec, emotional closeness; fe, family efficiency; hs, help services used; m, medication; nc, non-coresidency; nf, negative feeling; oc, organised cohesiveness; r, religion; t, time spent (duration of) caregiving.

†These are the subscales of the relationship quality scale.

HR > 1 indicates increased risk, HR < 1 indicates decreased risk. OR > 1 indicates increased odds of outcome, OR < 1 indicates decreased odds of outcome.

Corr, correlation (This is a number between −1 to +1 and indicates the degree to which the exposure and outcome vary together. Positive numbers indicate that exposure and outcome increase together, while negative numbers indicate that the outcome increases as the exposure decreases. Larger numbers indicate stronger correlation); MD, mean difference in outcome (Large differences between groups suggests that the exposure might affect the outcome. For continuous exposures, mean difference represents the change in the outcome for one unit increase in the exposure.); NR, information not reported (where possible results and 95% CIs were calculated from raw data); PoV, proportion of variance (This is a number between 0–1 indicating the proportion of variance in the outcome explained by the exposure. Higher numbers indicate greater explanatory power of the exposure.)

### Risk factors for challenging behaviour (BPSD)

Under the umbrella term of challenging behaviour four main types of outcome were evaluated: global challenging behaviour scores, psychotic symptoms, depression and other BPSD outcomes. Eight studies examined nine different relationship quality factors as potential risk factors for these aspects of challenging behaviour. The length of follow-up ranged from 6 to 24 months table 4.

**Table 4:** Associations between relationship factors and challenging behaviour (BPSD) (eight studies)

Risk factor	Study	n	Follow-up	Results (95% CI; P value)	Analyses adjusted for*	Outcome assessment tool, scale range
Outcome: global challenging behaviour (BPSD) or problem behaviour (four studies)						
Emotional distancing/boundary ambiguity	Caron *et al*^36^	60	12 m	Corr 0.27 (P<0.001) (overall)	NR	BEHAVE-AD, 0–75
Non-adaptive coping	de Vugt *et al*^46^	69	6m	MD NR (P=0.007)	g, s, ct	NPI, 1–144
		69	12 m	MD NR (P=0.019)	g, s, ct	
Avoidant attachment	Perren *et al*^41^	68	24 m	MD 0.23 (P<0.05)	NR	NPI, 1–144
Expressed emotion	Vitaliano *et al*^44^	77	15–18 m	MD 1.9 (0.77 to 3.04; P<0.001)	NR	SCB, 0–8
Outcome: psychotic symptoms (three studies)						
Mutuality	Ball *et al*^33^	171	24 m	MD −0.1 (−0.24 to 0.04; P>0.05) (delusions)	a, g, d, e	NPI, 1–12
	Ball *et al*^33^	171	24 m	MD −0.04 (−0.16 to 0.08; P>0.05) (hallucinations)	a, g, d, e	
Emotional distancing/boundary ambiguity	Caron *et al*^36^	60	12 m	Corr 0.30 (P<0.001) (paranoia)	NR	BEHAVE-AD, 0–75
Non-adaptive coping	de Vugt *et al*^46^	69	12 m	MD NR (P>0.05) (psychosis)	g, s	NPI, 1–24
Outcome: depression (five studies)						
Mutuality	Ball *et al*^33^	171	24 m	MD −0.43 (−0.80 to -0.06; P<0.05)	a, g, d, e	HAM-D, 0–68
Quality of relationship	Burgener and Twigg^20^	70	18 m	Corr 0.22 (P>0.05)	NR	Cornell, 0–38
Emotional distancing/boundary ambiguity	Caron *et al*^36^	60	12 m	Corr 0.01 (P>0.05)	NR	BEHAVE-AD, 0–75
Expressed emotion	Vitaliano *et al*^44^	77	15–18 m	MD 2.00 (−0.31 to 4.31; P>0.05)	NR	HAM-D, 0–68
Cohesion quantity	Wright *et al*^43^	14	6m	Corr −0.302 (P>0.05)	NR	Zung, 0–270
Quality		14	6m	Corr −0.387 (P>0.05)	NR	
Affection quantity	Wright *et al*^43^	14	6m	Corr −2.41 (P>0.05)	NR	
Quality		14	6m	Corr −0.038 (P>0.05)	NR	
Tension quantity	Wright *et al*^43^	14	6m	Corr 0.533 (P<0.01)	NR	
Quality		14	6m	Corr −0.288 (P>0.05)	NR	
Outcome: other						
Non-adaptive coping	de Vugt *et al*^46^	69	12 m	MD NR (P=0.512) (apathy)	g, s, ct	NPI, 1–48
Non-adaptive coping	de Vugt *et al*^46^	69	12 m	MD NR (P=0.005) (hyperactivity)	g, s, ct	NPI, 1–60
Mutuality	Morgan *et al*^34^ and Kunik *et al*^32^	171	24 m	MD −0.42 (−0.64 to 0.20; P<0.001) HR 0.63 (0.45 to 2.87; P=0.006) (aggression)	NR	CMAI, 0–156
Emotional distancing/boundary ambiguity	Caron *et al*^36^	60	6m	Corr 0.15 (P>0.05) (anxiety)	NR	BEHAVE-AD, 0–75

*Prespecified key confounders: a, age; d, dementia severity; g, gender; s, socioeconomic status. All other confounders: ct, carer type; e, ethnicity.

HR > 1 Indicates increased risk, HR < 1 indicates decreased risk. OR > 1 indicates increased odds of outcome, OR < 1 Indicates decreased odds of outcome.

BEHAVE-AD, Behavioural Pathology in Alzheimer’s Disease Rating Scale; BPSD, Behavioural and Psychological Symptoms of Dementia; CMAI, Cohen Mansfield Agitation Inventory; Corr, correlation (This is a number between −1 to +1 and indicates the degree to which the exposure and outcome vary together. Positive numbers indicate that exposure and outcome increase together, negative numbers indicate that the outcome increases as the exposure decreases. Larger numbers indicate stronger correlation); Cornell, Cornell Scale for Depression in Dementia; HAM-D, Hamilton Rating Scale for Depression;MD, mean difference in outcome (Large differences between groups suggests that the exposure might affect the outcome. For continuous exposures, mean difference represents the change in the outcome for one unit increase in the exposure); NPI, Neuropsychiatric Inventory; NR, information not reported (where possible results and 95%CIs were calculated from raw data); PoV, proportion of variance (This is a number between 0–1 indicating the proportion of variance in the outcome explained by the exposure. Higher numbers indicate greater explanatory power of the exposure); SCB, Screen for Caregiver Burden (selection of items); Zung, Short Zung Interviewer Assisted Depression Rating Scale.

There was a suggestion of an association between relationship factors and global challenging behaviour. All studies evaluating global challenging behaviour provided statistical evidence of an association (most P values below 0.02). However, one study that reported two analyses did not report effect sizes.^46^ For another, the reported effect size was very small (mean difference of 0.23 on a scale of 1–144).^41^ A larger effect size was seen for the association between EE and global challenging behaviours (mean difference of 1.9 in a scale of 0–8).^44^ None of these analyses adjusted for our prespecified confounding factors.

Most studies found no evidence of an association between relationship factors and either psychotic symptoms, depression or other BPSD outcomes. However, some of these were small studies that may have been underpowered to detect an association. One study adjusted for three out of four prespecified potential confounders.^33^ This was also one of the largest studies (n=171). It found no evidence for an association between couple mutuality and psychotic symptoms and a very weak, likely clinically negligible effect of this factor on depression (mean difference −0.43 points on a scale of 0–68).^33^

### Risk factors for hospitalisation, QoL and death

The outcomes of hospitalisation^35^ were examined in one study, and QoL^37 52^ and death^51 53^ were each examined in two studies. The small number of poor quality studies means it is not possible to draw conclusions regarding the association of relationship factors with these outcomes table 5.

**Table 5:** Associations between relationship factors and hospitalisation, quality of life and time to death

Risk factor	Study	n	Follow-up	Results (95% CI; P value)	Analyses adjusted for*
Hospitalisation (one study)					
Relationship strain	Godwin *et al*^35^	296	12 m	OR 1.03 (0.92 to 1.14; 0.637)	NR
Quality of life (two studies)					
Quality of relationship (patient view)	Clare *et al*^37^	51	20 m	MD 0.31 (P=0.06)	pqol, pd, cs, cqor
	Shroff^52^	83	NR	R^2^ 0.179 (P<0.001)	NR
Quality of relationship (carer view)	Clare *et al*^37^	51	20 m	MD −0.13 (P=0.89)	pqol, pd, cs, cqor
Time to death (two studies)					
Instrumental coping	McClendon *et al*^51^	141	5–9 years	HR 0.99 (P=0.915)	NR
Acceptance coping	McClendon *et al*^51^	141	5–9 years	HR 1.09 (P=0.644)	NR
Wishful thinking	McClendon *et al*^51^	141	5–9 years	HR 1.41 (P=0.019)	NR
	Snyder^53^	233	NR	HR 0.88 (0.673 to 1.171; 0.4)	a, d, g, nc
Problem focused coping	Snyder^53^	233	NR	HR 0.80 (0.571 to 1.128; 0.2)	a, d, g, nc
Seeking social support	Snyder^53^	233	NR	HR 1.056 (0.787 to 1.416; 0.7)	a, d, g, nc
Blaming self	Snyder^53^	233	NR	HR 0.967 (0.768 to 1.218; 0.7)	a, d, g, nc
Avoidance coping	Snyder^53^	233	NR	HR1.021 (0.720 to 1.448; 0.9)	a, d, g, nc
Blaming others	Snyder^53^	233	NR	HR 0.867 (0.632 to 1.190; 0.3)	a, d, g, nc
Counting blessings	Snyder^53^	233	NR	HR 0.648 (0.454 to 0.926; 0.017)	a, d, g, nc
Religiosity	Snyder^53^	233	NR	HR 0.882 (0.682 to 1.142; 0.341)	a, d, g, nc

*Prespecified key confounders: a, age; d, dementia severity; g, gender; s, socioeconomic status. All other confounders: cqor, carer quality of relationship; cs, carer stress; nc, non-coresidency; pd, PWD depression; Pqol, PWD quality of life.

HR > 1 indicates increased risk, HR < 1 indicates decreased risk. OR > 1 indicates increased odds of outcome, OR <1 indicates decreased odds of outcome.

MD, mean difference in outcome (Large differences between groups suggests that the exposure might affect the outcome. For continuous exposures, mean difference represents the change in the outcome for one unit increase in the exposure); NR, information not reported (where possible results and 95% CIs were calculated from raw data).

## Discussion

This systematic review assessed the evidence on the role of relationship factors on outcomes in dementia. Although it is plausible that relationship factors could affect the risk of institutionalisation, challenging behaviour and other outcomes, there is currently no robust evidence to establish which, or to what extent, elements of the caring relationship affect specific outcomes.

The majority of studies found no association between specific risk factors and the outcomes of interest. However, this is not necessarily evidence of a lack of association, as some of the studies will have been underpowered to detect differences, and most were at risk of confounding as they failed to adjust for even the most basic confounding factors. There was a suggestion of an association between factors related to the emotional withdrawal of the caregiver and subsequent increased risk of challenging behaviour in the person with dementia. All studies in this category found statistical evidence of an association. However, the methodological quality of these studies was poor. For example, many did not report effect sizes, while in others the effect sizes were small, suggesting that associations may not be clinically important. This could also be because the sample was too small to detect a difference. No study reported justification for their sampling or the sample sizes. There was also a potential for confounding in studies that did report effect sizes.

One of the strengths of this review is a thorough, sensitive search that will have minimised the chance of missing relevant studies. By limiting inclusion to cohort studies only, we avoided interpretive difficulties from recall bias (systematic differences in how people remember risk factors) and reverse causation (when a purported risk factor is in fact a result of the outcome). The double-screening of each record by two reviewers working independently also minimised the possibility of errors and selection bias in the identification of eligible reports. All data extracted was checked in full by a second reviewer.

A limitation of our review is that we were not able to assess for publication bias or selective reporting of results. It is known that studies with null findings are less likely to be published, and authors can selectively report their ‘more interesting’ findings.^54^ This means it is possible there are other relevant results that have not been reported and so would not be available to a review. We attempted to minimise the risk of publication bias by including a grey literature search.

Our ability to answer the research question was limited due to unclear and incomplete reporting, few studies assessing similar risk factors and the potential for bias in included studies. We considered the appropriateness of using meta-analysis to produce summary estimates. However, many studies did not report full details of effect sizes and CIs that are required for meta-analysis. Additionally, most risk factor–outcome relationships were only reported in one or two studieswith conflicting results or non-comparable outcome measures between studies), thus themeta-analyses would have been inapropriate. It would not have been meaningful or appropriate to combine different risk factors or outcomes in pooled analysis. We assessed the potential for bias in included studies and took these assessments into consideration when reaching conclusions, thus avoiding overinterpretation of findings from studies with potentially serious problems.

Very few studies adjusted their analyses for key prespecified confounding factors (age, gender, SES and dementia severity). Two-thirds of included analyses did not adjust for any confounding factors. This is an important limitation as it means any findings could be explained by differences in these factors across the exposure groups—differences that are very likely in a non-randomised study where participants self-select into exposure groups. A small number of studies were adjusted for other factors, but these were factors such as QoL and emotional withdrawal that may well be on the causal pathway between the relationship factor studied and the outcome (so not a true confounder). Reporting of results was incomplete with many studies only reporting P values, or just that an association was ‘statistically significant’/‘not significant’ with no effect sizes or CIs. This is another important limitation as in these cases we have no information about the probable magnitude of effect, and it is impossible to interpret the potential clinical implications of any statistical difference. Inadequate reporting of methods and results made it difficult to assess risk of bias. Very few studies evaluated the same risk factors, so there is also very little evidence on any individual factor. Duration of follow-up (typically 6–24 months), although theoretically long enough for an outcome to have occurred, may not have been sufficient to detect outcomes in the majority of the sample in a study. As dementia is typically a slowly progressing disease, this may not be sufficient for long-term outcomes such as institutionalisation.

Finally, majority of the studies identified were published before 2000. The most recent study is from 2016, with only three studies reported in the last 5 years. The reasons for this apparent diminishing of literature are unclear, but the relative absence of more recent studies may have implications for applicability of the findings of this review. New long-term studies that are well conducted and fully reported are needed to reliably answer questions regarding effect of family relationship quality on institutionalisation risks.

The current evidence does not provide a basis by which general practitioners or other health professionals could reliably identify people at risk of poor outcome on the basis of relationship factors. However, the lack of robust evidence about the role of relationship factors does not imply that personal relationships are not important factors in dementia outcomes; many professionals working with families consider these to be important.^55 56^ One plausible mechanism is that factors such as emotional withdrawal of the caregiver might prompt ‘challenging behaviour’ in the person with dementia as an attempt to elicit an emotional connection.^41^ For instance, caregiver-coping strategies such as constantly ‘correcting’ the person with dementia, rather than accepting their cognitive challenges, could be construed as potentially undermining the individual’s personhood or self-esteem, both of which are seen as key elements in good dementia care. In this way, any ‘challenging behaviour’ might be construed as behavioural expressions of underlying frustration or distress. It is also possible that challenging behaviours are themselves on the causal pathway between relationship factors and institutionalisation—the latter plausibly becoming more likely as the caregiver becomes less able to cope with challenging behaviours. If this is the case, then longer follow-up may be necessary to detect associations between relationship factors and institutionalisation. In addition, the association between quality of relationships, challenging behaviour and subsequent institutionalisation is likely to be complex. A recent study^54^ found that relationship quality was one of a number of psychosocial factors associated with caregiver distress at challenging behaviour independently of the frequency of that behaviour. Similarly, changes in the meaning of their relationship, and in particular the belief that their relative had lost, or would inevitably lose, their identity to dementia, is a fundamental reason why family carers experienced behaviour as challenging.^55^ It may be, then, that the quality of relationship acts as a confounding variable in the association between challenging behaviour and institutionalisation.

Lack of evidence on what relationship factors predict outcomes in people with dementia should not preclude further evaluation of psychosocial interventions targeting people with dementia and their carers. Such studies could use experimental designs to identify which interventions work and in which settings. It may also be useful to look to qualitative research, exploring the views of people with dementia and their family carers on what they consider important for continued living at home and the challenges in the relationship that they face.

## Conclusions

There is currently no strong or consistent evidence on the effects of relationship factors on institutionalisation, hospitalisation, death or QoL for people with dementia. There was a suggestion of an association between relationship factors and challenging behaviour, although the evidence for this was weak. As the current focus of dementia care is ‘person-centred’, which prioritises interpersonal relationships, this lack of evidence about the role of relationships in dementia outcomes is striking. To improve our ability to support those with dementia and their families, this evidence gap needs to be addressed.

## Supplementary Material

Reviewer comments

Author's manuscript
