# Supplementary material for: Quality of family relationships and outcomes of dementia: a systematic review
Source: BMJ Open. 2018 Jan 21;8(1):e015538. doi: 10.1136/bmjopen-2016-015538 (PMC5780722; doi:10.1136/bmjopen-2016-015538)
Supplement: Author's manuscript [file bmjopen-2016-015538.draft_revisions.pdf]

BMJ Open

BMJ Open is committed to open peer review. As part of this commitment we make the peer review history of every article we publish publicly available.

When an article is published we post the peer reviewers' comments and the authors' responses online. We also post the versions of the paper that were used during peer review. These are the versions that the peer review comments apply to.

The versions of the paper that follow are the versions that were submitted during the peer review process. They are not the versions of record or the final published versions. They should not be cited or distributed as the published version of this manuscript.

BMJ Open is an open access journal and the full, final, typeset and author-corrected version of record of the manuscript is available on our site with no access controls, subscription charges or pay-per-view fees (http://bmjopen.bmj.com).

If you have any questions on BMJ Open's open peer review process please email editorial.bmjopen@bmj.com

BMJ Open

Quality of family relationships and outcomes of dementia: a systematic review

Journal:	*BMJ Open*
Manuscript ID	bmjopen-2016-015538
Article Type:	Research
Date Submitted by the Author:	12-Dec-2016
Complete List of Authors:	Edwards, Hannah; University of Bristol, School of Social and Community Medicine<br>Whiting, Penny; University of Bristol, School of Social and Community Medicine<br>Ijaz , Sharea ; NIHR CLAHRC West<br>Leach, Verity; University of Bristol<br>Richards, Alison; University of Bristol, School of Social and Community Medicine<br>Cullum, Sarah; University of Bristol, School of Social and Community Medicine<br>Cheston, Richard; University of the West of England, Health and Social Sciences<br>Savovic, Jelena; University of Bristol, NIHR CLAHRC West
**Primary Subject Heading**:	Neurology
Secondary Subject Heading:	Evidence based practice, Health services research, Patient-centred medicine, Epidemiology
Keywords:	Dementia < NEUROLOGY, family relationships, challenging behaviour, institutionalisation, systematic review

SCHOLARONE™
Manuscripts

Title

Quality of family relationships and outcomes of dementia: a systematic review

Author details

Corresponding author: Hannah B Edwards: School of Social and Community Medicine, University of Bristol, and National Institute for Health Research (NIHR) Collaboration for Leadership in Applied Health Research and Care (CLAHRC) West.

Email: hannah.edwards@bristol.ac.uk

Telephone: 0117 342 7691

Address: 9th Floor Whitefriars, Lewins Mead, Bristol BS1 2NT, UK

ORCID: 0000-0002-1885-4771

Full author list: Hannah B Edwards[1,2], Penny Whiting[1,2], Sharea Ijaz[1,2], Verity Leach[1,2], Alison Richards[1,2], Sarah Cullum[3], *Richard Cheston[4], *Jelena Savović[1,2]

1. School of Social and Community Medicine, University of Bristol. Bristol, UK
2. National Institute for Health Research (NIHR) Collaboration for Leadership in Applied Health Research and Care (CLAHRC) West. Bristol, UK
3. University of Auckland. Auckland, New Zealand.
4. Health and Social Sciences, University of the West of England. Bristol, UK

* Joint senior authors

Keywords: Dementia, carers, family relationships, institutionalisation, challenging behaviour

Word count: 3389

Tables: 4

Figures: 1

Appendices: 1

For peer review only

For peer review only - http://bmjopen.bmj.com/site/about/guidelines.xhtml

ABSTRACT

Objectives: Evaluation of the association between the quality of relationship between a person with dementia and their family carer and outcomes for the person with dementia.

Design: Systematic review.

Eligibility criteria: Cohort studies of people with clinically diagnosed dementia and their main carers. Exposures of interest were any elements of relationship quality, e.g. attachment style, expressed emotion, coping style. Our primary outcome was institutionalisation, and secondary outcomes were hospitalisation, death, quality of life, and behavioural and psychiatric symptoms of dementia (‘challenging behaviour’).

Data sources: Medline, Embase, Web of Science, PsycInfo, the Cochrane Library and Opengrey were searched from inception to May 2015.

Study appraisal and synthesis methods: The Newcastle-Ottawa Scale was used to assess risk of bias. A narrative synthesis of results was performed due to differences between studies.

Results: Eighteen studies were included. None of the studies controlled for all pre-specified confounding factors (age, gender, socio-economic status, and severity of dementia). Reporting of results was poor with many studies simply reporting whether or not associations were ‘statistically significant’ without providing effect size estimates or confidence intervals. There was a suggestion of an association between relationship factors and global challenging behaviour. All studies evaluating global challenging behaviour provided statistical evidence of an association (most p-values below 0.02). There was no consistent evidence for an association for any other outcome assessed.

Conclusions: There is currently no strong or consistent evidence on the effects of relationship factors on institutionalisation, hospitalisation, death or quality of life for people with dementia. There was a suggestion of an association between relationship factors and challenging behaviour, although the evidence for this was weak. To improve our ability to support those with dementia and their families further robust studies are needed.

Systematic review registration: CRD42015020518

For peer review only - http://bmjopen.bmj.com/site/about/guidelines.xhtml

For peer review only

ARTICLE SUMMARY

Strengths and limitations of this study

- Broad search strategy so unlikely to have missed relevant studies
- Double screening minimises selection bias
- We were not able to assess publication bias and the potential for selective reporting of outcomes within studies.

For peer review only

BACKGROUND

Dementia is a key public health concern in the UK[1-3] with around 7% of all those over 65 affected, and the numbers of people with dementia predicted to double every 20 years.[4-6] Institutionalisation (being placed in a full time care /nursing home) is a key outcome for people with dementia, their families, and the healthcare system. Although in many circumstances institutionalisation may be the best or only option for the person affected by dementia, most people report that they would prefer to stay living in their own home.[7] Recent media attention to a few very poorly run care homes has also lead to concerns about institutionalisation.[8-10] Additionally the financial cost of full-time care is very high, both for affected individuals and their families, and for the public, as public taxes are used to contribute to care home fees. Consequently, for some time it has been UK government policy to help families to continue supporting people with dementia at home, specifically to delay or avoid institutionalisation.[11]

The quality of relationship between the person with dementia and people who care for them has been linked with a range of outcomes including institutionalisation, cognitive and functional decline, and quality of life.[12-21] There is also growing interest in the potential for psychosocial interventions to improve outcomes, by enhancing interactions within families.[22,23] If families are better equipped to cope with the psychological and emotional challenges of dementia, then care at home may be

For peer review only - http://bmjopen.bmj.com/site/about/guidelines.xhtml

sustained for longer. However, it is not clear which elements of the relationship are predictive of early institutionalisation or which lead to a faster decline. This evidence is necessary both to justify and to help to develop early psychosocial interventions, ideally at the point of diagnosis.

To address this issue we performed a systematic review of the evidence on how elements of relationship quality between the person with dementia and their main informal (family) carer are associated with outcomes for the person with dementia.

METHODS

This study was a systematic review, registered with the PROSPERO International Prospective Register of systematic reviews, registration number CRD42015020518. The full protocol has been published.[24]

Eligibility criteria

Only cohort studies (prospective and retrospective) were included in the review. Relevant systematic reviews were obtained and used as a means of identifying other original studies.[24]

The population of interest was people with dementia and their main informal caregiver (most commonly a spouse or child). Professional, paid caregivers were excluded. People with all types of clinically-diagnosed dementia were included.

The exposures of interest were factors that capture an element of relationship quality. Attachment style, coping style, affection, and expressed emotion were all identified at the design stage as key exposures of interest. While coping and affection are relatively straightforward terms, attachment style and expressed emotion relate to specific psychological constructs. 'Attachment style' is a term

For peer review only - http://bmjopen.bmj.com/site/about/guidelines.xhtml

originally developed to understand the emotional relationship between children and parents, but has since been extended to adult romantic relationships. Four main styles of attachment have been identified in adults: secure; anxious–preoccupied; dismissive-avoidant; and fearful-avoidant.[25,26] 'Expressed Emotion' (EE) is a measure of the family environment based on how the relatives of a psychiatric patient spontaneously talk about/to the patient. High levels of expressed emotion have been associated with worse prognosis in a number of mental illnesses including schizophrenia. This may be due to emotional over-involvement, which can be experienced as hostility, criticism and intolerance.[27,28]

Two other notable factors emerged as key exposures when reviewing the literature: 'mutuality' and 'boundary ambiguity'. Mutuality is a cluster concept capturing levels of positive engaging interaction, attachment and emotional support. Boundary ambiguity involves uncertainty about whether a person is in or out of the family group. This occurs as a result of significant changes in that person including those cognitive, functional, mood and personality changes that are indicative of dementia. Boundary ambiguity is associated with, and taken as an indicator of emotional distancing and the withdrawal of the caregiver from the person with dementia. Other factors emerging from the literature were included if they captured an element of relationship quality, and this was assessed on a case-by-case basis through discussion with the study team. Carer abuse was excluded, as this was considered to be a different area of research. Overall measures of carer burden were also excluded as an exposure because they relate more to cognitive and functional levels in dementia than to relationship quality.

The primary outcome of interest was institutionalisation. This is a key event in the course of dementia, both socially and financially, from the perspectives of the individual, their family and the public. Secondary outcomes were hospitalisations, death (or time to death), quality of life (QoL), and challenging behaviour (also referred to as the behavioural and psychological symptoms of dementia

For peer review only - http://bmjopen.bmj.com/site/about/guidelines.xhtml

or BPSD). Examples of challenging behaviour can include depression, anxiety, aggression, paranoia, hallucinations and delusions. Studies measuring QoL or challenging behaviour as the outcome had to use validated assessment tools to be included in the review.

Search strategy

Medline, Embase, Web of Science, PsycInfo, The Cochrane Library and Opengrey were searched from inception to May 2015 (see Appendix A for full search strategy). All results were imported into an Endnote X7 reference library, and into a bespoke-built Microsoft Access 2013 database to manage screening.

Selection of papers

The titles and abstracts of all identified papers were screened in duplicate by two reviewers working independently, and all potentially relevant papers were retrieved. All retrieved papers were read in full and assessed for eligibility using a standardised and piloted inclusion checklist, applied by two reviewers independently. Any discrepancies between the reviewers (at either stage of screening) were resolved through discussion.

Data extraction

Data were extracted from included studies using a bespoke data collection form, which was piloted on six studies and amended as a result of the piloting. Data extracted included study characteristics, characteristics of the population of people with dementia and their carers, recruitment and response, the exposures and outcomes and how and when they were measured, and details of the analyses and results. The terminology for risk factors and outcomes was used as reported by the original study authors. Where numerical results were incompletely reported, where possible, relevant results (effect estimates, standard errors, 95% confidence intervals) were calculated from the raw data. Multiple publications of the same dataset were counted as a single study. In the case

For peer review only - http://bmjopen.bmj.com/site/about/guidelines.xhtml

where analyses of the same association was repeated in more than one report, our policy was either to include the result based on the largest sample size only, or if sample sizes were equivalent, then we would include the result from the most recent publication only. Data were extracted from the published reports only, we did not contact authors for additional unpublished information.

Analysis

We planned to use meta-analysis to estimate summary effect sizes if there had been sufficient studies with similar populations, exposures and outcomes. As meta-analysis was not possible, a narrative synthesis of results is provided.[24]

Assessment of methodological quality and risk of bias

The Newcastle-Ottawa Scale[29] (NOS) was used to assess risk of bias for included reports. This is an eight-item questionnaire that assesses the following methodological criteria: representativeness of the exposed cohort; selection of the non-exposed cohort; ascertainment of exposure; demonstration that the outcome of interest was not present at the start of the study; comparability of cohorts (risk of confounding); assessment of exposure and outcome; and length and adequacy of follow up. NOS allocates 'stars' for adequate methods, but does not specifically advise calculating the sum of allocated stars to give an overall score.[29] Empirical evidence also suggests that numerical quality scores are not helpful in differentiating between studies of high and low risk of bias.[30] For this reason we considered each of the eight criteria of the NOS tool separately and assessed the study as having adequate methods for that particular aspect of study conduct if the 'star' could be allocated for that NOS criterion. To be assessed as adequate for comparability of cohorts (risk of confounding) a study had to control for the following four pre-specified factors which we viewed as likely key potential confounders: age, gender, socio-economic status (SES) and dementia severity.[24]

For peer review only - http://bmjopen.bmj.com/site/about/guidelines.xhtml

RESULTS

The search identified 7,684 potentially relevant papers. Of these, 162 papers were retrieved for full-text screening, 21 publications met the eligibility criteria (Figure 1). Four of the 21 publications were based on data from one cohort study[31-34] and so the total number of unique studies was 18 (2,107 participants). As the four connected publications each contributed unique results, all were included in the review.

Study characteristics

The majority of included unique studies (12 out of 18) came from the US, and there was one each from the UK, Canada, Switzerland, Belgium, the Netherlands, and Australia. Sample sizes in the relevant analyses ranged from 29 to 220 (mean 143). Years of publication ranged from 1990 to 2014, with most from the 1990s and early 2000s. The most frequently reported dementia type was Alzheimer's disease, although in six studies the distribution of dementia types was not reported. Time since diagnosis ranged from 3 months to 6.5 years, although in six studies this was not reported. The majority (13/18) did not report participants' ethnicity, where this was reported, cohorts were predominantly Caucasian. The majority of caregivers included were spouses (100% in seven studies, and more than 50% in another seven). Other carers were children of the person with dementia.

Risk of bias in included studies (Table 1)

None of the studies met all eight NOS criteria. All studies had adequate ascertainment of exposure as these were all based on structured interviews. All but one also had adequate 'demonstration that the outcome of interest was not present at start of study' and 'follow up long enough for outcome to occur'. Blind independent outcome assessment was not possible in many studies with institutionalisation as the outcome as this outcome tended to be self-reported by the carer.

For peer review only - http://bmjopen.bmj.com/site/about/guidelines.xhtml

In total, 60 separate analyses were included in this review, of which 40 (two thirds) did not control for any potential confounding factors. In the 20 analyses that did include some adjustment, only a minority adjusted for any of the factors we identified as key potential confounders. None of the studies adjusted for all four key confounding factors. Two studies each adjusted for three out of the four key confounding factors.[32,35] A further two studies adjusted for two of the four identified key confounders.[36,37]

Six studies had inadequate follow up of the cohort (loss to follow up). Many studies presented insufficient information to make a clear judgment on some of the NOS criteria. In addition to potential risk of bias, many studies had reporting problems. Of the 60 included results, eight (13%) neither reported effect size estimates nor confidence intervals, 40 (67%) reported an effect size with no confidence intervals, and 23 (38%) did not report specific p-values. Only six analyses (10%) fully reported their results with effect size, confidence intervals, and specific p-values.

For peer review only

For peer review only - http://bmjopen.bmj.com/site/about/guidelines.xhtml

Table 1: Risk of bias assessment based on Newcastle –Ottawa scale

Study Author Year	Country	Newcastle-Ottawa Scale items addressing different methodological components of the study and potential sources of bias							
		Representativeness of the exposed cohort	Selection of the non-exposed cohort	Ascertainment of exposure	Outcome of interest was not present at start of study	Comparability of cohorts on the basis of the design/analysis (confounding)	Assessment of outcome blind/ record linkage	Follow-up long enough for outcome to occur	Adequacy of follow up of cohorts
Pruchno 1990 [35]	USA	✓	✓	✓	✓	×[1]	×	✓	×
Vitaliano 1993 [38]	USA	✓	✓	✓	✓	×	×	✓	✓
Wright 1994 [39]	USA	✓	✓	✓	✓	×	✓	✓	✓
Markiewicz 1997 [40]	Canada	✓	✓	✓	✓	×	✓	✓	✓
Bannister 1998 [41]	UK	✓	✓	✓	✓	×	×	✓	✓
Wells 1998 [36]	Australia	✓	✓	✓	✓	×	×	✓	×
Wright 1998 [42]	USA	×	×	✓	✓	×	×	✓	✓
Caron 1999 [43]	USA	×	×	✓	✓	×	×	✓	✓
Fisher 1999 [44]	USA	✓	✓	✓	✓	×	×	✓	✓
Torrossian 1999 [45]	USA	×	✓	✓	✓	×	×	✓	×
Spruytte 2001 [46]	Belgium	✓	✓	✓	✓	×	×	×	✓
Burgener 2002 [20]	USA	✓	✓	✓	✓	×	×	✓	✓
de Vugt 2004 [37]	Netherlands	✓	✓	✓	×	×	×	✓	✓
McClendon 2004 [47]	USA	×	✓	✓	✓	×	✓	✓	×
Stevens 2004 [48]	USA	✓	✓	✓	✓	×	×	✓	✓
Perren 2007 [49]	Switzerland	✓	✓	✓	✓	×	×	✓	✓
Kunik 2010 (4 papers) [31-34]	USA	✓	✓	✓	✓	×[1]	×	✓	✓
Clare 2014 [50]	UK	×	✓	✓	✓	×	×	✓	×

✓= study dealt with this adequately. ×= study was at risk of bias in this area, or provided no information to demonstrate otherwise.

[1.] These studies adjusted for three out of the four key confounders

For peer review only - http://bmjopen.bmj.com/site/about/guidelines.xhtml

Primary outcome: risk factors for institutionalisation (Table 2)

Nine studies examined 25 different relationship quality factors as potential risk factors for institutionalisation. Follow-up ranged from 6 to 24 months. The majority of studies found no association between the risk factors investigated and the incidence of institutionalisation. Although some individual studies reported associations between relationship quality and institutionalisation, there were no consistent findings across risk factors, and the lack of appropriate adjustment for basic confounding factors makes interpreting the results very difficult.

For peer review only

For peer review only - http://bmjopen.bmj.com/site/about/guidelines.xhtml

Table 2: Associations between relationship factors and institutionalisation (9 studies)

Risk factor	Study	N	Follow-up	Results (95% CI; p-value)	Analysis adjusted for[1]
**Factors relating to the interaction between the person with dementia and their caregiver**					
Quality of relationship	Spruytte[46]	144	6-9m	OR 0.92 (p=0.02)	None
	Pruchno[35]	220	12m	OR 1.31 (p>0.05)	a,g,s,o
Marital happiness<br>At baseline	Wright[39]	29	24m	PoV 0.313 (p<0.01)	None
Before dementia onset				PoV 0.045 (p>0.05)	None
Emotional closeness	Fisher[44]	164	24m	OR 1.64 (1.09, 2.46; p=0.02)	d,o
Emotional distancing /boundary ambiguity	Fisher[44]	164	24m	OR 1.25 (0.85, 1.83; p=0.26)	d,o
	Wells[36]	93	12-18m	OR 1.3 (p>0.05)	a,d
Cohesion	Torossian[45]	197	24m	MD NR (p>0.05)	None
	Wright[39]	29	24m	PoV 0.472 (p<0.001)	None
Affection	Wright	29	24m	PoV 0.144 (p>0.05)	None
Warmth	Spruytte[46]	144	6-9m	NR (p<0.05)	None
Anxious-ambivalent attachment (carer)	Markiewicz[40]	108	24m	OR 1.27 (p=0.36)	None
Avoidant attachment (carer)	Markiewicz[40]	108	24m	OR 2.39 (p<0.001)	None
	Stevens[48]	215	24m	HR 1.011 (p=0.37)	None
Tension	Wright	29	24m	PoV 0.162 (p>0.05)	None
Criticism	Spruytte[46]	144	6-9m	NR (p>0.05)	None
**Factors mainly relating to the caregiver**					
Adaptability	Torossian[45]	197	24m	MD NR (p>0.05)	None
Approach coping	Stevens[48]	215	24m	HR 0.997 (p=0.77)	None
Directing relatives behaviour	Bannister[41]	116	12m	MD 0.2 (p=0.40)	None
Keeping relative busy	Bannister[41]	116	12m	MD 0.5 (p=0.02)	None
Learning about the illness	Bannister[41]	116	12m	MD 0.3 (p=0.42)	None
Prioritizing	Bannister[41]	116	12m	MD 0.1 (p=0.52)	None
Reducing expectations	Bannister[41]	116	12m	MD 0.4 (p=0.19)	None
Consistent larger sense of the illness	Bannister[41]	116	12m	MD 0.4 (p=0.35)	None
Positivity	Bannister[41]	116	12m	MD 0.5 (p=0.57)	None
	Wells[36]	93	12-18m	OR 1.03 (p>0.05)	a,d
Seeking social support	Wells[36]	93	12-18m	OR 1.91 (p<0.05)	a,d
Accepting responsibility	Wells[36]	93	12-18m	OR 0.28 (p<0.01)	a,d
Confrontational	Wells[36]	93	12-18m	OR 2.12 (p<0.05)	a,d
Negative feelings	Fisher[44]	164	24m	OR 1.47 (0.99, 2.19; p=0.05)	d,o

Legend: **OR** = odds ratio. OR>1 indicates increased risk, OR<1 indicates decreased risk. **HR** = hazard ratio, interpreted the same as OR. **PoV** = proportion of variance. This is a number between 0-1 indicating the proportion of variance in the outcome explained by the exposure. Higher numbers indicate greater explanatory power of the exposure. **Corr** = correlation. This is a number between -1 to +1 and indicates the degree to which the exposure and outcome vary together. Positive numbers indicate that exposure and outcome increase together, negative numbers indicate that the outcome increases as the exposure decreases. Larger numbers indicate stronger correlation. **MD** = mean difference in outcome. Large differences between groups suggests that the exposure might affect the outcome. **NR** = information not reported (where possible results and 95%CIs were calculated from raw data)

1. Pre-specified key confounders: **a** = age; **g** = gender; **s** = socioeconomic status; **d** = dementia severity.
o = study adjusted for other potential confounders.

For peer review only

Risk factors for challenging behaviour (BPSD) (Table 3)

Under the umbrella term of challenging behaviour four main types of outcome were evaluated: global challenging behaviour scores; psychotic symptoms; depression; and other BPSD outcomes. Eight studies examined nine different relationship quality factors as potential risk factors for these aspects of challenging behaviour. The length of follow-up ranged from 6 to 24 months.

There was a suggestion of an association between relationship factors and global challenging behaviour. All studies evaluating global challenging behaviour provided statistical evidence of an association (most p-values below 0.02). However, one study that reported two analyses did not report effect sizes.[37] For another the reported effect size was very small (mean difference of 0.23 in a scale of 1-144).[49] A larger effect size was seen for the association between expressed emotion and global challenging behaviours (mean difference of 1.9 in a scale of 0-8).[38] None of these analyses adjusted for our pre-specified confounding factors.

Most studies found no evidence of an association between relationship factors and either psychotic symptoms, depression or other BPSD outcomes. However, some of these were small studies that may have been underpowered to detect an association. One study adjusted for three out of four pre-specified potential confounders.[32] This was also one of the largest studies (n=171). It found no evidence for an association between couple mutuality and psychotic symptoms and a very weak, likely clinically negligible effect of this factor on depression (mean difference -0.43 points on a scale of 0-68).[32]

For peer review only - http://bmjopen.bmj.com/site/about/guidelines.xhtml

Table 3: Associations between relationship factors and Challenging behaviour (BPSD) (8 studies)

Risk factor	Study	N	Follow-up	Results (95% CI; p-value)	Analyses adjusted for[1]	Outcome assessment tool, scale range[2]
**Outcome: Global challenging behaviour (BPSD), or "problem behaviour" (4 studies)**						
Emotional distancing /boundary ambiguity	Caron[43]	60	12m	Corr 0.27 (p<0.001) (overall)	None	BEHAVE-AD, 0-75
Non-adaptive coping	deVugt[37]	69	6m	MD NR (p=0.007)	g,s,o	NPI, 1-144
		69	12m	MD NR (p=0.019)	g,s,o	
Avoidant attachment	Perren[49]	68	24m	MD 0.23 (p<0.05)	None	NPI, 1-144
Expressed emotion	Vitaliano[38]	77	15-18m	MD 1.9 (0.77, 3.04; p<0.001)	None	SCB, 0-8
**Outcome: Psychotic symptoms (3 studies)**						
Mutuality	Ball[32]	171	24m	MD -0.1 (-0.24, 0.04; p>0.05) (delusions)	a,g,d	NPI, 1-12
	Ball[32]	171	24m	MD -0.04 (-0.16, 0.08; p>0.05) (hallucinations)	a,g,d	
Emotional distancing /boundary ambiguity	Caron[43]	60	12m	Corr 0.30 (p<0.001) (paranoia)	None	BEHAVE-AD, 0-75
Non-adaptive coping	deVugt[37]	69	12m	MD NR (p>0.05) (psychosis)	g,s	NPI, 1-24
**Outcome: Depression (5 studies)**						
Mutuality	Ball[32]	171	24m	MD -0.43 (-0.80, 0.06; p<0.05)	a,g,d	Hamilton, 0-68
Quality of relationship	Burgener[20]	70	18m	Corr 0.22 (p>0.05)	None	Cornell, 0-38
Emotional distancing /boundary ambiguity	Caron[43]	60	12m	Corr 0.01 (p>0.05)	None	BEHAVE-AD, 0-75
Expressed emotion	Vitaliano[38]	77	15-18m	MD 2.00 (-0.31, 4.31; p>0.05)	None	Hamilton, 0-68
Cohesion quantity	Wright[42]	14	6m	Corr -0.302 (p>0.05)	None	Zung, 0-270
quality		14	6m	Corr -0.387 (p>0.05)	None	
Affection quantity	Wright[42]	14	6m	Corr -2.41 (p>0.05)	None	
quality		14	6m	Corr -0.038 (p>0.05)	None	
Tension quantity	Wright[42]	14	6m	Corr 0.533 (p<0.01)	None	
quality		14	6m	Corr -0.288 (p>0.05)	None	
**Outcome: Other**						
Non-adaptive coping	deVugt[37]	69	12m	MD NR (p=0.512) (apathy)	g,s,o	NPI, 1-48
Non-adaptive coping	deVugt[37]	69	12m	MD NR (p=0.005) (hyperactivity)	g,s,o	NPI, 1-60
Mutuality	Morgan[33]	171	24m	MD -0.42 (-0.64, 0.20; p<0.001) (aggression)	None	CMAI, 0-156
Emotional distancing /boundary ambiguity	Caron[43]	60	6m	Corr 0.15 (p>0.05) (anxiety)	None	BEHAVE-AD, 0-75

Legend: **OR** = odds ratio. OR>1 indicates increased risk, OR<1 indicates decreased risk. **HR** = hazard ratio, interpreted the same as OR. **PoV** = proportion of variance. This is a number between 0-1 indicating the proportion of variance in the outcome explained by the exposure. Higher numbers indicate greater explanatory power of the exposure. **Corr** = correlation. This is a number between -1 to +1 and indicates the degree to which the exposure and outcome vary together. Positive numbers indicate that exposure and outcome increase together, negative numbers indicate that the outcome increases as the exposure decreases. Larger numbers indicate stronger correlation. **MD** = mean difference in outcome. Large differences between groups suggests that the exposure might affect the outcome. **NR** = information not reported (where possible results and 95%CIs were calculated from raw data)

1. Pre-specified key confounders: **a** = age; **g** = gender; **s** = socioeconomic status; **d** = dementia severity.
o = study adjusted for other potential confounders.

2. NPI = Neuropsychiatric Inventory; CMAI = Cohen-Mansfield Agitation Inventory; Cornell = Cornell Scale for Depression in Dementia; SCB = Screen for Caregiver Burden (selection of items); Zung = Short Zung Interviewer Assisted Depression Rating Scale;

For peer review only - http://bmjopen.bmj.com/site/about/guidelines.xhtml

Risk factors for hospitalisation, quality of life, and death (Table 4)

The outcomes of hospitalisation,[34] quality of life[50] and death[47] were each examined in only one study. None of the analyses for adjusted for any of the pre-specified confounding factors. The small number of poor quality studies mean it is not possible to draw conclusions regarding the association of relationship factors with these outcomes.

Table 4: Associations between relationship factors and hospitalisation; quality of life; and death

Risk factor	Study	N	Follow-up	Results (95% CI; p-value)	Analyses adjusted for[1]
**Hospitalisation (1 study)**					
Relationship strain	Godwin[34]	296	12m	OR 1.03 (0.92, 1.14; p=0.637)	None
**Quality of life (1 study)**					
Quality of relationship (patient view)	Clare[50]	51	20m	MD 0.31 (p=0.06)	o
Quality of relationship (carer view)	Clare[50]	51	20m	MD -0.13 (p=0.89)	o
**Death (1 study)**					
Instrumental coping	McClendon[47]	141	5-9 years	HR 0.99 (p=0.915)	None
Acceptance coping	McClendon[47]	141	5-9 years	HR 1.09 (p=0.644)	None
Wishful thinking	McClendon[47]	141	5-9 years	HR 1.41 (p=0.019)	None

Legend: OR = odds ratio. OR>1 indicates increased risk, OR<1 indicates decreased risk. HR = hazard ratio, interpreted the same as OR. MD = mean difference in outcome. Large differences between groups suggests that the exposure might affect the outcome. NR = information not reported (where possible results and 95%CIs were calculated from raw data)
1. Pre-specified key confounders: a = age; g = gender; s = socioeconomic status; d = dementia severity.
o = study adjusted for other factors not expected to be key confounders.

For peer review only

For peer review only - http://bmjopen.bmj.com/site/about/guidelines.xhtml

DISCUSSION

This systematic review assessed the evidence on the role of relationship factors on outcomes in dementia. Although it is plausible that relationship factors could increase the risk of institutionalisation, challenging behaviour and other outcomes, there is currently no robust evidence to establish which, or to what extent, elements of the caring relationship affect specific outcomes.

The majority of studies found no association between specific risk factors and the outcomes of interest. However this is not necessarily evidence of a lack of association, as some of the studies will have been underpowered to detect differences, and most were at risk of confounding as they failed to adjust for even the most basic confounding factors. There was a suggestion of an association between factors related to the emotional withdrawal of the caregiver and subsequent increased risk of challenging behaviour in the person with dementia. All studies in this category found statistical evidence of an association. However, the methodological quality of these studies was poor. For example many did not report effect sizes while in others the effect sizes were small, suggesting that associations may not be clinically important. There was also a potential for confounding in studies that did report effect sizes.

Amongst the strengths of this review were that we conducted a thorough, sensitive search which will have minimised the chance of missing relevant studies. By limiting inclusion to cohort studies only, we avoided interpretive difficulties from recall bias (systematic differences in how people remember risk factors) and reverse causation (when a purported risk factor is in fact a result of the outcome). The double-screening of each record by two reviewers working independently also minimised the possibility of errors and selection bias in the identification of eligible reports. All data extracted was checked in full by a second reviewer working independently.

For peer review only - http://bmjopen.bmj.com/site/about/guidelines.xhtml

A limitation of our review is that we were not able to assess for publication bias or selective reporting of results. It is known that studies with null findings are less likely to be published, and authors can selectively report their 'more interesting' findings.[51] This means it is possible there are other relevant results that have not been reported, and so would not be available to a review. We attempted to minimise the risk of publication bias by including a grey literature search.

Our ability to answer the research question was limited due to poor reporting, few studies assessing similar risk factors, and potential for bias in included studies. We considered the appropriateness of using meta-analysis to produce summary estimates. However, many studies did not report full details of effect sizes and confidence intervals that are required for meta-analysis. Additionally most risk factor-outcome relationships were only reported in one or two studies (with conflicting results in the latter cases) which is insufficient for meta-analyses. It would not have been meaningful or appropriate to combine different risk factors or outcomes in pooled analysis. We assessed the potential for bias in included studies and took these assessments into consideration when reaching conclusions, thus avoiding over-interpretation of findings from studies with potentially serious problems.

Very few studies adjusted their analyses for key pre-specified confounding factors (age, gender, socioeconomic status, and dementia severity). Two thirds of included analyses did not adjust for any confounding factors. This is an important limitation as it means any findings could be explained by differences in these factors across the exposure groups – differences that are very likely in a non-randomised study where participants self-select into exposure groups. A small number of studies adjusted for other factors, but these were factors such as quality of life and emotional withdrawal that may well be on the causal pathway between the relationship factor studied and the outcome (so not a true confounder). Reporting of results was poor with many studies only reporting p-values, or just that an association was 'statistically significant' / 'not significant' with no effect sizes or

For peer review only - http://bmjopen.bmj.com/site/about/guidelines.xhtml

confidence intervals. This is another important limitation as in these cases we have no information about the probable magnitude of effect, and it is impossible to interpret the potential clinical implications of any statistical difference. Poor reporting of methods and results made it difficult to assess risk of bias. Very few studies evaluated the same risk factors, so there is also very little evidence on any individual factor. Duration of follow-up (typically 6-24 months) may not have been sufficient to detect outcomes in many studies. As dementia is typically a slowly progressing disease this is unlikely have been sufficient for long-term outcomes such as institutionalisation.

The current evidence does not provide a basis by which GPs or other health professionals could reliably identify people at risk of poor outcome on the basis of relationship factors. However, the lack of robust evidence about the role of relationship factors does not imply that personal relationships are not important factors in dementia outcomes; many professionals working with families consider these to be important.[52,53] One plausible mechanism is that factors such as emotional withdrawal of the caregiver might prompt ‘challenging behaviour’ in the person with dementia as an attempt to elicit an emotional connection.[49] For instance, care-giver coping strategies such as constantly ‘correcting’ the person with dementia, rather than accepting their cognitive challenges, could be construed as potentially undermining the individual’s personhood or self-esteem, both of which are seen as key elements in good dementia care. In this way, any ‘challenging behaviour’ might be construed as behavioural expressions of underlying frustration or distress. It is also possible that challenging behaviours are themselves on the causal pathway between relationship factors and institutionalisation – the latter plausibly becoming more likely as the caregiver becomes less able to cope with challenging behaviours. If this is the case then longer follow-up may be necessary to detect associations between relationship factors and institutionalisation.

For peer review only - http://bmjopen.bmj.com/site/about/guidelines.xhtml

Lack of evidence on what relationship factors predict outcomes in people with dementia should not preclude further evaluation of psychosocial interventions targeting people with dementia and their carers. Such studies could use experimental designs to identify which interventions work and in which settings. It may also be useful to look to qualitative research, exploring the views of people with dementia and their family carers on what they consider important for continued living at home, and the challenges in the relationship that they face.

CONCLUSIONS

There is currently no strong or consistent evidence on the effects of relationship factors on institutionalisation, hospitalisation, death or quality of life for people with dementia. There was a suggestion of an association between relationship factors and challenging behaviour, although the evidence for this was weak. As the current focus of dementia care is 'person-centred', which prioritises interpersonal relationships, this lack of evidence about the role of relationships in dementia outcomes is striking. To improve our ability to support those with dementia and their families this evidence gap needs to be addressed.

For peer review only

FIGURES

Figure 1: PRISMA flow diagram

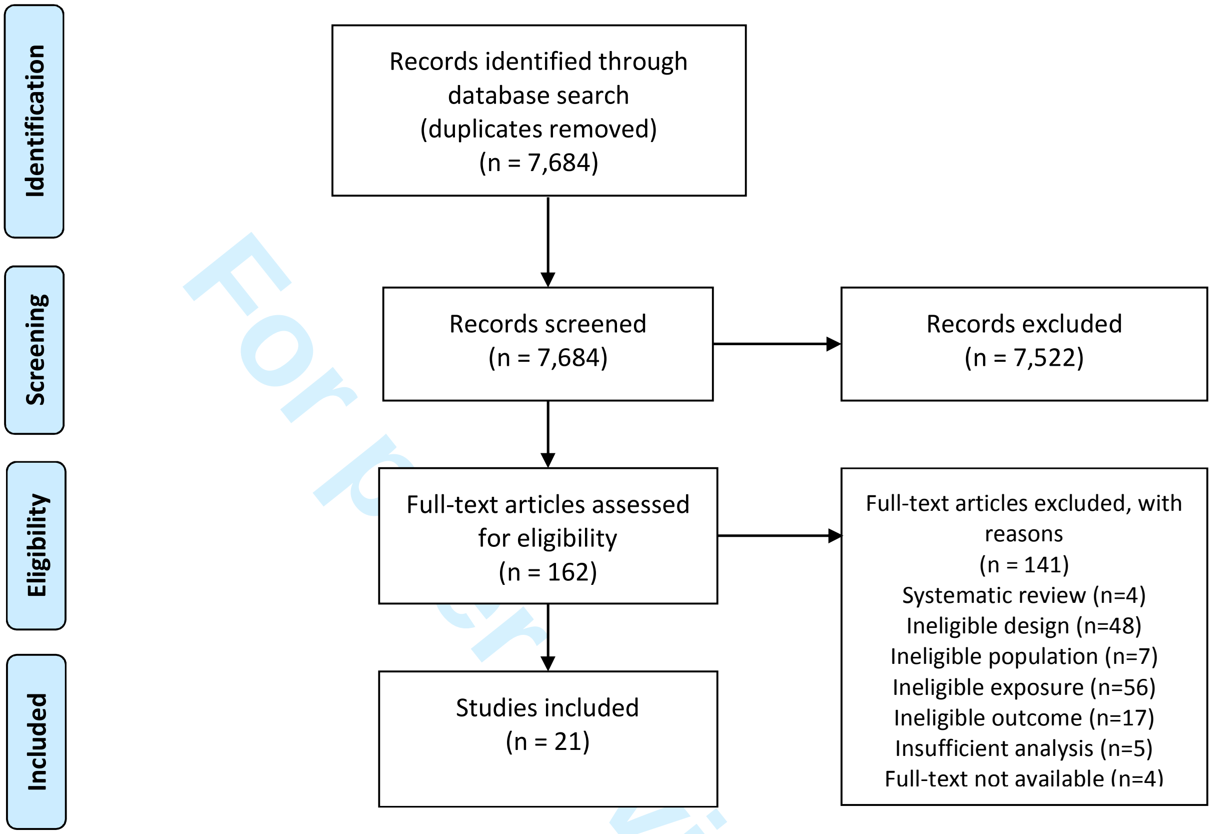

For peer review only - http://bmjopen.bmj.com/site/about/guidelines.xhtml

APPENDICES / SUPPLEMENTARY FILES

Appendix A: Search strategy

Supplementary file: Systematic review protocol

Funding statements and acknowledgements

This research is supported by the National Institute for Health Research (NIHR) Collaboration for Leadership in Applied Health Research and Care West at University Hospitals Bristol NHS Foundation Trust. The views expressed are those of the author(s) and not necessarily those of the NHS, the NIHR or the Department of Health. Authors thank Becky Beynon for support screening papers.

Competing interests

The authors declare that they have no competing interests.

Authors' contributions

HE was the lead researcher, contributed to the study design, carried out screening, data extraction, data analysis, and prepared the manuscript. SI and VL contributed to the screening and data extraction. SI also contributed to the risk of bias assessment. PW and JS led the study design, supervised the review and contributed to drafting the manuscript. AR developed the search strategy and conducted the searches. RC and SC conceived the original study idea, and contributed to the study design. RC provided clinical advice throughout the review. All authors read and approved the final manuscript.

For peer review only

References

1. Fineberg NA, Haddad PM, Carpenter L, et al. The size, burden and cost of disorders of the brain in the UK. *Journal of Psychopharmacology.* 2013.
2. Luengo-Fernandez R, Leal J, Gray AM. UK research expenditure on dementia, heart disease, stroke and cancer: are levels of spending related to disease burden? *Eur J Neurol.* 2012;19(1):149-154.
3. WHO. Dementia (Fact sheet no. 362). *http://wwwwhoint/mediacentre/factsheets/fs362/en/*. 2015.
4. Prince M, Bryce R, Albanese E, Wimo A, Ribeiro W, Ferri CP. The global prevalence of dementia: A systematic review and metaanalysis. *Alzheimer's & Dementia.* 2013;9(1):63-75.e62.
5. Prince M, Knapp, M, Guerchet, M, McCrone, P, Prina, M, Comas-Herrera, A, Wittenberg, R, Adelaja, B, Hu, B, King, D, Rehill, A and Salimkumar, D (Alzheimer's Society). Dementia UK Update (Second Edition). In: Society As, ed2014.
6. International AsD. *World Alzheimer Report 2014: Dementia and Risk Reduction. An analysis of protective and modifiable factors.* September 2014 2014.
7. Byrne-Davis LMT, Bennett PD, Wilcock GK. How are Quality of Life Ratings Made? Toward a Model of Quality of Life in People with Dementia. *Qual Life Res.* 2006;15(5):855-865.
8. Donnelly L. Care Quality Commission finds 'appalling' failings in elderly care. *Telegraph.* Vol http://www.telegraph.co.uk/news/health/elder/11167179/Care-Quality-Commission-finds-appalling-failings-in-elderly-care.htmlOctober 2014.
9. Sawer P, Ross, T. Dozens of care homes failing the most vulnerable. *Telegraph.* Vol http://www.telegraph.co.uk/news/health/elder/11292305/Dozens-of-care-homes-failing-the-most-vulnerable.htmlDecember 2014.
10. YouGov. Care home concerns. *https://yougovcouk/news/2013/03/06/care-home-concerns/*. March 2013.
11. Health Do. *Prime Minister's challenge on dementia 2020.* 21 February 2015.
12. Tschanz JT, Piercy K, Corcoran CD, et al. Caregiver Coping Strategies Predict Cognitive and Functional Decline in Dementia: The Cache County Dementia Progression Study. *The American Journal of Geriatric Psychiatry.* 2013;21(1):57-66.
13. García-Alberca JM, Cruz B, Lara JP, et al. The experience of caregiving: the influence of coping strategies on behavioral and psychological symptoms in patients with Alzheimer's disease. *Aging & Mental Health.* 2013;17(5):615-622.
14. Luppa M, Riedel-Heller SG, Stein J, et al. Predictors of Institutionalisation in Incident Dementia – Results of the German Study on Ageing, Cognition and Dementia in Primary Care Patients (AgeCoDe Study). *Dement Geriatr Cogn Disord.* 2012;33(4):282-288.
15. Fauth E, Hess K, Piercy K, et al. Caregivers' relationship closeness with the person with dementia predicts both positive and negative outcomes for caregivers' physical health and psychological well-being. *Aging & Mental Health.* 2012;16(6):699-711.
16. Quinn C, Clare L, Woods B. The impact of the quality of relationship on the experiences and wellbeing of caregivers of people with dementia: A systematic review. *Aging & Mental Health.* 2009;13(2):143-154.
17. Norton MC, Piercy KW, Rabins PV, et al. Caregiver–Recipient Closeness and Symptom Progression in Alzheimer Disease. The Cache County Dementia Progression Study. *The Journals of Gerontology Series B: Psychological Sciences and Social Sciences.* 2009;64B(5):560-568.
18. Spitznagel MB, Tremont G, Davis JD, Foster SM. Psychosocial Predictors of Dementia Caregiver Desire to Institutionalize: Caregiver, Care Recipient, and Family Relationship Factors. *J Geriatr Psychiatry Neurol.* 2006;19(1):16-20.

For peer review only - http://bmjopen.bmj.com/site/about/guidelines.xhtml
For peer review only

19. Banerjee S, Murray J, Foley B, Atkins L, Schneider J, Mann A. Predictors of institutionalisation in people with dementia. *J Neurol Neurosurg Psychiatry.* 2003;74(9):1315-1316.
20. Burgener S, Twigg P. Relationships Among Caregiver Factors and Quality of Life in Care Recipients with Irreversible Dementia. *Alzheimer Dis Assoc Disord.* 2002;16(2):88-102.
21. Bergmann K, Manchee V, Woods RT. Effect of family relationships on psychogeriatric patients. *J R Soc Med.* 1984;77(10):840-844.
22. Benbow SM, Sharman V. Review of family therapy and dementia: twenty-five years on. *Int Psychogeriatr.* 2014;26(Special Issue 12):2037-2050.
23. Cheston R, Ivanecka A. Individual and group psychotherapy with people diagnosed with dementia: a systematic review of the literature. *Int J Geriatr Psychiatry.* 2016:n/a-n/a.
24. Edwards HB, Savović J, Whiting P, et al. Quality of relationships as predictors of outcomes in people with dementia: a systematic review protocol. *BMJ Open.* 2016;6(4).
25. Hazan C, Shaver P. Romantic love conceptualized as an attachment process. *J Pers Soc Psychol.* 1987;52(3):511-524.
26. Hazan C, Shaver PR. Attachment as an Organizational Framework for Research on Close Relationships. *Psychol Inq.* 1994;5(1):1-22.
27. Kuipers L, Bebbington P. Expressed emotion research in schizophrenia: theoretical and clinical implications. *Psychol Med.* 1988;18(4):893-909.
28. Raune D, Kuipers E, Bebbington PE. Expressed emotion at first-episode psychosis: investigating a carer appraisal model. *The British Journal of Psychiatry.* 2004;184(4):321-326.
29. GA Wells BS, D O'Connell, J Peterson, V Welch, M Losos, P Tugwell. The Newcastle-Ottawa Scale (NOS) for assessing the quality of nonrandomised studies in meta-analyses http://www.ohri.ca/programs/clinical_epidemiology/oxford.asp. Accessed September 2015.
30. Jüni P, Witschi A, Bloch R, Egger M. The hazards of scoring the quality of clinical trials for meta-analysis. *JAMA.* 1999;282(11):1054-1060.
31. Kunik ME, Snow AL, Davila JA, et al. Causes of aggressive behavior in patients with dementia. *J Clin Psychiatry.* 2010;71(9):1145-1152.
32. Ball V, Snow AL, Steele AB, et al. Quality of Relationships as a Predictor of Psychosocial Functioning in Patients With Dementia. *J Geriatr Psychiatry Neurol.* 2010;23(2):109-114.
33. Morgan RO, Sail KR, Snow AL, Davila JA, Fouladi NN, Kunik ME. Modeling Causes of Aggressive Behavior in Patients With Dementia. *The Gerontologist.* 2013;53(5):738-747.
34. Godwin KM, Morgan RO, Walder A, et al. Predictors of Inpatient Utilization among Veterans with Dementia. *Current Gerontology and Geriatrics Research.* 2014;2014:10.
35. Pruchno RA, Michaels JE, Potashnik SL. Predictors of Institutionalization Among Alzheimer Disease Victims With Caregiving Spouses. *J Gerontol.* 1990;45(6):S259-S266.
36. Wells Y, Over R. Institutional Placement of a Dementing Spouse: The Influence of Appraisal, Coping Strategies, and Social Support. *Aust J Psychol.* 1998;50(2):100-105.
37. de Vugt ME, Stevens F, Aalten P, et al. Do caregiver management strategies influence patient behaviour in dementia? *Int J Geriatr Psychiatry.* 2004;19(1):85-92.
38. Vitaliano PP, Young HM, Russo J, Romano J, Magana-Amato A. Does Expressed Emotion in Spouses Predict Subsequent Problems Among Care Recipients With Alzheimer's Disease? *J Gerontol.* 1993;48(4):P202-P209.
39. Wright LK. Alzheimer's disease afflicted spouses who remain at home: can human dialectics explain the findings? *Soc Sci Med.* 1994;38(8):1037-1046.
40. Markiewicz D, Reis M, Gold DP. An exploration of attachment styles and personality traits in caregiving for dementia patients. *Int J Aging Hum Dev.* 1997;45(2):111-132.
41. Bannister C, Ballard C, Lana M, Fairbairn A, Wilcock G. Placement of dementia sufferers in residential and nursing home care. *Age Ageing.* 1998;27(2):189-193.
42. Wright LK, Hickey JV, Buckwalter KC, Kelechi T, Hendrix S. Spousal interactions in Alzheimer's disease and stroke caregiving: Relationship to care recipients' functional abilities and physical and emotional health. *J Am Psychiatr Nurses Assoc.* 1998;4(6):169-181.

For peer review only

For peer review only - http://bmjopen.bmj.com/site/about/guidelines.xhtml

43. Caron W, Boss P, Mortimer J. Family boundary ambiguity predicts Alzheimer's outcomes. *Psychiatry.* 1999;62(4):347-356.
44. Fisher L, Lieberman MA. A Longitudinal Study of Predictors of Nursing Home Placement for Patients With Dementia: The Contribution of Family Characteristics. *The Gerontologist.* 1999;39(6):677-686.
45. Torossian CL, Ruffins S. Relationship Between Family Dynamics of Caregivers, Depression, and the Likelihood of Institutionalization of Alzheimer's Patients. *Journal of Contemporary Psychotherapy.* 1999;29(2):127-142.
46. Spruytte N, Van Audenhove C, Lammertyn F. Predictors of institutionalization of cognitively-impaired elderly cared for by their relatives. *Int J Geriatr Psychiatry.* 2001;16(12):1119-1128.
47. McClendon MJ, Smyth KA, Neundorfer MM. Survival of Persons With Alzheimer's Disease: Caregiver Coping Matters. *The Gerontologist.* 2004;44(4):508-519.
48. Stevens A, Owen J, Roth D, Clay O, Bartolucci A, Haley W. Predictors of time to nursing home placement in White and African American individuals with dementia. *J Aging Health.* 2004;16(3):375-397.
49. Perren S, Schmid R, Herrmann S, Wettstein A. The impact of attachment on dementia-related problem behavior and spousal caregivers' well-being. *Attachment & Human Development.* 2007;9(2):163-178.
50. Clare L, Woods RT, Nelis SM, et al. Trajectories of quality of life in early-stage dementia: individual variations and predictors of change. *Int J Geriatr Psychiatry.* 2014;29(6):616-623.
51. Dwan K, Gamble C, Williamson PR, Kirkham JJ, for the Reporting Bias G, the Reporting Bias G. Systematic Review of the Empirical Evidence of Study Publication Bias and Outcome Reporting Bias — An Updated Review. *PLoS One.* 2013;8(7):e66844.
52. T K. *Dementia Reconsidered: The Person Comes First* Buckingham: Open University Press; 1997.
53. Cheston R BM. *Understanding Dementia: The Man with the Worried Eyes.* London: Jessica Kingsley Press; 1999.

For peer review only

For peer review only - http://bmjopen.bmj.com/site/about/guidelines.xhtml

SEARCH STRATEGY

Systematic Review: Individual and family relationships as predictors of outcomes in people affected by dementia: a systematic review

Databases: Embase, Medline, Psychinfo, Web of Science, Cochrane.
The search strategy for each database is detailed below.

EMBASE

Database: Embase <1974 to 2015 May 11>
Search Strategy:
----
1 *dementia/ or *alzheimer disease/ or *cadasil/ or *diffuse lewy body disease/ or *diffuse neurofibrillary tangles with calcification/ or exp *frontotemporal dementia/ or *hiv associated dementia/ or *multiinfarct dementia/ or *pick presenile dementia/ or *presenile dementia/ or exp *senile dementia/ (127188)
2 alzheimer$.ti,ab. (127578)
3 (dementia or demented).ti. (44409)
4 ((dementia or demented) adj4 (senile or senility or elder$ or person$ or subject$ or adult$ or patient$ or people)).ab. (32436)
5 or/1-4 (183931)
6 *caregiver/ or *caregiver burden/ or *caregiver support/ or caregiver strain index/ (15454)
7 *family relation/ or *family conflict/ (2945)
8 *child parent relation/ (18108)
9 emotional intelligence/ (1316)
10 emotional attachment/ (4306)
11 family coping/ or family functioning/ or family interaction/ (3562)
12 ((carer$ or caregiver$ or care-giver$ or pre-caregiv$ or caregiving or care-giving) adj4 (quality of life or Qol or conflict$ or morbidity or capabilit$ or depression or depressive or anxiety or closeness or motivat$ or resilien$ or communicat$ or support or counsel$ or informal or factor$ or distress$ or relationship$ or stress or burden$ or support$ or interaction$ or commitment or wellbeing or well-being or style$ or dysfunctional or dependen$ or independen$ or intimat$ or affection or affectionate or tension$ or pressure$ or disagreement$ or isolat$ or loving)).ti,ab. (20332)
13 ((marital or spous$ or couple$ or daughter$ or son or son$2 or partner$ or husband$ or wife$ or wives or familial or families) adj4 (quality of life or Qol or morbidity or counsel$ or support or depression or depressive or anxiety or closeness or motivat$ or resilien$ or capabilit$ or communicat$ or informal or factor$ or distress$ or relationship$ or stress or burden$ or support$ or interaction$ or commitment or wellbeing or well-being or conflict$ or style$ or dysfunctional or dependen$ or independen$ or intimat$ or affection or affectionate or tension$ or pressure$ or disagreement$ or isolat$ or loving)).ti,ab. (74168)
14 (Relationship$ adj3 (loving or intimate or intimacy or affection or affectionate or quality or close$ or dysfunctional or dependen$ or predictor$)).ti,ab. (36940)
15 new york university caregiver intervention.ti,ab. (7)
16 *marriage/ or *spouse/ (15122)
17 "sense of coherence"/ (641)
18 (expressed adj2 emotion).ti,ab. (1222)
19 ((role captivity or self efficacy or sense of competence or sense of coherence) and (carer$ or caregiver$ or care-giver$ or pre-caregiv$ or caregiving or care-giving or marital or spous$ or couple$ or daughter$ or son or son$2 or partner$ or husband$ or wife$ or wives or familial or families)).ti,ab. (3054)
20 (attachment adj2 (style$ or avoidant or insecure or secure or ambivalent)).ti,ab. (2432)

21 exp *adaptive behavior/ or *coping behavior/ (30848)
22 (coping adj2 (acceptance-based or solution-based or positive or abilit$ or strateg$ or style$ or mechanism$ or strain or stress)).ti,ab. (21085)
23 *social isolation/ or *loneliness/ (6328)
24 (social adj2 isolation).ti,ab. (5882)
25 (emotional adj2 (strain or impact or health or support)).ti,ab. (10379)
26 *social support/ or *respite care/ (15162)
27 ((loneliness or social support or time to listen or befriend$ or lines of communication) and (carer$ or caregiver$ or care-giver$ or pre-caregiv$ or caregiving or care-giving or marital or spous$ or couple$ or daughter$ or son or son$2 or partner$ or husband$ or wife$ or wives or familial or families)).ti,ab. (8387)
28 (respite adj2 (break$ or care)).ti,ab. (749)
29 or/6-28 (243201)
30 5 and 29 (7756)
31 letter.pt. or letter/ (889069)
32 note.pt. (598597)
33 editorial.pt. (476664)
34 case report/ or case study/ (2029980)
35 (letter or comment$).ti. (157341)
36 or/31-35 (3836762)
37 exp clinical study/ or meta analysis/ or "meta analysis (topic)"/ or "systematic review"/ or "systematic review (topic)"/ or cross-sectional study/ or crossover procedure/ or (systematic review or meta-analys$ or study or trial or cross-over or crossover or cross-sectional or case-control or cohort$ or blind$ or random$).mp. (14600734)
38 36 not 37 (1524603)
39 exp animal/ not human/ (4494915)
40 nonhuman/ (4504835)
41 exp experimental animal/ (457411)
42 exp animal experiment/ (1848987)
43 exp animal model/ (829666)
44 exp rodent/ (3024267)
45 (rat or rats or mouse or mice).ti. (1317756)
46 or/38-45 (8447146)
47 30 not 46 (7357)
48 meta analysis/ or "meta analysis (topic)"/ or "systematic review"/ or "systematic review (topic)"/ (166636)
49 (meta analy$ or metaanaly$ or metanaly$ or meta regression).ti,ab. (99458)
50 ((systematic$ or evidence$) adj2 (review$ or overview$)).ti,ab. (108142)
51 (reference list$ or bibliograph$ or hand search$ or manual search$ or relevant journals).ab. (33172)
52 (search strategy or search criteria or systematic search or study selection or data extraction).ab. (34138)
53 (search$ adj4 literature).ab. (40229)
54 (medline or pubmed or cochrane or embase or psychlit or psyclit or psychinfo or cinahl or science citation index or bids or cancerlit).ab. (123876)
55 cochrane.jw. (12418)
56 ((multiple treatment$ or indirect or mixed) adj2 comparison).ti,ab. (1799)
57 or/48-56 (317594)
58 randomized controlled trial/ or "randomized controlled trial (topic)"/ (442912)
59 crossover procedure/ (42753)
60 "double blind procedure"/ (122609)
61 "single-blind procedure"/ (20152)
62 (randomi$ or randomly).ti,ab. (795220)
63 trial.ti. (181805)
64 (cross-over$ or crossover$ or cross over$).ti,ab. (75959)

For peer review only

65 ((doubl$ or singl$) adj blind$).ti,ab. (171033)
66 or/58-65 (1067625)
67 Clinical study/ (110797)
68 exp case control study/ (99829)
69 family study/ (10770)
70 longitudinal study/ (76925)
71 retrospective study/ (399400)
72 prospective study/ (289051)
73 cross-sectional study/ (143173)
74 cohort analysis/ (200367)
75 follow up/ (910549)
76 cohort$.ti,ab. (471173)
77 75 and 76 (98758)
78 (cross-sectional or case-control or interrupted time series).ti,ab. (337093)
79 (cohort adj (study or studies or analys$)).ti,ab. (138586)
80 ((follow up or followup or observational or non randomi#ed or uncontrolledl or "before and after") adj2 (study or studies)).ti,ab. (156520)
81 ((longitudinal$ or retrospectiv$ or prospectiv$) adj6 (study or studies or review or analys$ or cohort$)).ti,ab. (884195)
82 or/67-74,77-81 (1856248)
83 57 or 66 or 82 (2929368)
84 prognosis/ (453898)
85 disease course/ (317345)
86 treatment outcome/ or outcome assessment/ (929562)
87 disease severity assessment/ or apache/ (9250)
88 kaplan meier method/ (38059)
89 patient acuity/ (279)
90 "severity of illness index"/ (8484)
91 statistical model/ (115081)
92 exp regression analysis/ or statistical analysis/ (469010)
93 proportional hazards model/ or multivariate analysis/ (156362)
94 (likelihood or probabilit$ or predict$ or prognos$ or progress$ or logistic regression).ti,ab. (2886071)
95 ((multivariate or regression) adj2 analys$).ti,ab. (389260)
96 ((cox or linear or hazard$ or mixed or proportional or logistic) adj3 model$).ti,ab. (182048)
97 institutionalization/ or institutionali$.ti,ab. (18870)
98 or/84-97 (4319535)
99 83 or 98 (6105919)
100 47 and 99 (3985)

MEDLINE

Database: Medline In-process - Current week, Medline 1950 to present
Search Strategy:
----
1 dementia/ or aids dementia complex/ or alzheimer disease/ or exp aphasia, primary progressive/ or exp dementia, vascular/ or diffuse neurofibrillary tangles with calcification/ or exp frontotemporal lobar degeneration/ or lewy body disease/ (111181)
2 alzheimer$.ti,ab. (98468)
3 (dementia or demented).ti. (33717)
4 ((dementia or demented) adj4 (person$ or senile or senility or elder$ or subject$ or adult$ or patient$ or people)).ab. (23494)
5 or/1-4 (151599)

For peer review only

6 *Caregivers/ or caregivers/px (18460)
7 family relations/ or family conflict/ (9371)
8 intergenerational relations/ or parent-child relations/ (31073)
9 emotional intelligence/ (1004)
10 *interpersonal relations/ (23404)
11 family/px or spouses/ or marriage/px (26847)
12 ((carer$ or caregiver$ or care-giver$ or pre-caregiv$ or caregiving or care-giving) adj4 (quality of life or Qol or conflict$ or morbidity or capabilit$ or depression or depressive or anxiety or closeness or motivat$ or resilien$ or communicat$ or support or counsel$ or informal or factor$ or distress$ or relationship$ or stress or burden$ or support$ or interaction$ or commitment or wellbeing or well-being or style$ or dysfunctional or dependen$ or independen$ or intimat$ or affection or affectionate or tension$ or pressure$ or disagreement$ or isolat$ or loving)).ti,ab. (14943)
13 ((marital or spous$ or couple$ or daughter$ or son or son$2 or partner$ or husband$ or wife$ or wives or familial or families) adj4 (quality of life or Qol or morbidity or counsel$ or support or depression or depressive or anxiety or closeness or motivat$ or resilien$ or capabilit$ or communicat$ or informal or factor$ or distress$ or relationship$ or stress or burden$ or support$ or interaction$ or commitment or wellbeing or well-being or conflict$ or style$ or dysfunctional or dependen$ or independen$ or intimat$ or affection or affectionate or tension$ or pressure$ or disagreement$ or isolat$ or loving)).ti,ab. (60894)
14 (Relationship$ adj3 (loving or intimate or intimacy or affection or affectionate or quality or close$ or dysfunctional or dependen$ or predictor$)).ti,ab. (30669)
15 new york university caregiver intervention.ti,ab. (5)
16 Expressed Emotion/ (1466)
17 sense of coherence/ (315)
18 (expressed adj2 emotion).ti,ab. (997)
19 ((role captivity or self efficacy or sense of competence or sense of coherence) and (carer$ or caregiver$ or care-giver$ or pre-caregiv$ or caregiving or care-giving or marital or spous$ or couple$ or daughter$ or son or son$2 or partner$ or husband$ or wife$ or wives or familial or families)).ti,ab. (2199)
20 (attachment adj2 (style$ or avoidant or insecure or secure or ambivalent)).ti,ab. (1866)
21 *adaptation, psychological/ (33853)
22 (coping adj2 (acceptance-based or solution-based or positive or abilit$ or strateg$ or style$ or mechanism$ or strain or stress)).ti,ab. (16036)
23 social isolation/ or loneliness/ (13056)
24 (social adj2 isolation).ti,ab. (4417)
25 (emotional adj2 (strain or impact or health or support)).ti,ab. (7768)
26 *social support/ or respite care/ (19752)
27 ((loneliness or social support or time to listen or befriend$ or lines of communication) and (carer$ or caregiver$ or care-giver$ or pre-caregiv$ or caregiving or care-giving or marital or spous$ or couple$ or daughter$ or son or son$2 or partner$ or husband$ or wife$ or wives or familial or families)).ti,ab. (6261)
28 (respite adj2 (break$ or care)).ti,ab. (591)
29 or/6-28 (255631)
30 5 and 29 (7435)
31 letter/ (878639)
32 editorial/ (377659)
33 news/ (168499)
34 exp historical article/ (333775)
35 Anecdotes as topic/ (4623)
36 comment/ (626697)
37 case report/ (1731222)
38 (letter or comment).ti. (66298)
39 or/31-38 (3462608)

For peer review only

40 exp clinical trial/ or exp clinical trials as Topic/ or meta-analysis/ or meta-analysis as topic/ or cross-over studies/ or exp epidemiologic studies/ or (systematic review or meta-analys$ or study or trial or cross over or crossover or cross sectional or case control or cohort$ or blind$ or random$).mp. (7668336)
41 39 not 40 (3093302)
42 animals/ not humans/ (3943260)
43 exp Animals, Laboratory/ (745617)
44 exp Animal Experimentation/ (6628)
45 exp Models, Animal/ (436913)
46 exp rodentia/ (2736988)
47 (rat or rats or mouse or mice).ti. (1142420)
48 or/41-47 (7676181)
49 30 not 48 (6825)
50 meta-analysis/ (55637)
51 meta-analysis as topic/ (14222)
52 (meta analy$ or metaanaly$ or metanaly$ or meta regression).ti,ab. (77420)
53 ((systematic$ or evidence$) adj2 (review$ or overview$)).ti,ab. (88839)
54 (reference list$ or bibliograph$ or hand search$ or manual search$ or relevant journals).ab. (28082)
55 (search strategy or search criteria or systematic search or study selection or data extraction).ab. (30051)
56 (search$ adj4 literature).ab. (32198)
57 (medline or pubmed or cochrane or embase or psychlit or psyclit or psychinfo or cinahl or science citation index or bids or cancerlit).ab. (100580)
58 cochrane.jw. (11498)
59 ((multiple treatment$ or indirect or mixed) adj2 comparison).ti,ab. (1034)
60 or/50-59 (227071)
61 randomized controlled trial.pt. or randomized controlled trial/ or Randomized Controlled Trials as Topic/ (486988)
62 controlled clinical trial.pt. (89427)
63 (randomi$ or randomly).ti,ab. (604805)
64 trial.ti. (138376)
65 clinical trials as topic.sh. (172816)
66 or/61-65 (1005304)
67 epidemiologic studies/ (6184)
68 cross-over studies/ (35964)
69 exp case control studies/ (713979)
70 exp cohort studies/ (1432164)
71 cross-sectional studies/ (193001)
72 "Controlled Before-After Studies"/ (35)
73 interrupted time series analysis/ (35)
74 case control.ti,ab. (85271)
75 (cohort adj (study or studies or analys$)).ti,ab. (104741)
76 ((follow up or followup or uncontrolled or non randomi#ed or observational or "before and after") adj2 (study or studies)).ti,ab. (116321)
77 ((longitudinal$ or retrospectiv$ or prospectiv$) adj6 (study or studies or review or analys$ or cohort$)).ti,ab. (627937)
78 cross sectional.ti,ab. (192858)
79 interrupted time series.ti,ab. (1226)
80 (control$ adj4 (group$ or trial or study)).ti,ab. (593653)
81 (cross-over$ or crossover$ or cross over$).ti,ab. (65294)
82 or/67-81 (2506236)
83 prognosis/ or survival analysis/ or kaplan-meier estimate/ or treatment outcome/ or "Outcome Assessment (Health Care)"/ (1117694)
84 Disease Progression/ (110573)

For peer review only

85 health status indicators/ or apache/ or patient acuity/ or "severity of illness index"/ (201441)
86 (likelihood or probabilit$).ti,ab. (223333)
87 (predict$ or prognos$ or progress$).ti,ab. (2002491)
88 models, statistical/ or likelihood functions/ or linear models/ or logistic models/ or proportional hazards models/ or regression analysis/ or least-squares analysis/ (389820)
89 ((multivariate or regression) adj2 analys$).ti,ab. (287877)
90 logistic regression.ti,ab. (152336)
91 ((cox or linear or hazard$ or mixed or proportional or logistic) adj3 model$).ti,ab. (131889)
92 Institutionalization/ or Nursing Homes/ut or institutionali$.ti,ab. (15705)
93 or/83-92 (3413126)
94 60 or 66 or 82 or 93 (5399217)
95 49 and 94 (3874)

PSYCHINFO
Database: PsycINFO <1806 to May Week 1 2015>
Search Strategy:
----
1 dementia/ or aids dementia complex/ or dementia with lewy bodies/ or presenile dementia/ or semantic dementia/ or exp senile dementia/ or vascular dementia/ or alzheimer's disease/ or picks disease/ (56057)
2 alzheimer$.ti,ab. (42977)
3 (dementia or demented).ti. (22125)
4 ((dementia or demented) adj4 (elder$ or senile or senility or person$ or subject$ or adult$ or patient$ or people)).ti,ab. (18797)
5 or/1-4 (66161)
6 ((carer$ or caregiver$ or care-giver$ or pre-caregiv$ or caregiving or care-giving) adj4 (quality of life or Qol or conflict$ or morbidity or capabilit$ or depression or depressive or anxiety or closeness or motivat$ or resilien$ or communicat$ or support or counsel$ or informal or factor$ or distress$ or relationship$ or stress or burden$ or support$ or interaction$ or commitment or wellbeing or well-being or style$ or dysfunctional or dependen$ or independen$ or intimat$ or affection or affectionate or tension$ or pressure$ or disagreement$ or isolat$ or loving)).ti,ab. (15209)
7 ((marital or spous$ or couple$ or daughter$ or son or son$2 or partner$ or husband$ or wife$ or wives or familial or families) adj4 (quality of life or Qol or morbidity or counsel$ or support or depression or depressive or anxiety or closeness or motivat$ or resilien$ or capabilit$ or communicat$ or informal or factor$ or distress$ or relationship$ or stress or burden$ or support$ or interaction$ or commitment or wellbeing or well-being or conflict$ or style$ or dysfunctional or dependen$ or independen$ or intimat$ or affection or affectionate or tension$ or pressure$ or disagreement$ or isolat$ or loving)).ti,ab. (63974)
8 (Relationship$ adj3 (loving or intimate or intimacy or affection or affectionate or quality or close$ or dysfunctional or dependen$ or predictor$)).ti,ab. (25220)
9 new york university caregiver intervention.ti,ab. (3)
10 (expressed adj2 emotion).ti,ab. (1585)
11 ((role captivity or self efficacy or sense of competence or sense of coherence) and (carer$ or caregiver$ or care-giver$ or pre-caregiv$ or caregiving or care-marital or spous$ or couple$ or daughter$ or son or son$2 or partner$ or husband$ or wife$ or wives or familial or families)).ti,ab. (2851)
12 (attachment adj2 (style$ or avoidant or insecure or secure or ambivalent)).ti,ab. (6161)
13 (coping adj2 (acceptance-based or solution-based or positive or abilit$ or strateg$ or style$ or mechanism$ or strain or stress)).ti,ab. (28270)
14 (social adj2 isolation).ti,ab. (5075)
15 (emotional adj2 (strain or impact or health or support)).ti,ab. (9683)

For peer review only

16 ((loneliness or social support or time to listen or befriend$ or lines of communication) and (carer$ or caregiver$ or care-giver$ or pre-caregiv$ or caregiving or care-marital or spous$ or couple$ or daughter$ or son or son$2 or partner$ or husband$ or wife$ or wives or familial or families)).ti,ab. (8486)
17 (respite adj2 (break$ or care)).ti,ab. (560)
18 caregivers/ or caregiver burden/ or elder care/ (24619)
19 *family relations/ (24979)
20 *interpersonal relationships/ or exp family conflict/ (14094)
21 intergenerational relations/ (3128)
22 parent child relations/ (23963)
23 emotional intelligence/ (3760)
24 *couples/ or *spouses/ (13434)
25 *marital status/ or *marriage/ (7397)
26 expressed emotion/ (1291)
27 "Sense of Coherence"/ (1168)
28 *coping behavior/ (31605)
29 social isolation/ (5821)
30 loneliness/ (3182)
31 *Social Support/ (20990)
32 respite care/ (401)
33 or/6-32 (249997)
34 5 and 33 (7103)
35 (authored book or book or edited book).pt. (409358)
36 scientific communication/ (21960)
37 case report/ (22551)
38 (letter or comment$).ti. (33887)
39 35 or 36 or 37 or 38 (482275)
40 (random$ or trial or blind$ or prognos$ or prospectiv$ or retrospectiv$ or "before and after" or interrupted time series or case control or cross-sectional or crossover or cross-over or cohort$ or meta-analys$ or systematic review or longitudinal).ti,ab. (464856)
41 39 not 40 (459334)
42 exp animals/ or animal models/ (290922)
43 (rat or rats or mouse or mice).ti. (96299)
44 or/41-43 (745544)
45 34 not 44 (6512)
46 meta analysis/ (3649)
47 (meta analy$ or metaanaly$ or metanaly$ or meta regression).ti,ab. (20935)
48 ((systematic$ or evidence$) adj2 (review$ or overview$)).ti,ab. (23038)
49 (reference list$ or bibliograph$ or hand search$ or manual search$ or relevant journals).ab. (17934)
50 (search strategy or search criteria or systematic search or study selection or data extraction).ab. (3271)
51 (search$ adj4 literature).ab. (6117)
52 (medline or pubmed or cochrane or embase or psychlit or psyclit or psychinfo or cinahl or science citation index or bids or cancerlit).ab. (13402)
53 cochrane.jw. (0)
54 ((multiple treatment$ or indirect or mixed) adj2 comparison).ti,ab. (124)
55 clinical trials/ (8640)
56 randomi#ed.ab. (48449)
57 random$.ti,ab. (141165)
58 placebo.ab. (31763)
59 randomly.ab. (54448)
60 trial.ti. (19386)
61 case control.ti,ab. (7274)
62 (cohort adj (study or studies or analys$)).ti,ab. (12120)

For peer review only

63 ((follow up or followup or observational or "before and after" or uncontrolled or non randomi#ed) adj (study or studies)).ti,ab. (17233)
64 ((longitudinal$ or prospectiv$) and (study or studies or studied or review$ or analys$ or cohort$)).ti,ab. (104849)
65 cross sectional.ti,ab. (45320)
66 interrupted time series.ti,ab. (494)
67 followup studies/ (12328)
68 cohort analysis/ (1118)
69 exp longitudinal studies/ (15563)
70 (crossover$ or cross-over$).ti,ab. (7590)
71 or/46-70 (394874)
72 prediction/ (17035)
73 prognosis/ (7200)
74 exp multivariate analysis/ (16733)
75 exp statistical regression/ (4806)
76 Statistical Analysis/ (14428)
77 statistical probability/ (4016)
78 item response theory/ (4659)
79 least squares/ (680)
80 treatment outcomes/ (26880)
81 disease course/ (9928)
82 "severity (disorders)"/ (13060)
83 (likelihood or probabilit$ or predict$ or prognos$ or progress$).ti,ab. (496092)
84 ((multivariate or regression) adj2 analys$).ti,ab. (71311)
85 logistic regression.ti,ab. (31604)
86 ((cox or linear or hazard$ or mixed or proportional or logistic) adj3 model$).ti,ab. (28503)
87 institutionalization/ (3346)
88 institutionali$.ti,ab. (13041)
89 or/72-88 (630258)
90 71 or 89 (906642)
91 45 and 90 (2901)

For peer review

WEB OF SCIENCE

# 31	3,896	#30 AND #19<br>*Indexes=SCI-EXPANDED, SSCI, CPCI-S, CPCI-SSH Timespan=All years*
# 30	6,282,730	#29 OR #28 OR #27 OR #26 OR #25 OR #24 OR #23 OR #22 OR #21 OR #20<br>*Indexes=SCI-EXPANDED, SSCI, CPCI-S, CPCI-SSH Timespan=All years*
# 29	266,576	TS= ((cox or linear or hazard* or mixed or proportional or logistic) near/3 model*)<br>*Indexes=SCI-EXPANDED, SSCI, CPCI-S, CPCI-SSH Timespan=All years*
# 28	336,935	TS= ((multivariate or regression) near/2 analys*)<br>*Indexes=SCI-EXPANDED, SSCI, CPCI-S, CPCI-SSH Timespan=All years*
# 27	3,829,258	TS= (likelihood or probabilit* or predict* or prognos* or progress* or "logistic regression" or institutionali*)<br>*Indexes=SCI-EXPANDED, SSCI, CPCI-S, CPCI-SSH Timespan=All years*
# 26	839,359	TS= (control* near/4 (group* or trial or study))<br>*Indexes=SCI-EXPANDED, SSCI, CPCI-S, CPCI-SSH Timespan=All years*
# 25	384,811	TS= (case-control or cross-sectional or "interrupted time series" or crossover* or cross-over*)<br>*Indexes=SCI-EXPANDED, SSCI, CPCI-S, CPCI-SSH Timespan=All years*

# 24	710,670	TS= ((cohort near/2 stud*) or (cohort near/2 analys*) or ("before and after" near/2 stud*) or (longitudinal* near/6 stud*) or (longitudinal* near/6 analys*) or (follow-up near/2 stud*) or (followup near/2 stud*) or (observational near/2 stud*) or (retrospectiv* near/6 stud*) or (retrospectiv* near/6 analys*) or (prospectiv* near/6 stud*) or (prospectiv* near/6 analys*) or (longitudinal* near/6 cohort*) or (retrospectiv* near/6 cohort*) or (prospectiv* near/6 cohort*))<br>*Indexes=SCI-EXPANDED, SSCI, CPCI-S, CPCI-SSH Timespan=All years*
# 23	264,330	TI= trial*<br>*Indexes=SCI-EXPANDED, SSCI, CPCI-S, CPCI-SSH Timespan=All years*
# 22	1,242,539	TS= random*<br>*Indexes=SCI-EXPANDED, SSCI, CPCI-S, CPCI-SSH Timespan=All years*
# 21	204,404	TS= ((controlled near/2 "clinical trial") or (doubl* near/2 blind*) or (singl* near/2 blind*) or (trebl* near/2 blind*) or (tripl* near/2 blind*))<br>*Indexes=SCI-EXPANDED, SSCI, CPCI-S, CPCI-SSH Timespan=All years*
# 20	287,295	TS= (metaanalys* or meta-analys* or "meta regression" or metanalys* or (systematic near/2 review*) or (systematic near/2 overview*) or (evidence* near/2 review*) or (evidence* near/2 overview*) or (search* near/4 literature*) or (hand near/2 search*) or (manual* near/2 search*) or (search near/2 strateg*) or (systematic* near/2 search*) or "study selection" or "data extraction" or "search criteria" or (indirect near/2 comparison) or (mixed near/2 comparison) or ("multiple treatment" near/2 comparison) or ("multiple treatments" near/2 comparison))<br>*Indexes=SCI-EXPANDED, SSCI, CPCI-S, CPCI-SSH Timespan=All years*
# 19	7,212	#15 not #18<br>*Indexes=SCI-EXPANDED, SSCI, CPCI-S, CPCI-SSH Timespan=All years*
# 18	2,175,105	#17 OR #16<br>*Indexes=SCI-EXPANDED, SSCI, CPCI-S, CPCI-SSH Timespan=All years*
# 17	198,475	TS= ((animal* near/2 experiment*) or (animal* near/2 model*) or (animal* near/2 laborator*))<br>*Indexes=SCI-EXPANDED, SSCI, CPCI-S, CPCI-SSH Timespan=All years*
# 16	2,020,675	TI= (rat or rats or mouse or mice or rodent* or comment* or letter or "case study" or "case report" or anecdote* or editorial* or news)<br>*Indexes=SCI-EXPANDED, SSCI, CPCI-S, CPCI-SSH Timespan=All years*
# 15	7,434	#14 AND #3<br>*Indexes=SCI-EXPANDED, SSCI, CPCI-S, CPCI-SSH Timespan=All years*
# 14	324,066	#13 OR #12 OR #11 OR #10 OR #9 OR #8 OR #7 OR #6 OR #5 OR #4<br>*Indexes=SCI-EXPANDED, SSCI, CPCI-S, CPCI-SSH Timespan=All years*
# 13	222,937	TS= ((carer* or caregiver* or care-giver* or pre-caregiv* or caregiving or care-giving or marital or spous* or couple* or daughter* or son or sons or partner* or husband* or wife* or wives* or family or families or familial) near/4 ("quality of life" or qol or conflict* or morbidity or capabilit* or depression or depressive or anxiety or closeness or motivat* or resilien* or communicat* or support* or counsel* or informal or factor* or distress* or relationship* or stress or burden* or interaction* or commitment or wellbeing or well-being or style* or dysfunctional or dependen* or independen* or intimat* or intimacy or affection* or tension* or pressure* or disagreement* or isolat* or loving))<br>*Indexes=SCI-EXPANDED, SSCI, CPCI-S, CPCI-SSH Timespan=All years*
# 12	57,045	TS= (relationship* near/3 (loving or intimat* or intimacy or affection* or quality or close* or dysfunctional or dependen* or predictor*))<br>*Indexes=SCI-EXPANDED, SSCI, CPCI-S, CPCI-SSH Timespan=All years*
# 11	4,080	TS= (attachment near/2 (style* or avoidant or insecure or secure or ambivalent))

		Indexes=SCI-EXPANDED, SSCI, CPCI-S, CPCI-SSH Timespan=All years
# 10	29,472	TS= (coping near/2 (acceptance-based or solution-based or positive or abilit* or strateg* or style* or mechanism* or strain or stress))<br>*Indexes=SCI-EXPANDED, SSCI, CPCI-S, CPCI-SSH Timespan=All years*
# 9	10,767	TS= (emotional near/2 (strain or impact or health or support))<br>*Indexes=SCI-EXPANDED, SSCI, CPCI-S, CPCI-SSH Timespan=All years*
# 8	619	TS= (respite near/2 (break or care))<br>*Indexes=SCI-EXPANDED, SSCI, CPCI-S, CPCI-SSH Timespan=All years*
# 7	5,581	TS= (social near/2 isolation)<br>*Indexes=SCI-EXPANDED, SSCI, CPCI-S, CPCI-SSH Timespan=All years*
# 6	21,636	TS= (("role captivity" or "self efficacy" or "sense of competence" or "sense of coherence" or loneliness or "social support" or "time to listen" or befriend* or "lines of communication") and (carer* or caregiver* or care-giver* or pre-caregiv* or caregiving or care-giving or marital or spous* or couple* or daughter* or son or sons or partner* or husband* or wife* or wives* or family or families or familial))<br>*Indexes=SCI-EXPANDED, SSCI, CPCI-S, CPCI-SSH Timespan=All years*
# 5	7	TS= "new york university caregiver intervention"<br>*Indexes=SCI-EXPANDED, SSCI, CPCI-S, CPCI-SSH Timespan=All years*
# 4	4,134	TS=(expressed near/2 emotion)<br>*Indexes=SCI-EXPANDED, SSCI, CPCI-S, CPCI-SSH Timespan=All years*
# 3	199,635	#2 OR #1<br>*Indexes=SCI-EXPANDED, SSCI, CPCI-S, CPCI-SSH Timespan=All years*
# 2	193,800	TI= (dementia or demented) or TS= alzheimer*<br>*Indexes=SCI-EXPANDED, SSCI, CPCI-S, CPCI-SSH Timespan=All years*
# 1	36,758	TS= ((dementia or demented) near/4 (elder* or senile or senility or person* or individual* or subject* or adult* or patient* or people*))<br>*Indexes=SCI-EXPANDED, SSCI, CPCI-S, CPCI-SSH Timespan=All years*

COCHRANE

Search Name: dementia_relation_prog
Date Run: 12/05/15 15:08:16.546
Description:

ID Search Hits
#1 MeSH descriptor: [Dementia] this term only 1443
#2 MeSH descriptor: [AIDS Dementia Complex] this term only 55
#3 MeSH descriptor: [Alzheimer Disease] this term only 2258
#4 MeSH descriptor: [Aphasia, Primary Progressive] explode all trees 8
#5 MeSH descriptor: [Dementia, Vascular] explode all trees 299
#6 MeSH descriptor: [Frontotemporal Lobar Degeneration] explode all trees 14
#7 MeSH descriptor: [Lewy Body Disease] this term only 48
#8 alzheimer*:ti,ab 5005
#9 (dementia or demented):ti 3405
#10 ((dementia or demented) near/3 (elder* or senile or senility or person* or subject* or adult* or patient* or people)):ti,ab 2209
#11 #1 or #2 or #3 or #4 or #5 or #6 or #7 or #8 or #9 or #10 8250
#12 MeSH descriptor: [Caregivers] this term only 1281
#13 MeSH descriptor: [Family Relations] this term only 183
#14 MeSH descriptor: [Family Conflict] this term only 47

For peer review only - http://bmjopen.bmj.com/site/about/guidelines.xhtml

#15 MeSH descriptor: [Intergenerational Relations] this term only 32
#16 MeSH descriptor: [Parent-Child Relations] this term only 719
#17 MeSH descriptor: [Emotional Intelligence] this term only 31
#18 MeSH descriptor: [Interpersonal Relations] this term only 1584
#19 MeSH descriptor: [Spouses] this term only 223
#20 MeSH descriptor: [Marriage] this term only and with qualifier(s): [Psychology - PX] 98
#21 MeSH descriptor: [Family] this term only and with qualifier(s): [Psychology - PX] 404
#22 MeSH descriptor: [Expressed Emotion] this term only 122
#23 MeSH descriptor: [Sense of Coherence] this term only 7
#24 MeSH descriptor: [Adaptation, Psychological] this term only 3138
#25 MeSH descriptor: [Social Isolation] this term only 141
#26 MeSH descriptor: [Loneliness] this term only56
#27 MeSH descriptor: [Social Support] this term only 2467
#28 MeSH descriptor: [Respite Care] this term only 33
#29 (coping near/2 (acceptance-based or solution-based or positive or abilit* or strateg* or style* or mechanism* or strain or stress)):ti,ab 1119
#30 (social near/2 isolation):ti,ab 157
#31 ((loneliness or "social support" or "time to listen" or befriend* or "lines of communication") and (carer* or caregiver* or care-giver* or pre-caregiv* or caregiving or care-giving or marital or spous* or couple* or daughter* or son or sons or partner* or husband* or wife* or wives* or familial or family or families)):ti,ab 486
#32 (emotional near/2 (strain or impact or health or support)):ti,ab 475
#33 (respite near/2 (break* or care)):ti,ab 28
#34 "new york university caregiver intervention":ti,ab 2
#35 (relationship* near/3 (loving or intimate or intimacy or affection* or quality or close* or dysfunctional or dependen* or predictor*)):ti,ab 714
#36 (expressed near/2 emotion):ti,ab 107
#37 (("role captivity" or "self efficacy" or "sense of competence" or "sense of coherence") and (carer* or caregiver* or care-giver* or pre-caregiv* or caregiving or care-giving or marital or spous* or couple* or daughter* or son or sons or partner* or husband* or wife* or wives* or familial or family or families)):ti,ab 496
#38 (attachment near/2 (style* or avoidant or insecure or secure or ambivalent)):ti,ab 77
#39 ((carer* or caregiver* or care-giver* or pre-caregiv* or caregiving or care-giving or marital or spous* or couple* or daughter* or son or sons or partner* or husband* or wife* or wives* or familial or family or families) near/4 ("quality of life" or Qol or morbidity or conflict* or capabili* or depression or depressive or anxiety or anxieties or closeness or motivat* or resilien* or communicat* or support* or counsel* or informal or factor* or distress* or relationship* or stress or burden* or interaction* or commitment or wellbeing or well-being or style* or dysfunctional or dependen* or independen* or intimat* or intimacy or affection* or tension* or pressure* or disagreement* or isolat* or loving)):ti,ab 4968
#40 #12 or #13 or #14 or #15 or #16 or #17 or #18 or #19 or #20 or #21 or #22 or #23 or #24 or #25 or #26 or #27 or #28 or #29 or #30 or #31 or #32 or #33 or #34 or #35 or #36 or #37 or #38 or #39 14181
#41 #11 and #40 849

For peer review only

PRISMA 2009 Checklist

Section/topic	#	Checklist item	Reported on page #
**TITLE**			
Title	1	Identify the report as a systematic review, meta-analysis, or both.	1
**ABSTRACT**			
Structured summary	2	Provide a structured summary including, as applicable: background; objectives; data sources; study eligibility criteria, participants, and interventions; study appraisal and synthesis methods; results; limitations; conclusions and implications of key findings; systematic review registration number.	2
**INTRODUCTION**			
Rationale	3	Describe the rationale for the review in the context of what is already known.	3-4
Objectives	4	Provide an explicit statement of questions being addressed with reference to participants, interventions, comparisons, outcomes, and study design (PICOS).	4
**METHODS**			
Protocol and registration	5	Indicate if a review protocol exists, if and where it can be accessed (e.g., Web address), and, if available, provide registration information including registration number.	4
Eligibility criteria	6	Specify study characteristics (e.g., PICOS, length of follow-up) and report characteristics (e.g., years considered, language, publication status) used as criteria for eligibility, giving rationale.	4-6
Information sources	7	Describe all information sources (e.g., databases with dates of coverage, contact with study authors to identify additional studies) in the search and date last searched.	6
Search	8	Present full electronic search strategy for at least one database, including any limits used, such that it could be repeated.	6
Study selection	9	State the process for selecting studies (i.e., screening, eligibility, included in systematic review, and, if applicable, included in the meta-analysis).	6
Data collection process	10	Describe method of data extraction from reports (e.g., piloted forms, independently, in duplicate) and any processes for obtaining and confirming data from investigators.	6-7
Data items	11	List and define all variables for which data were sought (e.g., PICOS, funding sources) and any assumptions and simplifications made.	6
Risk of bias in individual studies	12	Describe methods used for assessing risk of bias of individual studies (including specification of whether this was done at the study or outcome level), and how this information is to be used in any data synthesis.	7
Summary measures	13	State the principal summary measures (e.g., risk ratio, difference in means).	6
Synthesis of results	14	Describe the methods of handling data and combining results of studies, if done, including measures of consistency (e.g., I^2) for each meta-analysis.	7

For peer review only - http://bmjopen.bmj.com/site/about/guidelines.xhtml

PRISMA 2009 Checklist

Section/topic	#	Checklist item	Reported on page #
Risk of bias across studies	15	Specify any assessment of risk of bias that may affect the cumulative evidence (e.g., publication bias, selective reporting within studies).	3
Additional analyses	16	Describe methods of additional analyses (e.g., sensitivity or subgroup analyses, meta-regression), if done, indicating which were pre-specified.	7
**RESULTS**			
Study selection	17	Give numbers of studies screened, assessed for eligibility, and included in the review, with reasons for exclusions at each stage, ideally with a flow diagram.	8
Study characteristics	18	For each study, present characteristics for which data were extracted (e.g., study size, PICOS, follow-up period) and provide the citations.	Tables
Risk of bias within studies	19	Present data on risk of bias of each study and, if available, any outcome level assessment (see item 12).	Table 1
Results of individual studies	20	For all outcomes considered (benefits or harms), present, for each study: (a) simple summary data for each intervention group (b) effect estimates and confidence intervals, ideally with a forest plot.	Tables
Synthesis of results	21	Present the main results of the review. If meta-analyses are done, include for each, confidence intervals and measures of consistency	Tables plus page 11,13,15
Risk of bias across studies	22	Present results of any assessment of risk of bias across studies (see Item 15).	Not done (see p17)
Additional analysis	23	Give results of additional analyses, if done (e.g., sensitivity or subgroup analyses, meta-regression [see Item 16]).	n/a
**DISCUSSION**			
Summary of evidence	24	Summarize the main findings including the strength of evidence for each main outcome; consider their relevance to key groups (e.g., healthcare providers, users, and policy makers).	16 (16-19)
Limitations	25	Discuss limitations at study and outcome level (e.g., risk of bias), and at review-level (e.g., incomplete retrieval of identified research, reporting bias).	17
Conclusions	26	Provide a general interpretation of the results in the context of other evidence, and implications for future research.	19
**FUNDING**			
Funding	27	Describe sources of funding for the systematic review and other support (e.g., supply of data); role of funders for the systematic review.	21

From: Moher D, Liberati A, Tetzlaff J, Altman DG, The PRISMA Group (2009). Preferred Reporting Items for Systematic Reviews and Meta-Analyses: The PRISMA Statement. PLoS Med 6(7): e1000097. doi:10.1371/journal.pmed1000097

For peer review only - http://bmjopen.bmj.com/site/about/guidelines.xhtml

PRISMA 2009 Checklist

For more information, visit: www.prisma-statement.org.

Page 2 of 2

For peer review only

Quality of family relationships and outcomes of dementia: a systematic review

Journal:	*BMJ Open*
Manuscript ID	bmjopen-2016-015538.R1
Article Type:	Research
Date Submitted by the Author:	06-Sep-2017
Complete List of Authors:	Edwards, Hannah; University of Bristol, School of Social and Community Medicine<br>Ijaz , Sharea ; NIHR CLAHRC West<br>Whiting, Penny; University of Bristol, School of Social and Community Medicine<br>Leach, Verity; University of Bristol<br>Richards, Alison; University of Bristol, School of Social and Community Medicine<br>Cullum, Sarah; University of Bristol, School of Social and Community Medicine<br>Cheston, Richard; University of the West of England, Health and Social Sciences<br>Savovic, Jelena; University of Bristol, NIHR CLAHRC West
**Primary Subject Heading**:	Neurology
Secondary Subject Heading:	Evidence based practice, Health services research, Patient-centred medicine, Epidemiology, Mental health
Keywords:	Dementia < NEUROLOGY, family relationships, challenging behaviour, institutionalisation, systematic review

SCHOLARONE™ Manuscripts

Title

Quality of family relationships and outcomes of dementia: a systematic review

Author details

Corresponding author: Jelena Savović, Bristol Medical School, University of Bristol, and National Institute for Health Research (NIHR) Collaboration for Leadership in Applied Health Research and Care (CLAHRC) West.

Email: j.savovic@bristol.ac.uk

Telephone: 0117 342 1243

Address: 9th Floor Whitefriars, Lewins Mead, Bristol BS1 2NT, UK

Full author list: *Hannah B Edwards[1,2], *Sharea Ijaz[1,2], Penny Whiting[1,2], Verity Leach[1,2], Alison Richards[1,2], Sarah Cullum[3], Richard Cheston[4], Jelena Savović[1,2]

1. Bristol Medical School, University of Bristol. Bristol, UK
2. National Institute for Health Research (NIHR) Collaboration for Leadership in Applied Health Research and Care (CLAHRC) West. Bristol, UK
3. University of Auckland. Auckland, New Zealand.
4. Health and Social Sciences, University of the West of England. Bristol, UK

* Joint first authors

Keywords: Dementia, carers, family relationships, institutionalisation, challenging behaviour

Word count: 3746

Tables: 5

Figures: 1

For peer review only - http://bmjopen.bmj.com/site/about/guidelines.xhtml

ABSTRACT

Objectives: Evaluation of the association between the quality of relationship between a person with dementia and their family carer and outcomes for the person with dementia.

Design: Systematic review.

Eligibility criteria: Cohort studies of people with clinically diagnosed dementia and their main carers. Exposures of interest were any elements of relationship quality, e.g. attachment style, expressed emotion, coping style. Our primary outcome was institutionalisation, and secondary outcomes were hospitalisation, death, quality of life, and behavioural and psychiatric symptoms of dementia (‘challenging behaviour’).

Data sources: Medline, Embase, Web of Science, PsycInfo, the Cochrane Library and Opengrey were searched from inception to May 2017.

Study appraisal and synthesis methods: The Newcastle-Ottawa Scale was used to assess risk of bias. A narrative synthesis of results was performed due to differences between studies.

Results: Twenty studies were included. None of the studies controlled for all pre-specified confounding factors (age, gender, socio-economic status, and severity of dementia). Reporting of results was inadequate with many studies simply reporting whether or not associations were ‘statistically significant’ without providing effect size estimates or confidence intervals. There was a suggestion of an association between relationship factors and global challenging behaviour. All studies evaluating global challenging behaviour provided statistical evidence of an association (most p-values below 0.02). There was no consistent evidence for an association for any other outcome assessed.

Conclusions: There is currently no strong or consistent evidence on the effects of relationship factors on institutionalisation, hospitalisation, death or quality of life for people with dementia. There was a suggestion of an association between relationship factors and challenging behaviour, although the evidence for this was weak. To improve our ability to support those with dementia and their families further robust studies are needed.

For peer review only - http://bmjopen.bmj.com/site/about/guidelines.xhtml

For peer review only

Systematic review registration: CRD42015020518

ARTICLE SUMMARY

Strengths and limitations of this study

- Broad search strategy so unlikely to have missed relevant studies
- Double screening minimises selection bias
- We were not able to assess publication bias and the potential for selective reporting of outcomes within studies.

BACKGROUND

Dementia is a key public health concern in the UK[1-3] with around 7% of all those over 65 affected, and the numbers of people with dementia predicted to double every 20 years.[4-6] Institutionalisation (being placed in a full time care /nursing home) is a key outcome for people with dementia, their families, and the healthcare system. Although in many circumstances institutionalisation may be the best or only option for the person affected by dementia, most people report that they would prefer to stay living in their own home.[7] Recent media attention to a few very poorly run care homes has also lead to concerns about institutionalisation.[8-10] Additionally, the financial cost of full-time care is very high, both for affected individuals and their families, and for the public, as public taxes are used to contribute to care home fees. Consequently, for some time it has been UK government policy to help families to continue supporting people with dementia at home, specifically to delay or avoid institutionalisation.[11]

The quality of relationship between the person with dementia and people who care for them has been linked with a range of outcomes including institutionalisation, cognitive and functional decline, and quality of life.[12-21] There is also growing interest in the potential for psychosocial interventions to improve outcomes, by enhancing interactions within families.[22 23] If families are better equipped to

For peer review only

For peer review only - http://bmjopen.bmj.com/site/about/guidelines.xhtml

cope with the psychological and emotional challenges of dementia, then care at home may be sustained for longer. However, it is not clear which elements of the relationship are predictive of early institutionalisation or which lead to a faster decline. This evidence is necessary both to justify and to help to develop early psychosocial interventions, ideally at the point of diagnosis.

To address this issue we performed a systematic review of the evidence on how elements of relationship quality between the person with dementia and their main informal (family) carer are associated with outcomes for the person with dementia.

METHODS

This study was a systematic review, registered with the PROSPERO International Prospective Register of systematic reviews, registration number CRD42015020518. The full protocol has been published.[24]

Eligibility criteria

Only cohort studies (prospective and retrospective) were included in the review. Relevant systematic reviews were obtained and used as a means of identifying other original studies.[24] Qualitative, case control (unless nested in a prospective cohort), and cross-sectional studies were excluded.

The population of interest was people with dementia and their main informal caregiver (most commonly a spouse or child). Professional, paid caregivers were excluded. People with all types of clinically-diagnosed dementia were included.

The exposures of interest were factors that capture an element of relationship quality. We adopted a broad definition of “relationship quality” as how happy or satisfied an individual is in his or her relationship. [25] Attachment style, coping style, affection, and expressed emotion were all identified

For peer review only - http://bmjopen.bmj.com/site/about/guidelines.xhtml

at the design stage as key exposures of interest. While affection is a relatively straightforward term, attachment style, coping and expressed emotion relate to specific psychological constructs. ‘Attachment style’ is a term originally developed to understand the emotional relationship between children and parents, but has since been extended to adult romantic relationships. Four main styles of attachment have been identified in adults: secure; anxious–preoccupied; dismissive-avoidant; and fearful-avoidant.[26 27] Coping is a wide-ranging construct that includes elements which are clearly measures of relationship quality (e.g. ‘relationship-focussed coping’) and those which are more individual in nature (e.g. acceptance coping). However, as even individual coping styles are typically initiated in response to aspects of relationships, we felt that this was an appropriate exposure to capture. ‘Expressed Emotion’ (EE) is a measure of the family environment based on how the relatives of a psychiatric patient spontaneously talk about/to the patient. High levels of expressed emotion have been associated with worse prognosis in a number of mental illnesses including schizophrenia. This may be due to emotional over-involvement, which can be experienced as hostility, criticism and intolerance.[28 29]

Two other notable factors emerged as key exposures when reviewing the literature: ‘mutuality’ and ‘boundary ambiguity’. Mutuality is a cluster concept capturing levels of positive engaging interaction, attachment and emotional support. Boundary ambiguity involves uncertainty about whether a person is in or out of the family group. This occurs as a result of significant changes in that person including those cognitive, functional, mood and personality changes that are indicative of dementia. Boundary ambiguity is associated with, and taken as an indicator of emotional distancing and the withdrawal of the caregiver from the person with dementia. Other factors emerging from the literature were included if they captured an element of relationship quality, and this was assessed on a case-by-case basis through discussion with the study team. Carer abuse was excluded, as this was considered to be a different area of research. Overall measures of carer burden

For peer review only - http://bmjopen.bmj.com/site/about/guidelines.xhtml

were also excluded as an exposure because they relate more to cognitive and functional levels in dementia than to relationship quality.

The primary outcome of interest was institutionalisation. This is a key event in the course of dementia, both socially and financially, from the perspectives of the individual, their family and the public. Secondary outcomes were hospitalisations, death (or time to death), quality of life (QoL), and challenging behaviour (also referred to as the behavioural and psychological symptoms of dementia or BPSD). Examples of challenging behaviour can include depression, anxiety, aggression, paranoia, hallucinations and delusions. Studies measuring QoL or challenging behaviour as the outcome had to use validated assessment tools to be included in the review.

Search strategy

Medline, Embase, Web of Science, PsycInfo, The Cochrane Library and Opengrey were searched from inception to May 2017 without any language restrictions. The full search strategy is available in the supplementary data of our published protocol.[24] All results were imported into an Endnote X7 reference library, and into a bespoke-built Microsoft Access 2013 database to manage screening.

Selection of papers

The titles and abstracts of all identified papers were screened in duplicate by two reviewers working independently, and all potentially relevant papers were retrieved. All retrieved papers were read in full and assessed for eligibility using a standardised and piloted inclusion checklist, applied by two reviewers independently. Any discrepancies between the reviewers (at either stage of screening) were resolved through discussion.

For peer review only - http://bmjopen.bmj.com/site/about/guidelines.xhtml

Data extraction

Data were extracted from included studies using a bespoke data collection form, which was piloted on six studies and amended as a result of the piloting. Data extracted included study characteristics, characteristics of the population of people with dementia and their carers, recruitment and response, the exposures and outcomes and how and when they were measured, and details of the analyses and results. The terminology for risk factors and outcomes was used as reported by the original study authors. Where numerical results were incompletely reported, where possible, relevant results (effect estimates, standard errors, 95% confidence intervals) were calculated from the raw data. Multiple publications of the same dataset were counted as a single study. In the case where analyses of the same association was repeated in more than one report, our policy was either to include the result based on the largest sample size only, or if sample sizes were equivalent, then we would include the result from the most recent publication only. Data were extracted from the published reports only, we did not contact authors for additional unpublished information.

Analysis

We planned to use meta-analysis to estimate summary effect sizes if there had been sufficient studies with similar populations, exposures and outcomes. As meta-analysis was not possible, a narrative synthesis of results is provided.[24]

Assessment of methodological quality and risk of bias

The Newcastle-Ottawa Scale[30] (NOS) was used to assess risk of bias for included reports. This is an eight-item questionnaire that assesses the following methodological criteria: representativeness of the exposed cohort; selection of the non-exposed cohort; ascertainment of exposure; demonstration that the outcome of interest was not present at the start of the study; comparability of cohorts (risk of confounding); assessment of exposure and outcome; and length and adequacy of follow up. NOS allocates 'stars' for adequate methods, but does not specifically advise calculating

For peer review only - http://bmjopen.bmj.com/site/about/guidelines.xhtml

the sum of allocated stars to give an overall score.[30] Empirical evidence also suggests that numerical quality scores are not helpful in differentiating between studies of high and low risk of bias.[31] For this reason we considered each of the eight criteria of the NOS tool separately and assessed the study as having adequate methods for that particular aspect of study conduct if the 'star' could be allocated for that NOS criterion. In our protocol we considered ten factors as potentially important confounding domains.[24] During the piloting of the data-extraction and risk of bias assessment the team agreed a minimum number of essential confounders that all studies should have adjusted for. A study had to control for the following four pre-specified factors to be at low risk of confounding: age, gender, socio-economic status (SES) and dementia severity. We also recorded other confounders studies have adjusted for, in addition to the four main confounders used for risk of bias assessment.

RESULTS

The search identified 9,321 potentially relevant papers. Of these, 190 papers were retrieved for full-text screening, 23 publications [20 32-53] met the eligibility criteria (Figure 1). Four of the 23 publications were based on data from one cohort study[32-35] and so the total number of unique studies was 20 (2340 participants with dementia). As the four connected publications each contributed unique results, all were included in the review.

Study characteristics

The majority of included unique studies (14 out of 20) came from the US, and there was one each from the UK, Canada, Switzerland, Belgium, the Netherlands, and Australia. Sample sizes in the relevant analyses ranged from 29 to 220 (mean 143). Years of publication ranged from 1990 to 2016, with most from the 1990s and early 2000s. The most frequently reported dementia type was Alzheimer's disease, although in eight studies the distribution of dementia types was not reported. Time since diagnosis ranged from 3 months to 6.5 years, although in six studies this was not reported. The majority (13/20) did not report participants' ethnicity, where this was reported,

For peer review only - http://bmjopen.bmj.com/site/about/guidelines.xhtml

cohorts were predominantly Caucasian. The majority of caregivers included were spouses (100% in seven studies, and more than 50% in another seven). Other carers were children of the person with dementia. The characteristics of included studies are presented in Table 1.

For peer review only

For peer review only - http://bmjopen.bmj.com/site/about/guidelines.xhtml

Table 1. Characteristics of included studies

Study ID	Country	People with dementia						Carers					Exposures*	Outcomes*
		N	% female	dementia type	Dementia duration years (SD); range	Dementia severity Mean (SD); range	Age years (SD); range	N	% female	% spouse	% child	Age years (SD); range		
Caron 1999[36]	USA	60	22	AD	3.4 (2.5);1-13	NR	68.26 (8.44); 53-90	60	79	86	8	63.57 (8.94; 30-84	Boundary ambiguity (caregiver closeout)	BPSD – depression; anxiety; paranoia; activity disturbance
Clare 2013 [37]	UK	51	50.98	61% AD, 21% VD, 17% Mixed	NR	MMSE= 24.5 (2.80); 18-30	76.75 (7.88); 55-91	51	70	57	31	65.06 (14.50); 33-89	positive quality of relationship: Positive Affect Index	QoL Alzheimer's Disease Scale (QoL-AD)
Kunik 2010 (4 papers) [32-35]	USA	215	4.6	NR	≤ 1	NR	76 (6.2)	215	NR	NR	NR	NR	Mutuality, relationship strain	BPSD – aggression, depression, psychosis, hospitalisation
Spruytte 2001[38]	Belgium	144	68.8	NR	≥ 0.25	GDS mean= 6	82; 61-94	144	69.4	38.9	50.7	63; 38-90	Quality of relationship; criticism	Institutionalisation
Fisher 1999[39]	USA	164	0.44	NR	6.5	MMSE= 17 (6.25)	77 (7)	164	54% non NHP, 47% NHP	0	100	45 (9)	Emotional Closeness; Boundary ambiguity; Negative family feelings	Institutionalisation
Burgener 2002 [20]	USA	96	57	NR	≤ 1	MMSE= 21.52; 10-29	77.3 (7.8); 55-96	96	74	56	40	63.7 (12.2); 35-83	Quality of relationship	Institutionalisation
Stevens 2004 [40]	USA	215	71.2	NR	NR	MMSE= 12.2 (8.7)	74.4 (8.2)	215	NR	47.9	40.5	NR	Coping strategies	Institutionalisation

For peer review only

For peer review only - http://bmjopen.bmj.com/site/about/guidelines.xhtml

Perren 2007[41]	Switzerland	68	NR	60% AD, 25% VD, 15% other	NR	MMSE = 21.6 (5.1)	74.8 (7.6)	68	NR	100	0	70.9 (10.0)	attachment style	Overall care recipients problem behaviour
Wright 1994[42]	USA	29		AD	4.8; 1-11	MMSE = 18; 4-22, GDS = 4.4; 2-6	67.5; 51-83	30	80	100	0	67.4; 51-81	Cohesion; Tension; affection; present/past marital happiness	Institutionalisation
Wright 1998[43]	USA	14	50	AD	2.8	MMSE of 21.29 (3.69); 16-26	65; 49-85	14	50	100	0	NR	Cohesion; tension; affection	BPSD - Depression
Vitaliano 1993[44]	USA	77	32	AD	4.3 (2.1)	NR	70.9 (6.9)	77	NR	100	0	67.2 (7.4)	Expressed Emotion	Depression; negative behaviour
Bannister 1998[45]	UK	116	72.4	72% probable AD, 17% VD, 10% LB, 3% unclear	NR	CAMCOG mean= 45.2	79.8	116	59.5	14.7	NR	65	Coping strategies	Institutionalisation
de Vugt 2004[46]	The Netherlands	99	56.6	74% AD, 19% VD, 2% frontal lobe, 3% Parkinson's, 2% mixed	3.5 (31.1)	MMSE= 18.2 (4.8)	78.2 (8.4)	99	66.6	55.6	44.4	61.9 (11.9)	Coping strategies	psychosis; mood/apathy; hyperactivity; overall BPSD
Wells 1998[47]	Australia	93	42	NR	5.6 (4.2)	NR	76.1 (7.3) men; 74.6 (6.2) women	93	58	100	0	74.7 (6.2) men; 71.4 (7.9) women	Coping strategies	Institutionalisation
Torossian 1999[48]	USA	197	60.9	AD	NR	GDS range= 4-7	NR	197	39.1	100	0	70.7	adaptability; cohesion	institutionalization

For peer review only - http://bmjopen.bmj.com/site/about/guidelines.xhtml

Markiewicz 1997[49]	Canada	108	56.6	92% AD, 8% dementia + stroke	3.43 (2.57)	MMSE= 12.86 (7.95)	74.42 (8.37)	108	68.1	65.5	34.5	62.24 (12.97)	attachment styles	institutionalisation
Pruchno 1990[50]	USA	220	NR	AD	NR	NR	NR	220	67.9	100	0	70.2; 45-94	Quality of relationship	Institutionalisation
McClendon 2004 [51]	USA	141	45	71.5% probable AD, 28.5% possible AD	4.14 (2.47)	MMSE= 17.28 (6.50)	72.46 (7.91)	141	NR	75	NR	NR	Caregiver coping	Time to death
Shroff 2015[52]	USA	NR	NR	NR	NR	NR	84.78 (7.78)	83	85.5	54.3	NR	62.38 (18.21)	family coping and coherence	PwD Qol
Snyder 2016[53]	USA	233	56.7	NR	3.49 (1.80)	NR	86.08 (5.83)	233	78.5	37.3	53.6	66.27 (13.21)	carer coping strategies	incidence of severe dementia, time to institutionalisation, mortality

AD= Alzheimer's dementia; BPSD= Behavioural and Psychological Symptoms of Dementia; CAMCOG= Cambridge Cognition score; CBA= controlled before and after study; DRS= Dementia Rating Scale; GDS= Global Deterioration Scale; MMSE= mini mental state examination; N= numbers in analysis; NR= Not reported; SD= standard deviation; PwD= person with dementia; QoL= quality of life; RCT= randomised controlled trial; NHP= nursing home placement; VD= vascular dementia

*The exposures and outcomes listed in table refer only to the ones relevant to our review question

Study design: All studies were prospective cohorts except for Godwin 2014 (randomized trial) and Scroff 2015 (controlled before & after intervention study) - both were analysed as prospective cohorts disregarding intervention status as the focus of the review were associations of relationship risk factors rather than interventions effectiveness.

For peer review only - http://bmjopen.bmj.com/site/about/guidelines.xhtml

Risk of bias in included studies (Table 2)

None of the studies met all eight NOS criteria. All studies had adequate ascertainment of exposure as these were all based on structured interviews. All but one also had adequate ‘demonstration that the outcome of interest was not present at start of study’ and ‘follow up long enough for outcome to occur’. Blind independent outcome assessment was not possible in many studies with institutionalisation as the outcome because this outcome tended to be self-reported by the carer.

In total, 60 separate analyses were included in this review, of which 40 (two thirds) did not control for any potential confounding factors. In the 20 analyses that did include some adjustment, only a minority adjusted for any of the factors we identified as key potential confounders. None of the studies adjusted for all four key confounding factors. Three studies each adjusted for three out of the four key confounding factors.[33 50 53] A further two studies adjusted for two of the four identified key confounders.[46 47]

Seven studies had inadequate follow up of the cohort (loss to follow up). [37 47 48 50-53] Many studies presented insufficient information to make a clear judgment on some of the NOS criteria. In addition to potential risk of bias, many studies had reporting problems. Of the 60 included results, eight (13%) neither reported effect size estimates nor confidence intervals, 40 (67%) reported an effect size with no confidence intervals, and 23 (38%) did not report specific p-values. Only six analyses (10%) fully reported their results with effect size, confidence intervals, and specific p-values.

For peer review only - http://bmjopen.bmj.com/site/about/guidelines.xhtml

Table 2: Risk of bias assessment based on Newcastle –Ottawa scale

Study Author Year	Country	Newcastle-Ottawa Scale items addressing different methodological components of the study and potential sources of bias							
		Representativeness of the exposed cohort	Selection of the non-exposed cohort	Ascertainment of exposure	Outcome of interest was not present at start of study	Comparability of cohorts on the basis of the design/analysis (confounding)	Assessment of outcome blind/ record linkage	Follow-up long enough for outcome to occur	Adequacy of follow up of cohorts (attrition)
Pruchno 1990 [50]	USA	✓	✓	✓	✓	×[1]	×	✓	×
Vitaliano 1993 [44]	USA	✓	✓	✓	✓	×	×	✓	✓
Wright 1994 [42]	USA	✓	✓	✓	✓	×	✓	✓	✓
Markiewicz 1997 [49]	Canada	✓	✓	✓	✓	×	✓	✓	✓
Bannister 1998 [45]	UK	✓	✓	✓	✓	×	×	✓	✓
Wells 1998 [47]	Australia	✓	✓	✓	✓	×	×	✓	×
Wright 1998 [43]	USA	×	×	✓	✓	×	×	✓	✓
Caron 1999 [36]	USA	×	×	✓	✓	×	×	✓	✓
Fisher 1999 [39]	USA	✓	✓	✓	✓	×	×	✓	✓
Torrossian 1999 [48]	USA	×	✓	✓	✓	×	×	✓	×
Spruytte 2001 [38]	Belgium	✓	✓	✓	✓	×	×	×	✓
Burgener 2002 [20]	USA	✓	✓	✓	✓	×	×	✓	✓
de Vugt 2004 [46]	Netherlands	✓	✓	✓	×	×	×	✓	✓
McClendon 2004 [51]	USA	×	✓	✓	✓	×	✓	✓	×
Stevens 2004 [40]	USA	✓	✓	✓	✓	×	×	✓	✓
Perren 2007 [41]	Switzerland	✓	✓	✓	✓	×	×	✓	✓
Kunik 2010 (4 papers) [32-35]	USA	✓	✓	✓	✓	×[1]	×	✓	✓
Clare 2014 [37]	UK	×	✓	✓	✓	×	×	✓	×
Shroff 2015[52]	USA	✓	✓	✓	✓	×	×	×	×
Snyder 2016[53]	USA	✓	✓	✓	✓	×[1]	✓	✓	×

✓= study dealt with this adequately. ×= study was at risk of bias in this area, or provided no information to demonstrate otherwise.

[1.] These studies adjusted for three out of the four key confounders

For peer review only - http://bmjopen.bmj.com/site/about/guidelines.xhtml

Primary outcome: risk factors for institutionalisation (Table 3)

Ten studies examined 25 different relationship quality factors as potential risk factors for institutionalisation. Follow-up ranged from 6 to 24 months. The majority of studies found no association between the risk factors investigated and the incidence of institutionalisation. Although some individual studies reported associations between relationship quality and institutionalisation, there were no consistent findings across risk factors, and the lack of appropriate adjustment for basic confounding factors makes interpreting the results very difficult.

For peer review only

Table 3: Associations between relationship factors and institutionalisation (10 studies)

Risk factor	Study	N	Follow-up	Results (95% CI; p-value)	Analysis adjusted for[1]
**Factors relating to the interaction between the person with dementia and their caregiver**					
Quality of relationship	Spruytte[38]	144	6-9m	OR 0.92 (p=0.02)	NR
	Pruchno[50]	220	12m	OR 1.31 (p>0.05)	a,g,s,r,t,c,bp,ADL, ci,m,hs,cb
Marital happiness<br>At baseline	Wright[42]	29	24m	PoV 0.313 (p<0.01)	NR
Before dementia onset				PoV 0.045 (p>0.05)	NR
Emotional closeness	Fisher[39]	164	24m	OR 1.64 (1.09, 2.46; p=0.02)	d,ba,nf, oc,fe
Emotional distancing /boundary ambiguity	Fisher[39]	164	24m	OR 1.25 (0.85, 1.83; p=0.26)	d,ba,nf,oc,fe
	Wells[47]	93	12-18m	OR 1.3 (p>0.05)	a,d,t
Cohesion	Torossian[48]	197	24m	MD NR (p>0.05)	NR
	Wright[42]	29	24m	PoV 0.472 (p<0.001)	NR
Affection	Wright	29	24m	PoV 0.144 (p>0.05)	NR
Warmth*	Spruytte[38]	144	6-9m	NR (p<0.05)	NR
Anxious-ambivalent attachment (carer)	Markiewicz[49]	108	24m	OR 1.27 (p=0.36)	NR
Avoidant attachment (carer)	Markiewicz[49]	108	24m	OR 2.39 (p<0.001)	NR
	Stevens[40]	215	24m	HR 1.011 (p=0.37)	NR
High levels of tension	Wright[42]	29	24m	PoV 0.162 (p>0.05)	NR
Excessive criticism*	Spruytte[38]	144	6-9m	NR (p>0.05)	NR
**Factors mainly relating to the caregiver**					
Limited adaptability	Torossian[48]	197	24m	MD NR (p>0.05)	NR
Approach coping	Stevens[40]	215	24m	HR 0.997 (p=0.77)	NR
Directing relative's behaviour	Bannister[45]	116	12m	MD 0.2 (p=0.40)	NR
Keeping relative busy	Bannister[45]	116	12m	MD 0.5 (p=0.02)	NR
Learning about the illness	Bannister[45]	116	12m	MD 0.3 (p=0.42)	NR
Prioritizing	Bannister[45]	116	12m	MD 0.1 (p=0.52)	NR
Reducing expectations	Bannister[45]	116	12m	MD 0.4 (p=0.19)	NR
Consistent larger sense of the illness	Bannister[45]	116	12m	MD 0.4 (p=0.35)	NR
Positivity	Bannister[45]	116	12m	MD 0.5 (p=0.57)	NR
	Wells[47]	93	12-18m	OR 1.03 (p>0.05)	a,d
Seeking social support	Wells[47]	93	12-18m	OR 1.91 (p<0.05)	a,d
	Snyder [53]	233	NR	HR 1.159 (0.718, 1.87; p=0.55)	a,d,g,nc
Accepting responsibility	Wells[47]	93	12-18m	OR 0.28 (p<0.01)	a,d
Confrontational	Wells[47]	93	12-18m	OR 2.12 (p<0.05)	a,d
Negative feelings	Fisher[39]	164	24m	OR 1.47 (0.99, 2.19; p=0.05)	d,ba,oc,ec,fe

Legend: **OR** = odds ratio. OR>1 indicates increased risk, OR<1 indicates decreased risk. **HR** = hazard ratio, interpreted the same as OR. **PoV** = proportion of variance. This is a number between 0-1 indicating the proportion of variance in the outcome explained by the exposure. Higher numbers indicate greater explanatory power of the exposure. **Corr** = correlation. This is a number between -1 to +1 and indicates the degree to which the exposure and outcome vary together. Positive numbers indicate that exposure and outcome increase together, negative numbers indicate that the outcome increases as the exposure decreases. Larger numbers indicate stronger correlation. **MD** = mean difference in outcome. Large differences between groups suggests that the exposure might affect the outcome. **NR** = information not reported (where possible results and 95%CIs were calculated from raw data)

1. Pre-specified key confounders: **a** = age; **g** = gender; **s** = socioeconomic status; **d** = dementia severity.

All other confounders: ADL= Activities of Daily Living; ba=Boundary ambiguity; bp= Behaviour Problems; c=number of children; ci= caregiver illness; hs=help services used; ,cb= caregiver burden; ec= Emotional Closeness; fe= Family efficiency;

For peer review only - http://bmjopen.bmj.com/site/about/guidelines.xhtml

m= medication; nc= non-coresidency; nf=negative feeling; oc= Organised cohesiveness; r= religion; t= time spent (duration of) caregiving;
*These are the subscales of the relationship quality scale

Risk factors for challenging behaviour (BPSD) (Table 4)

Under the umbrella term of challenging behaviour four main types of outcome were evaluated: global challenging behaviour scores; psychotic symptoms; depression; and other BPSD outcomes. Eight studies examined nine different relationship quality factors as potential risk factors for these aspects of challenging behaviour. The length of follow-up ranged from 6 to 24 months.

There was a suggestion of an association between relationship factors and global challenging behaviour. All studies evaluating global challenging behaviour provided statistical evidence of an association (most p-values below 0.02). However, one study that reported two analyses did not report effect sizes.[46] For another the reported effect size was very small (mean difference of 0.23 in a scale of 1-144).[41] A larger effect size was seen for the association between expressed emotion and global challenging behaviours (mean difference of 1.9 in a scale of 0-8).[44] None of these analyses adjusted for our pre-specified confounding factors.

Most studies found no evidence of an association between relationship factors and either psychotic symptoms, depression or other BPSD outcomes. However, some of these were small studies that may have been underpowered to detect an association. One study adjusted for three out of four pre-specified potential confounders.[33] This was also one of the largest studies (n=171). It found no evidence for an association between couple mutuality and psychotic symptoms and a very weak, likely clinically negligible effect of this factor on depression (mean difference -0.43 points on a scale of 0-68).[33]

For peer review only - http://bmjopen.bmj.com/site/about/guidelines.xhtml

Table 4: Associations between relationship factors and Challenging behaviour (BPSD) (8 studies)

Risk factor	Study	N	Follow-up	Results (95% CI; p-value)	Analyses adjusted for[1]	Outcome assessment tool, scale range[2]
**Outcome: Global challenging behaviour (BPSD), or "problem behaviour" (4 studies)**						
Emotional distancing /boundary ambiguity	Caron[36]	60	12m	Corr 0.27 (p<0.001) (overall)	NR	BEHAVE-AD, 0-75
Non-adaptive coping	deVugt[46]	69	6m	MD NR (p=0.007)	g,s,ct	NPI, 1-144
		69	12m	MD NR (p=0.019)	g,s,ct	
Avoidant attachment	Perren[41]	68	24m	MD 0.23 (p<0.05)	NR	NPI, 1-144
Expressed emotion	Vitaliano[44]	77	15-18m	MD 1.9 (0.77, 3.04; p<0.001)	NR	SCB, 0-8
**Outcome: Psychotic symptoms (3 studies)**						
Mutuality	Ball[33]	171	24m	MD -0.1 (-0.24, 0.04; p>0.05) (delusions)	a,g,d,e	NPI, 1-12
	Ball[33]	171	24m	MD -0.04 (-0.16, 0.08; p>0.05) (hallucinations)	a,g,d,e	
Emotional distancing /boundary ambiguity	Caron[36]	60	12m	Corr 0.30 (p<0.001) (paranoia)	NR	BEHAVE-AD, 0-75
Non-adaptive coping	deVugt[46]	69	12m	MD NR (p>0.05) (psychosis)	g,s	NPI, 1-24
**Outcome: Depression (5 studies)**						
Mutuality	Ball[33]	171	24m	MD -0.43 (-0.80, 0.06; p<0.05)	a,g,d,e	Hamilton, 0-68
Quality of relationship	Burgener[20]	70	18m	Corr 0.22 (p>0.05)	NR	Cornell, 0-38
Emotional distancing /boundary ambiguity	Caron[36]	60	12m	Corr 0.01 (p>0.05)	NR	BEHAVE-AD, 0-75
Expressed emotion	Vitaliano[44]	77	15-18m	MD 2.00 (-0.31, 4.31; p>0.05)	NR	Hamilton, 0-68
Cohesion quantity	Wright[43]	14	6m	Corr -0.302 (p>0.05)	NR	Zung, 0-270
quality		14	6m	Corr -0.387 (p>0.05)	NR	
Affection quantity	Wright[43]	14	6m	Corr -2.41 (p>0.05)	NR	
quality		14	6m	Corr -0.038 (p>0.05)	NR	
Tension quantity	Wright[43]	14	6m	Corr 0.533 (p<0.01)	NR	
quality		14	6m	Corr -0.288 (p>0.05)	NR	
**Outcome: Other**						
Non-adaptive coping	deVugt[46]	69	12m	MD NR (p=0.512) (apathy)	g,s,ct	NPI, 1-48
Non-adaptive coping	deVugt[46]	69	12m	MD NR (p=0.005) (hyperactivity)	g,s,ct	NPI, 1-60
Mutuality	Morgan[34] & Kunik[32]	171	24m	MD -0.42 (-0.64, 0.20; p<0.001) HR 0.63 (0.45, 2.87; p=0.006) (aggression)	NR	CMAI, 0-156
Emotional distancing /boundary ambiguity	Caron[36]	60	6m	Corr 0.15 (p>0.05) (anxiety)	NR	BEHAVE-AD, 0-75

Legend: **OR** = odds ratio. OR>1 indicates increased risk, OR<1 indicates decreased risk. **HR** = hazard ratio, interpreted the same as OR. **PoV** = proportion of variance. This is a number between 0-1 indicating the proportion of variance in the outcome explained by the exposure. Higher numbers indicate greater explanatory power of the exposure. **Corr** = correlation. This is a number between -1 to +1 and indicates the degree to which the exposure and outcome vary together. Positive numbers indicate that exposure and outcome increase together, negative numbers indicate that the outcome increases as the exposure decreases. Larger numbers indicate stronger correlation. **MD** = mean difference in outcome. Large differences between groups suggests that the exposure might affect the outcome. **NR** = information not reported (where possible results and 95%CIs were calculated from raw data)

1. Pre-specified key confounders: **a** = age; **g** = gender; **s** = socioeconomic status; **d** = dementia severity. All other confounders: ct=carer type; e= ethnicity.

2. NPI = Neuropsychiatric Inventory; CMAI = Cohen-Mansfield Agitation Inventory; Cornell = Cornell Scale for Depression in Dementia; SCB = Screen for Caregiver Burden (selection of items); Zung = Short Zung Interviewer Assisted Depression Rating Scale;

For peer review only - http://bmjopen.bmj.com/site/about/guidelines.xhtml

Risk factors for hospitalisation, quality of life, and death (Table 5)

The outcomes of hospitalisation,[35] were examined in one study and quality of life[37,52] and death[51, 53] were each examined in two studies. . The small number of poor quality studies means it is not possible to draw conclusions regarding the association of relationship factors with these outcomes.

Table 5: Associations between relationship factors and hospitalisation, quality of life, and time to death

Risk factor	Study	N	Follow-up	Results (95% CI; p-value)	Analyses adjusted for[1]
**Hospitalisation (1 study)**					
Relationship strain	Godwin[35]	296	12m	OR 1.03 (0.92, 1.14; 0.637)	NR
**Quality of life (2 studies)**					
Quality of relationship (patient view)	Clare[37]	51	20m	MD 0.31 (p=0.06)	pqol,pd, cs, cqor
	Shroff[52]	83	NR	R^2 0.179 (p<0.001)	NR
Quality of relationship (carer view)	Clare[37]	51	20m	MD -0.13 (p=0.89)	pqol,pd, cs, cqor
**Time to death (2 studies)**					
Instrumental coping	McClendon[51]	141	5-9 years	HR 0.99 (p=0.915)	NR
Acceptance coping	McClendon[51]	141	5-9 years	HR 1.09 (p=0.644)	NR
Wishful thinking	McClendon[51]	141	5-9 years	HR 1.41 (p=0.019)	NR
	Snyder[53]	233	NR	HR 0.88 (0.673, 1.171; 0.4)	a,d,g,nc
Problem Focused coping	Snyder[53]	233	NR	HR 0.80 (0.571, 1.128; 0.2)	a,d,g,nc
Seeking Social Support	Snyder[53]	233	NR	HR 1.056 (0.787-1.416; 0.7)	a,d,g,nc
Blaming Self	Snyder[53]	233	NR	HR 0.967 (0.768-1.218; 0.7)	a,d,g,nc
Avoidance coping	Snyder[53]	233	NR	HR1.021 (0.720-1.448; 0.9)	a,d,g,nc
Blaming Others	Snyder[53]	233	NR	HR 0.867 (0.632-1.190; 0.3)	a,d,g,nc
Counting Blessings	Snyder[53]	233	NR	HR 0.648 (0.454-0.926; 0.017)	a,d,g,nc
Religiosity	Snyder[53]	233	NR	HR 0.882 (0.682-1.142; 0.341)	a,d,g,nc

Legend: OR = odds ratio. OR>1 indicates increased risk, OR<1 indicates decreased risk. HR = hazard ratio, interpreted the same as OR. MD = mean difference in outcome. Large differences between groups suggests that the exposure might affect the outcome. NR = information not reported (where possible results and 95%CIs were calculated from raw data)
1. Pre-specified key confounders:
a = age; **g** = gender; **s** = socioeconomic status; **d** = dementia severity.
All other confounders: Pqol=PWD quality of life; pd= PWD depression; cqor=carer quality of relationship; cs=carer stress; nc= non-coresidency

For peer review only

For peer review only - http://bmjopen.bmj.com/site/about/guidelines.xhtml

DISCUSSION

This systematic review assessed the evidence on the role of relationship factors on outcomes in dementia. Although it is plausible that relationship factors could increase the risk of institutionalisation, challenging behaviour and other outcomes, there is currently no robust evidence to establish which, or to what extent, elements of the caring relationship affect specific outcomes.

The majority of studies found no association between specific risk factors and the outcomes of interest. However, this is not necessarily evidence of a lack of association, as some of the studies will have been underpowered to detect differences, and most were at risk of confounding as they failed to adjust for even the most basic confounding factors. There was a suggestion of an association between factors related to the emotional withdrawal of the caregiver and subsequent increased risk of challenging behaviour in the person with dementia. All studies in this category found statistical evidence of an association. However, the methodological quality of these studies was poor. For example many did not report effect sizes while in others the effect sizes were small, suggesting that associations may not be clinically important. This could also be because the sample was too small to detect a difference. No study reported justification for their sampling or the sample sizes. There was also a potential for confounding in studies that did report effect sizes.

Amongst the strengths of this review were that we conducted a thorough, sensitive search which will have minimised the chance of missing relevant studies. By limiting inclusion to cohort studies only, we avoided interpretive difficulties from recall bias (systematic differences in how people remember risk factors) and reverse causation (when a purported risk factor is in fact a result of the outcome). The double-screening of each record by two reviewers working independently also minimised the possibility of errors and selection bias in the identification of eligible reports. All data extracted was checked in full by a second reviewer working independently.

For peer review only - http://bmjopen.bmj.com/site/about/guidelines.xhtml

A limitation of our review is that we were not able to assess for publication bias or selective reporting of results. It is known that studies with null findings are less likely to be published, and authors can selectively report their 'more interesting' findings.[54] This means it is possible there are other relevant results that have not been reported, and so would not be available to a review. We attempted to minimise the risk of publication bias by including a grey literature search.

Our ability to answer the research question was limited due to unclear and incomplete reporting, few studies assessing similar risk factors, and the potential for bias in included studies. We considered the appropriateness of using meta-analysis to produce summary estimates. However, many studies did not report full details of effect sizes and confidence intervals that are required for meta-analysis. Additionally most risk factor-outcome relationships were only reported in one or two studies (with conflicting results in the latter cases) which is insufficient for meta-analyses. It would not have been meaningful or appropriate to combine different risk factors or outcomes in pooled analysis. We assessed the potential for bias in included studies and took these assessments into consideration when reaching conclusions, thus avoiding over-interpretation of findings from studies with potentially serious problems.

Very few studies adjusted their analyses for key pre-specified confounding factors (age, gender, socioeconomic status, and dementia severity). Two thirds of included analyses did not adjust for any confounding factors. This is an important limitation as it means any findings could be explained by differences in these factors across the exposure groups – differences that are very likely in a non-randomised study where participants self-select into exposure groups. A small number of studies adjusted for other factors, but these were factors such as quality of life and emotional withdrawal that may well be on the causal pathway between the relationship factor studied and the outcome (so not a true confounder). Reporting of results was incomplete with many studies only reporting p-values, or just that an association was 'statistically significant' / 'not significant' with no effect sizes

For peer review only - http://bmjopen.bmj.com/site/about/guidelines.xhtml

or confidence intervals. This is another important limitation as in these cases we have no information about the probable magnitude of effect, and it is impossible to interpret the potential clinical implications of any statistical difference. Inadequate reporting of methods and results made it difficult to assess risk of bias. Very few studies evaluated the same risk factors, so there is also very little evidence on any individual factor. Duration of follow-up (typically 6-24 months), although theoretically long enough for an outcome to have occurred, may not have been sufficient to detect outcomes in the majority of the sample in a study. As dementia is typically a slowly progressing disease this may not be sufficient for long-term outcomes such as institutionalisation.
Finally, majority of the studies identified were published before 2000. The most recent study is from 2016, with only three studies reported in the last five years. The reasons for this apparent diminishing of literature are unclear, but the relative absence of more recent studies may have implications for applicability of the findings of this review. New long-term studies that are well conducted and fully reported are needed to reliably answer questions regarding effect of family relationship quality on institutionalisation risks.

The current evidence does not provide a basis by which GPs or other health professionals could reliably identify people at risk of poor outcome on the basis of relationship factors. However, the lack of robust evidence about the role of relationship factors does not imply that personal relationships are not important factors in dementia outcomes; many professionals working with families consider these to be important.[55 56] One plausible mechanism is that factors such as emotional withdrawal of the caregiver might prompt ‘challenging behaviour’ in the person with dementia as an attempt to elicit an emotional connection.[41] For instance, care-giver coping strategies such as constantly ‘correcting’ the person with dementia, rather than accepting their cognitive challenges, could be construed as potentially undermining the individual’s personhood or self-esteem, both of which are seen as key elements in good dementia care. In this way, any ‘challenging behaviour’ might be construed as behavioural expressions of underlying frustration or distress. It is also possible that challenging behaviours are themselves on the causal pathway

For peer review only - http://bmjopen.bmj.com/site/about/guidelines.xhtml

between relationship factors and institutionalisation – the latter plausibly becoming more likely as the caregiver becomes less able to cope with challenging behaviours. If this is the case then longer follow-up may be necessary to detect associations between relationship factors and institutionalisation. In addition, the association between quality of relationships, challenging behaviour and subsequent institutionalisation is likely to be complex. A recent study[54] found that relationship quality was one of a number of psychosocial factors associated with caregiver distress at challenging behaviour independently of the frequency of that behaviour. Similarly changes in the meaning of their relationship, and in particular the belief that their relative had lost, or would inevitably lose, their identity to dementia, is a fundamental reason why family carers experienced behaviour as challenging[55]. It may be, then, that the quality of relationship acts as a confounding variable in the association between challenging behaviour and institutionalisation.

Lack of evidence on what relationship factors predict outcomes in people with dementia should not preclude further evaluation of psychosocial interventions targeting people with dementia and their carers. Such studies could use experimental designs to identify which interventions work and in which settings. It may also be useful to look to qualitative research, exploring the views of people with dementia and their family carers on what they consider important for continued living at home, and the challenges in the relationship that they face.

CONCLUSIONS

There is currently no strong or consistent evidence on the effects of relationship factors on institutionalisation, hospitalisation, death or quality of life for people with dementia. There was a suggestion of an association between relationship factors and challenging behaviour, although the evidence for this was weak. As the current focus of dementia care is 'person-centred', which prioritises interpersonal relationships, this lack of evidence about the role of relationships in

For peer review only - http://bmjopen.bmj.com/site/about/guidelines.xhtml

dementia outcomes is striking. To improve our ability to support those with dementia and their families this evidence gap needs to be addressed.

FIGURES

Figure 1: PRISMA flow diagram

For peer review only

For peer review only - http://bmjopen.bmj.com/site/about/guidelines.xhtml

Funding statements and acknowledgements

This research is supported by the National Institute for Health Research (NIHR) Collaboration for Leadership in Applied Health Research and Care West at University Hospitals Bristol NHS Foundation Trust. The views expressed are those of the author(s) and not necessarily those of the NHS, the NIHR or the Department of Health. Authors thank Rebecca Beynon for support screening papers.

Competing interests

The authors declare that they have no competing interests.

Authors' contributions

HE was the lead researcher, contributed to the study design, carried out screening, data extraction, data analysis, and prepared the manuscript. SI carried out screening, data extraction, the risk of bias assessment, and revisions of the manuscript after the update search in Summer 2017. VL and JS contributed to the screening and data extraction. JS and PW led the study design, supervised the review and contributed to drafting the manuscript. AR developed the search strategy and conducted the searches. RC and SC conceived the original study idea, and contributed to the study design. RC provided clinical advice throughout the review and made critical revisions to the manuscript. All authors read and approved the final manuscript.

Data sharing statement

There is no additional unpublished data from this study. Any requests for access to the original data extracted from included papers should be sent to the corresponding author.

For peer review only

References

1. Fineberg NA, Haddad PM, Carpenter L, et al. The size, burden and cost of disorders of the brain in the UK. *J Psychopharm* 2013 doi: 10.1177/0269881113495118

2. Luengo-Fernandez R, Leal J, Gray AM. UK research expenditure on dementia, heart disease, stroke and cancer: are levels of spending related to disease burden? *Eur J Neurol* 2012;19(1):149-54. doi: 10.1111/j.1468-1331.2011.03500.x

3. World Health Organization. Dementia (Fact sheet no. 362). 2015. Available from: http://wwwwhoint/mediacentre/factsheets/fs362/en/ accessed 04/09/2017.

4. Prince M, Bryce R, Albanese E, et al. The global prevalence of dementia: A systematic review and metaanalysis. *Alzheimers Dement* 2013;9(1):63-75.e2. doi: 10.1016/j.jalz.2012.11.007

5. Prince M, Knapp M, Guerchet M, et al. Dementia UK Update (Second Edition): Alzheimer's Society, 2014.

6. Alzheimer's Disease International. World Alzheimer Report 2014: Dementia and Risk Reduction. An analysis of protective and modifiable factors, 2014.

7. Byrne-Davis LMT, Bennett PD, Wilcock GK. How are Quality of Life Ratings Made? Toward a Model of Quality of Life in People with Dementia. *Qual Life Res* 2006;15(5):855-65. doi: 10.1007/s11136-005-5416-9

8. Donnelly L. Care Quality Commission finds 'appalling' failings in elderly care. The Telegraph, October 2014.

9. Sawer P, Ross, T. Dozens of care homes failing the most vulnerable. The Telegraph, December 2014.

10. Nelson C. Care home concerns: YouGov UK; 6 March 2013. Available from: https://yougov.co.uk/news/2013/03/06/care-home-concerns/ accessed 04/09/2017.

11. Department of Health. Prime Minister's challenge on dementia 2020. Department of Health; February 2015. Available from:

For peer review only

https://www.gov.uk/government/uploads/system/uploads/attachment_data/file/414344/pm-dementia2020.pdf accessed 04/09/2017.

12. Tschanz JT, Piercy K, Corcoran CD, et al. Caregiver Coping Strategies Predict Cognitive and Functional Decline in Dementia: The Cache County Dementia Progression Study. *Am J Geriatr Psychiatry* 2013;21(1):57-66. doi: 10.1016/j.jagp.2012.10.005

13. García-Alberca JM, Cruz B, Lara JP, et al. The experience of caregiving: the influence of coping strategies on behavioral and psychological symptoms in patients with Alzheimer's disease. *Aging Ment Health* 2013;17(5):615-22. doi: 10.1080/13607863.2013.765833

14. Luppa M, Riedel-Heller SG, Stein J, et al. Predictors of Institutionalisation in Incident Dementia – Results of the German Study on Ageing, Cognition and Dementia in Primary Care Patients (AgeCoDe Study). *Dement Geriatr Cogn Disord* 2012;33(4):282-88.

15. Fauth E, Hess K, Piercy K, et al. Caregivers' relationship closeness with the person with dementia predicts both positive and negative outcomes for caregivers' physical health and psychological well-being. *Aging Ment Health* 2012;16(6):699-711. doi: 10.1080/13607863.2012.678482

16. Quinn C, Clare L, Woods B. The impact of the quality of relationship on the experiences and wellbeing of caregivers of people with dementia: A systematic review. *Aging Ment Health* 2009;13(2):143-54. doi: 10.1080/13607860802459799

17. Norton MC, Piercy KW, Rabins PV, et al. Caregiver–Recipient Closeness and Symptom Progression in Alzheimer Disease. The Cache County Dementia Progression Study. *J Gerontol B Psychol Sci Soc Sci* 2009;64B(5):560-68. doi: 10.1093/geronb/gbp052

18. Spitznagel MB, Tremont G, Davis JD, et al. Psychosocial Predictors of Dementia Caregiver Desire to Institutionalize: Caregiver, Care Recipient, and Family Relationship Factors. *J Geriatr Psychiatry Neurol* 2006;19(1):16-20. doi: 10.1177/0891988705284713

19. Banerjee S, Murray J, Foley B, et al. Predictors of institutionalisation in people with dementia. *J Neurol Neurosurg Psychiatry* 2003;74(9):1315-16. doi: 10.1136/jnnp.74.9.1315

For peer review only - http://bmjopen.bmj.com/site/about/guidelines.xhtml

20. Burgener S, Twigg P. Relationships among caregiver factors and quality of life in care recipients with irreversible dementia. *Alzheimer Dis Assoc Disord* 2002;16(2):88-102.

21. Bergmann K, Manchee V, Woods RT. Effect of family relationships on psychogeriatric patients. *J R Soc Med* 1984;77(10):840-44.

22. Benbow SM, Sharman V. Review of family therapy and dementia: twenty-five years on. *Int Psychogeriatr* 2014;26(Special Issue 12):2037-50.

23. Cheston R, Ivanecka A. Individual and group psychotherapy with people diagnosed with dementia: a systematic review of the literature. *Int J Geriatr Psychiatry* 2016:n/a-n/a. doi: 10.1002/gps.4529

24. Edwards HB, Savović J, Whiting P, et al. Quality of relationships as predictors of outcomes in people with dementia: a systematic review protocol. *BMJ Open* 2016;6(4) doi: 10.1136/bmjopen-2015-010835

25. Reynolds J, Houlson C, Coleman L. Understanding relationship quality London: One plus One; 2014. Available from: http://www.oneplusone.org.uk/wp-content/uploads/2014/02/UnderstandingRelationship-Quality-by-Jenny-Reynolds-Dr-Catherine-Houlston-and-Dr-Lester-Coleman.pdf accessed 04/09/2017.

26. Hazan C, Shaver P. Romantic love conceptualized as an attachment process. *J Pers Soc Psychol* 1987;52(3):511-24. doi: 10.1037/0022-3514.52.3.511

27. Hazan C, Shaver PR. Attachment as an Organizational Framework for Research on Close Relationships. *Psychol Inq* 1994;5(1):1-22.

28. Kuipers L, Bebbington P. Expressed emotion research in schizophrenia: theoretical and clinical implications. *Psychol Med* 1988;18(4):893-909. doi: 10.1017/S0033291700009831

29. Raune D, Kuipers E, Bebbington PE. Expressed emotion at first-episode psychosis: investigating a carer appraisal model. *Br J Psychiatry* 2004;184(4):321-26. doi: 10.1192/bjp.184.4.321

For peer review only

For peer review only - http://bmjopen.bmj.com/site/about/guidelines.xhtml

30. Wells GA, Shea B, O'Connell D, et al. The Newcastle-Ottawa Scale (NOS) for assessing the quality of nonrandomised studies in meta-analyses. 2014. Available from: http://www.ohri.ca/programs/clinical_epidemiology/oxford.asp accessed 04/09/2017.

31. Jüni P, Witschi A, Bloch R, et al. The hazards of scoring the quality of clinical trials for meta-analysis. *JAMA* 1999;282(11):1054-60. doi: 10.1001/jama.282.11.1054

32. Kunik ME, Snow AL, Davila JA, et al. Causes of aggressive behavior in patients with dementia. *J Clin Psychiatry* 2010;71(9):1145-52. doi: 10.4088/JCP.08m04703oli

33. Ball V, Snow AL, Steele AB, et al. Quality of Relationships as a Predictor of Psychosocial Functioning in Patients With Dementia. *J Geriatr Psychiatry Neurol* 2010;23(2):109-14. doi: 10.1177/0891988710363709

34. Morgan RO, Sail KR, Snow AL, et al. Modeling Causes of Aggressive Behavior in Patients With Dementia. *Gerontologist* 2013;53(5):738-47. doi: 10.1093/geront/gns129

35. Godwin KM, Morgan RO, Walder A, et al. Predictors of Inpatient Utilization among Veterans with Dementia. *Curr Gerontol Geriatr Res* 2014;2014:10. doi: 10.1155/2014/861613

36. Caron W, Boss P, Mortimer J. Family boundary ambiguity predicts Alzheimer's outcomes. *Psychiatry* 1999;62(4):347-56.

37. Clare L, Woods RT, Nelis SM, et al. Trajectories of quality of life in early-stage dementia: individual variations and predictors of change. *Int J Geriatr Psychiatry* 2014;29(6):616-23. doi: 10.1002/gps.4044

38. Spruytte N, Van Audenhove C, Lammertyn F. Predictors of institutionalization of cognitively-impaired elderly cared for by their relatives. *Int J Geriatr Psychiatry* 2001;16(12):1119-28. doi: 10.1002/gps.484

39. Fisher L, Lieberman MA. A Longitudinal Study of Predictors of Nursing Home Placement for Patients With Dementia: The Contribution of Family Characteristics. *Gerontologist* 1999;39(6):677-86. doi: 10.1093/geront/39.6.677

For peer review only

For peer review only - http://bmjopen.bmj.com/site/about/guidelines.xhtml

40. Stevens A, Owen J, Roth D, et al. Predictors of time to nursing home placement in White and African American individuals with dementia. *J Aging Health* 2004;16(3):375-97.

41. Perren S, Schmid R, Herrmann S, et al. The impact of attachment on dementia-related problem behavior and spousal caregivers' well-being. *Attach Hum Dev* 2007;9(2):163-78. doi: 10.1080/14616730701349630

42. Wright LK. Alzheimer's disease afflicted spouses who remain at home: can human dialectics explain the findings? *Soc Sci Med* 1994;38(8):1037-46.

43. Wright LK, Hickey JV, Buckwalter KC, et al. Spousal interactions in Alzheimer's disease and stroke caregiving: Relationship to care recipients' functional abilities and physical and emotional health. *J Am Psychiatr Nurses Assoc* 1998;4(6):169-81. doi: 10.1016/S1078-3903%2898%2990028-3

44. Vitaliano PP, Young HM, Russo J, et al. Does Expressed Emotion in Spouses Predict Subsequent Problems Among Care Recipients With Alzheimer's Disease? *J Gerontol* 1993;48(4):P202-P09. doi: 10.1093/geronj/48.4.P202

45. Bannister C, Ballard C, Lana M, et al. Placement of dementia sufferers in residential and nursing home care. *Age Ageing* 1998;27(2):189-93.

46. de Vugt ME, Stevens F, Aalten P, et al. Do caregiver management strategies influence patient behaviour in dementia? *Int J Geriatr Psychiatry* 2004;19(1):85-92. doi: 10.1002/gps.1044

47. Wells Y, Over R. Institutional Placement of a Dementing Spouse: The Influence of Appraisal, Coping Strategies, and Social Support. *Aust J Psychol* 1998;50(2):100-05. doi: 10.1080/00049539808257540

48. Torossian CL, Ruffins S. Relationship Between Family Dynamics of Caregivers, Depression, and the Likelihood of Institutionalization of Alzheimer's Patients. *J Contemp Psychother* 1999;29(2):127-42. doi: 10.1023/a:1021956715387

49. Markiewicz D, Reis M, Gold DP. An exploration of attachment styles and personality traits in caregiving for dementia patients. *Int J Aging Hum Dev* 1997;45(2):111-32.

For peer review only

50. Pruchno RA, Michaels JE, Potashnik SL. Predictors of Institutionalization Among Alzheimer Disease Victims With Caregiving Spouses. *J Gerontol* 1990;45(6):S259-S66. doi: 10.1093/geronj/45.6.S259

51. McClendon MJ, Smyth KA, Neundorfer MM. Survival of Persons With Alzheimer's Disease: Caregiver Coping Matters. *Gerontologist* 2004;44(4):508-19. doi: 10.1093/geront/44.4.508

52. Shroff HB. Family resiliency, sense of coherence, social support and psychosocial interventions: Reducing caregiver burden and determining the quality of life in persons with alzheimer's disease [Doctor of Philosophy]. Florida Atlantic University, 2014.

53. Snyder CM. Dementia caregiver personality traits and coping strategies: Association with care recipient outcomes [Doctor of Philosophy]. Utah State University, 2015.

54. Dwan K, Gamble C, Williamson PR, et al. Systematic Review of the Empirical Evidence of Study Publication Bias and Outcome Reporting Bias — An Updated Review. *PLoS One* 2013;8(7):e66844. doi: 10.1371/journal.pone.0066844

55. Kitwood T. Dementia Reconsidered: The Person Comes First Buckingham: Open University Press 1997.

56. Cheston R, Bender M. Understanding Dementia: The Man with the Worried Eyes. London: Jessica Kingsley Press 1999.

For peer review only

For peer review only - http://bmjopen.bmj.com/site/about/guidelines.xhtml

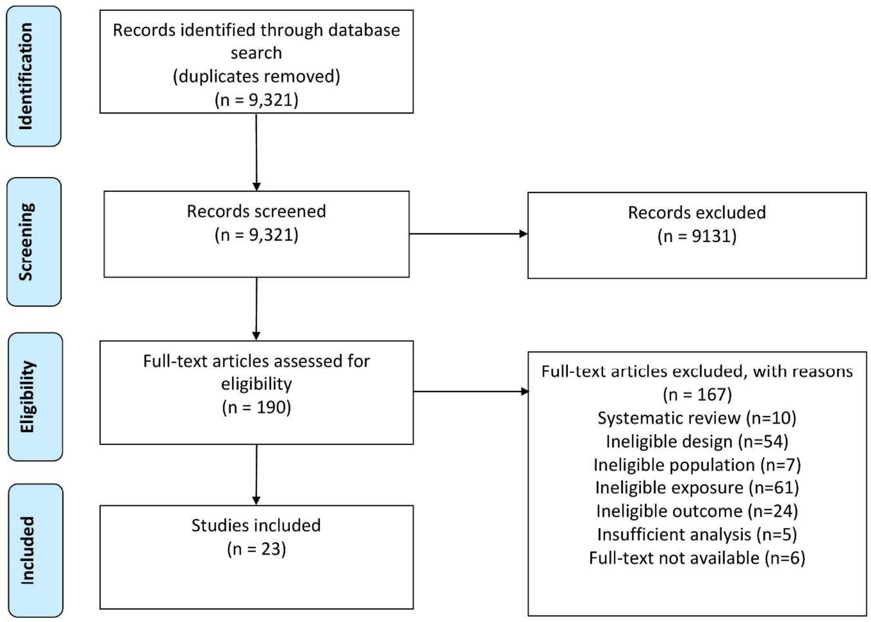

Figure 1. PRISMA flow diagram

254x190mm (300 x 300 DPI)

PRISMA 2009 Checklist

Section/topic	#	Checklist item	Reported on page #
**TITLE**			
Title	1	Identify the report as a systematic review, meta-analysis, or both.	1
**ABSTRACT**			
Structured summary	2	Provide a structured summary including, as applicable: background; objectives; data sources; study eligibility criteria, participants, and interventions; study appraisal and synthesis methods; results; limitations; conclusions and implications of key findings; systematic review registration number.	2
**INTRODUCTION**			
Rationale	3	Describe the rationale for the review in the context of what is already known.	3-4
Objectives	4	Provide an explicit statement of questions being addressed with reference to participants, interventions, comparisons, outcomes, and study design (PICOS).	4
**METHODS**			
Protocol and registration	5	Indicate if a review protocol exists, if and where it can be accessed (e.g., Web address), and, if available, provide registration information including registration number.	4
Eligibility criteria	6	Specify study characteristics (e.g., PICOS, length of follow-up) and report characteristics (e.g., years considered, language, publication status) used as criteria for eligibility, giving rationale.	4-6
Information sources	7	Describe all information sources (e.g., databases with dates of coverage, contact with study authors to identify additional studies) in the search and date last searched.	6
Search	8	Present full electronic search strategy for at least one database, including any limits used, such that it could be repeated.	6
Study selection	9	State the process for selecting studies (i.e., screening, eligibility, included in systematic review, and, if applicable, included in the meta-analysis).	6
Data collection process	10	Describe method of data extraction from reports (e.g., piloted forms, independently, in duplicate) and any processes for obtaining and confirming data from investigators.	7
Data items	11	List and define all variables for which data were sought (e.g., PICOS, funding sources) and any assumptions and simplifications made.	6-7
Risk of bias in individual studies	12	Describe methods used for assessing risk of bias of individual studies (including specification of whether this was done at the study or outcome level), and how this information is to be used in any data synthesis.	7-8
Summary measures	13	State the principal summary measures (e.g., risk ratio, difference in means).	6
Synthesis of results	14	Describe the methods of handling data and combining results of studies, if done, including measures of consistency (e.g., I^2) for each meta-analysis.	6

For peer review only - http://bmjopen.bmj.com/site/about/guidelines.xhtml

PRISMA 2009 Checklist

Section/topic	#	Checklist item	Reported on page #
Risk of bias across studies	15	Specify any assessment of risk of bias that may affect the cumulative evidence (e.g., publication bias, selective reporting within studies).	7
Additional analyses	16	Describe methods of additional analyses (e.g., sensitivity or subgroup analyses, meta-regression), if done, indicating which were pre-specified.	7
**RESULTS**			
Study selection	17	Give numbers of studies screened, assessed for eligibility, and included in the review, with reasons for exclusions at each stage, ideally with a flow diagram.	8
Study characteristics	18	For each study, present characteristics for which data were extracted (e.g., study size, PICOS, follow-up period) and provide the citations.	Table 1
Risk of bias within studies	19	Present data on risk of bias of each study and, if available, any outcome level assessment (see item 12).	Table 2
Results of individual studies	20	For all outcomes considered (benefits or harms), present, for each study: (a) simple summary data for each intervention group (b) effect estimates and confidence intervals, ideally with a forest plot.	Tables 3-5
Synthesis of results	21	Present the main results of the review. If meta-analyses are done, include for each, confidence intervals and measures of consistency	Tables 3-5 plus page 11,13,15
Risk of bias across studies	22	Present results of any assessment of risk of bias across studies (see Item 15).	Not done (see p20)
Additional analysis	23	Give results of additional analyses, if done (e.g., sensitivity or subgroup analyses, meta-regression [see Item 16]).	n/a
**DISCUSSION**			
Summary of evidence	24	Summarize the main findings including the strength of evidence for each main outcome; consider their relevance to key groups (e.g., healthcare providers, users, and policy makers).	20
Limitations	25	Discuss limitations at study and outcome level (e.g., risk of bias), and at review-level (e.g., incomplete retrieval of identified research, reporting bias).	21-22
Conclusions	26	Provide a general interpretation of the results in the context of other evidence, and implications for future research.	23
**FUNDING**			
Funding	27	Describe sources of funding for the systematic review and other support (e.g., supply of data); role of funders for the systematic review.	26

For peer review only - http://bmjopen.bmj.com/site/about/guidelines.xhtml

PRISMA 2009 Checklist

From: Moher D, Liberati A, Tetzlaff J, Altman DG, The PRISMA Group (2009). Preferred Reporting Items for Systematic Reviews and Meta-Analyses: The PRISMA Statement. PLoS Med 6(7): e1000097. doi:10.1371/journal.pmed1000097

For more information, visit: **www.prisma-statement.org**.

Page 2 of 2

For peer review only

BMJ Open

Quality of family relationships and outcomes of dementia: a systematic review

Journal:	*BMJ Open*
Manuscript ID	bmjopen-2016-015538.R2
Article Type:	Research
Date Submitted by the Author:	02-Oct-2017
Complete List of Authors:	Edwards, Hannah; University of Bristol, School of Social and Community Medicine<br>Ijaz , Sharea ; NIHR CLAHRC West<br>Whiting, Penny; University of Bristol, School of Social and Community Medicine<br>Leach, Verity; University of Bristol<br>Richards, Alison; University of Bristol, School of Social and Community Medicine<br>Cullum, Sarah; University of Bristol, School of Social and Community Medicine<br>Cheston, Richard; University of the West of England, Health and Social Sciences<br>Savovic, Jelena; University of Bristol, NIHR CLAHRC West
**Primary Subject Heading**:	Neurology
Secondary Subject Heading:	Evidence based practice, Health services research, Patient-centred medicine, Epidemiology, Mental health
Keywords:	Dementia < NEUROLOGY, family relationships, challenging behaviour, institutionalisation, systematic review

SCHOLARONE™ Manuscripts

Title

Quality of family relationships and outcomes of dementia: a systematic review

Author details

Corresponding author: Jelena Savović, Bristol Medical School, University of Bristol, and National Institute for Health Research (NIHR) Collaboration for Leadership in Applied Health Research and Care (CLAHRC) West.

Email: j.savovic@bristol.ac.uk

Telephone: 0117 342 1243

Address: 9th Floor Whitefriars, Lewins Mead, Bristol BS1 2NT, UK

Full author list: *Hannah B Edwards[1,2], *Sharea Ijaz[1,2], Penny Whiting[1,2], Verity Leach[1,2], Alison Richards[1,2], Sarah Cullum[3], Richard Cheston[4], Jelena Savović[1,2]

1. Bristol Medical School, University of Bristol. Bristol, UK
2. National Institute for Health Research (NIHR) Collaboration for Leadership in Applied Health Research and Care (CLAHRC) West. Bristol, UK
3. University of Auckland. Auckland, New Zealand.
4. Health and Social Sciences, University of the West of England. Bristol, UK

* Joint first authors

Keywords: Dementia, carers, family relationships, institutionalisation, challenging behaviour

Word count: 3746

Tables: 5

Figures: 1

For peer review only - http://bmjopen.bmj.com/site/about/guidelines.xhtml

ABSTRACT

Objectives: Evaluation of the association between the quality of relationship between a person with dementia and their family carer and outcomes for the person with dementia.

Design: Systematic review.

Eligibility criteria: Cohort studies of people with clinically diagnosed dementia and their main carers. Exposures of interest were any elements of relationship quality, e.g. attachment style, expressed emotion, coping style. Our primary outcome was institutionalisation, and secondary outcomes were hospitalisation, death, quality of life, and behavioural and psychiatric symptoms of dementia ('challenging behaviour').

Data sources: Medline, Embase, Web of Science, PsycInfo, the Cochrane Library and Opengrey were searched from inception to May 2017.

Study appraisal and synthesis methods: The Newcastle-Ottawa Scale was used to assess risk of bias. A narrative synthesis of results was performed due to differences between studies.

Results: Twenty studies were included. None of the studies controlled for all pre-specified confounding factors (age, gender, socio-economic status, and severity of dementia). Reporting of results was inadequate with many studies simply reporting whether or not associations were 'statistically significant' without providing effect size estimates or confidence intervals. There was a suggestion of an association between relationship factors and global challenging behaviour. All studies evaluating global challenging behaviour provided statistical evidence of an association (most p-values below 0.02). There was no consistent evidence for an association for any other outcome assessed.

Conclusions: There is currently no strong or consistent evidence on the effects of relationship factors on institutionalisation, hospitalisation, death or quality of life for people with dementia. There was a suggestion of an association between relationship factors and challenging behaviour, although the evidence for this was weak. To improve our ability to support those with dementia and their families further robust studies are needed.

For peer review only - http://bmjopen.bmj.com/site/about/guidelines.xhtml

Systematic review registration: CRD42015020518

ARTICLE SUMMARY

Strengths and limitations of this study

- Broad search strategy so unlikely to have missed relevant studies
- Double screening minimises selection bias
- We were not able to assess publication bias and the potential for selective reporting of outcomes within studies.

BACKGROUND

Dementia is a key public health concern in the UK[1-3] with around 7% of all those over 65 affected, and the numbers of people with dementia predicted to double every 20 years.[4-6] Institutionalisation (being placed in a full time care /nursing home) is a key outcome for people with dementia, their families, and the healthcare system. Although in many circumstances institutionalisation may be the best or only option for the person affected by dementia, most people report that they would prefer to stay living in their own home.[7] Recent media attention to a few very poorly run care homes has also lead to concerns about institutionalisation.[8-10] Additionally, the financial cost of full-time care is very high, both for affected individuals and their families, and for the public, as public taxes are used to contribute to care home fees. Consequently, for some time it has been UK government policy to help families to continue supporting people with dementia at home, specifically to delay or avoid institutionalisation.[11]

The quality of relationship between the person with dementia and people who care for them has been linked with a range of outcomes including institutionalisation, cognitive and functional decline, and quality of life.[12-21] There is also growing interest in the potential for psychosocial interventions to improve outcomes, by enhancing interactions within families.[22 23] If families are better equipped to

For peer review only - http://bmjopen.bmj.com/site/about/guidelines.xhtml

cope with the psychological and emotional challenges of dementia, then care at home may be sustained for longer. However, it is not clear which elements of the relationship are predictive of early institutionalisation or which lead to a faster decline. This evidence is necessary both to justify and to help to develop early psychosocial interventions, ideally at the point of diagnosis.

To address this issue we performed a systematic review of the evidence on how elements of relationship quality between the person with dementia and their main informal (family) carer are associated with outcomes for the person with dementia.

METHODS

This study was a systematic review, registered with the PROSPERO International Prospective Register of systematic reviews, registration number CRD42015020518. The full protocol has been published.[24]

Eligibility criteria

Only cohort studies (prospective and retrospective) were included in the review. Relevant systematic reviews were obtained and used as a means of identifying other original studies.[24] Qualitative, case control (unless nested in a prospective cohort), and cross-sectional studies were excluded.

The population of interest was people with dementia and their main informal caregiver (most commonly a spouse or child). Professional, paid caregivers were excluded. People with all types of clinically-diagnosed dementia were included.

The exposures of interest were factors that capture an element of relationship quality. We adopted a broad definition of “relationship quality” as how happy or satisfied an individual is in his or her relationship. [25] Attachment style, coping style, affection, and expressed emotion were all identified

For peer review only - http://bmjopen.bmj.com/site/about/guidelines.xhtml

at the design stage as key exposures of interest. While affection is a relatively straightforward term, attachment style, coping and expressed emotion relate to specific psychological constructs. 'Attachment style' is a term originally developed to understand the emotional relationship between children and parents, but has since been extended to adult romantic relationships. Four main styles of attachment have been identified in adults: secure; anxious–preoccupied; dismissive-avoidant; and fearful-avoidant.[26 27] Coping is a wide-ranging construct that includes elements which are clearly measures of relationship quality (e.g. 'relationship-focussed coping') and those which are more individual in nature (e.g. acceptance coping). However, as even individual coping styles are typically initiated in response to aspects of relationships, we felt that this was an appropriate exposure to capture. 'Expressed Emotion' (EE) is a measure of the family environment based on how the relatives of a psychiatric patient spontaneously talk about/to the patient. High levels of expressed emotion have been associated with worse prognosis in a number of mental illnesses including schizophrenia. This may be due to emotional over-involvement, which can be experienced as hostility, criticism and intolerance.[28 29]

Two other notable factors emerged as key exposures when reviewing the literature: 'mutuality' and 'boundary ambiguity'. Mutuality is a cluster concept capturing levels of positive engaging interaction, attachment and emotional support. Boundary ambiguity involves uncertainty about whether a person is in or out of the family group. This occurs as a result of significant changes in that person including those cognitive, functional, mood and personality changes that are indicative of dementia. Boundary ambiguity is associated with, and taken as an indicator of emotional distancing and the withdrawal of the caregiver from the person with dementia. Other factors emerging from the literature were included if they captured an element of relationship quality, and this was assessed on a case-by-case basis through discussion with the study team. Carer abuse was excluded, as this was considered to be a different area of research. Overall measures of carer burden

For peer review only - http://bmjopen.bmj.com/site/about/guidelines.xhtml

were also excluded as an exposure because they relate more to cognitive and functional levels in dementia than to relationship quality.

The primary outcome of interest was institutionalisation. This is a key event in the course of dementia, both socially and financially, from the perspectives of the individual, their family and the public. Secondary outcomes were hospitalisations, death (or time to death), quality of life (QoL), and challenging behaviour (also referred to as the behavioural and psychological symptoms of dementia or BPSD). Examples of challenging behaviour can include depression, anxiety, aggression, paranoia, hallucinations and delusions. Studies measuring QoL or challenging behaviour as the outcome had to use validated assessment tools to be included in the review.

Search strategy

Medline, Embase, Web of Science, PsycInfo, The Cochrane Library and Opengrey were searched from inception to May 2017 without any language restrictions. The full search strategy is available in the supplementary data of our published protocol.[24] All results were imported into an Endnote X7 reference library, and into a bespoke-built Microsoft Access 2013 database to manage screening.

Selection of papers

The titles and abstracts of all identified papers were screened in duplicate by two reviewers working independently, and all potentially relevant papers were retrieved. All retrieved papers were read in full and assessed for eligibility using a standardised and piloted inclusion checklist, applied by two reviewers independently. Any discrepancies between the reviewers (at either stage of screening) were resolved through discussion.

For peer review only - http://bmjopen.bmj.com/site/about/guidelines.xhtml

Data extraction

Data were extracted from included studies using a bespoke data collection form, which was piloted on six studies and amended as a result of the piloting. Data extracted included study characteristics, characteristics of the population of people with dementia and their carers, recruitment and response, the exposures and outcomes and how and when they were measured, and details of the analyses and results. The terminology for risk factors and outcomes was used as reported by the original study authors. Where numerical results were incompletely reported, where possible, relevant results (effect estimates, standard errors, 95% confidence intervals) were calculated from the raw data. Multiple publications of the same dataset were counted as a single study. In the case where analyses of the same association was repeated in more than one report, our policy was either to include the result based on the largest sample size only, or if sample sizes were equivalent, then we would include the result from the most recent publication only. Data were extracted from the published reports only, we did not contact authors for additional unpublished information.

Analysis

We planned to use meta-analysis to estimate summary effect sizes if there had been sufficient studies with similar populations, exposures and outcomes. As meta-analysis was not possible, a narrative synthesis of results is provided.[24]

Assessment of methodological quality and risk of bias

The Newcastle-Ottawa Scale[30] (NOS) was used to assess risk of bias for included reports. This is an eight-item questionnaire that assesses the following methodological criteria: representativeness of the exposed cohort; selection of the non-exposed cohort; ascertainment of exposure; demonstration that the outcome of interest was not present at the start of the study; comparability of cohorts (risk of confounding); assessment of exposure and outcome; and length and adequacy of follow up. NOS allocates ‘stars’ for adequate methods, but does not specifically advise calculating

For peer review only - http://bmjopen.bmj.com/site/about/guidelines.xhtml

For peer review only

the sum of allocated stars to give an overall score.[30] Empirical evidence also suggests that numerical quality scores are not helpful in differentiating between studies of high and low risk of bias.[31] For this reason we considered each of the eight criteria of the NOS tool separately and assessed the study as having adequate methods for that particular aspect of study conduct if the 'star' could be allocated for that NOS criterion. In our protocol we considered ten factors as potentially important confounding domains.[24] During the piloting of the data-extraction and risk of bias assessment the team agreed a minimum number of essential confounders that all studies should have adjusted for. A study had to control for the following four pre-specified factors to be at low risk of confounding: age, gender, socio-economic status (SES) and dementia severity. We also recorded other confounders studies have adjusted for, in addition to the four main confounders used for risk of bias assessment.

RESULTS

The search identified 9,321 potentially relevant papers. Of these, 190 papers were retrieved for full-text screening, 23 publications [20 32-53] met the eligibility criteria (Figure 1). Four of the 23 publications were based on data from one cohort study[32-35] and so the total number of unique studies was 20 (2340 participants with dementia). As the four connected publications each contributed unique results, all were included in the review.

Study characteristics

The majority of included unique studies (14 out of 20) came from the US, and there was one each from the UK, Canada, Switzerland, Belgium, the Netherlands, and Australia. Sample sizes in the relevant analyses ranged from 29 to 220 (mean 143). Years of publication ranged from 1990 to 2016, with most from the 1990s and early 2000s. The most frequently reported dementia type was Alzheimer's disease, although in eight studies the distribution of dementia types was not reported. Time since diagnosis ranged from 3 months to 6.5 years, although in six studies this was not reported. The majority (13/20) did not report participants' ethnicity, where this was reported,

For peer review only - http://bmjopen.bmj.com/site/about/guidelines.xhtml

cohorts were predominantly Caucasian. The majority of caregivers included were spouses (100% in seven studies, and more than 50% in another seven). Other carers were children of the person with dementia. The characteristics of included studies are presented in Table 1.

For peer review only

For peer review only - http://bmjopen.bmj.com/site/about/guidelines.xhtml

Table 1. Characteristics of included studies

Study ID	Country	People with dementia						Carers					Exposures*	Outcomes*
		N	% female	dementia type	Dementia duration years (SD); range	Dementia severity Mean (SD); range	Age years (SD); range	N	% female	% spouse	% child	Age years (SD); range		
Caron 1999[36]	USA	60	22	AD	3.4 (2.5);1-13	NR	68.26 (8.44); 53-90	60	79	86	8	63.57 (8.94; 30-84	Boundary ambiguity (caregiver closeout)	BPSD – depression; anxiety; paranoia; activity disturbance
Clare 2013 [37]	UK	51	50.98	61% AD, 21% VD, 17% Mixed	NR	MMSE= 24.5 (2.80); 18-30	76.75 (7.88); 55-91	51	70	57	31	65.06 (14.50); 33-89	positive quality of relationship: Positive Affect Index	QoL Alzheimer's Disease Scale (QoL-AD)
Kunik 2010 (4 papers) [32-35]	USA	215	4.6	NR	≤ 1	NR	76 (6.2)	215	NR	NR	NR	NR	Mutuality, relationship strain	BPSD – aggression, depression, psychosis, hospitalisation
Spruytte 2001[38]	Belgium	144	68.8	NR	≥ 0.25	GDS mean= 6	82; 61-94	144	69.4	38.9	50.7	63; 38-90	Quality of relationship; criticism	Institutionalisation
Fisher 1999[39]	USA	164	0.44	NR	6.5	MMSE= 17 (6.25)	77 (7)	164	54% non NHP, 47% NHP	0	100	45 (9)	Emotional Closeness; Boundary ambiguity; Negative family feelings	Institutionalisation
Burgener 2002 [20]	USA	96	57	NR	≤ 1	MMSE= 21.52; 10-29	77.3 (7.8); 55-96	96	74	56	40	63.7 (12.2); 35-83	Quality of relationship	Institutionalisation
Stevens 2004 [40]	USA	215	71.2	NR	NR	MMSE= 12.2 (8.7)	74.4 (8.2)	215	NR	47.9	40.5	NR	Coping strategies	Institutionalisation

For peer review only

For peer review only - http://bmjopen.bmj.com/site/about/guidelines.xhtml

Perren 2007 [41]	Switzerland	68	NR	60% AD, 25% VD, 15% other	NR	MMSE = 21.6 (5.1)	74.8 (7.6)	68	NR	100	0	70.9 (10.0)	attachment style	Overall care recipients problem behaviour
Wright 1994[42]	USA	29		AD	4.8; 1-11	MMSE = 18; 4-22, GDS = 4.4; 2-6	67.5; 51-83	30	80	100	0	67.4; 51-81	Cohesion; Tension; affection; present/past marital happiness	Institutionalisation
Wright 1998[43]	USA	14	50	AD	2.8	MMSE of 21.29 (3.69); 16-26	65; 49-85	14	50	100	0	NR	Cohesion; tension; affection	BPSD - Depression
Vitaliano 1993[44]	USA	77	32	AD	4.3 (2.1)	NR	70.9 (6.9)	77	NR	100	0	67.2 (7.4)	Expressed Emotion	Depression; negative behaviour
Bannister 1998[45]	UK	116	72.4	72% probable AD, 17% VD, 10% LB, 3% unclear	NR	CAMCOG mean= 45.2	79.8	116	59.5	14.7	NR	65	Coping strategies	Institutionalisation
de Vugt 2004 [46]	The Netherlands	99	56.6	74% AD, 19% VD, 2% frontal lobe, 3% Parkinson's, 2% mixed	3.5 (31.1)	MMSE= 18.2 (4.8)	78.2 (8.4)	99	66.6	55.6	44.4	61.9 (11.9)	Coping strategies	psychosis; mood/apathy; hyperactivity; overall BPSD
Wells 1998[47]	Australia	93	42	NR	5.6 (4.2)	NR	76.1 (7.3) men; 74.6 (6.2) women	93	58	100	0	74.7 (6.2) men; 71.4 (7.9) women	Coping strategies	Institutionalisation
Torossian 1999 [48]	USA	197	60.9	AD	NR	GDS range= 4-7	NR	197	39.1	100	0	70.7	adaptability; cohesion	institutionalization

For peer review only

For peer review only - http://bmjopen.bmj.com/site/about/guidelines.xhtml

Markiewicz 1997[49]	Canada	108	56.6	92% AD, 8% dementia + stroke	3.43 (2.57)	MMSE= 12.86 (7.95)	74.42 (8.37)	108	68.1	65.5	34.5	62.24 (12.97)	attachment styles	institutionalisation
Pruchno 1990[50]	USA	220	NR	AD	NR	NR	NR	220	67.9	100	0	70.2; 45-94	Quality of relationship	Institutionalisation
McClendon 2004 [51]	USA	141	45	71.5% probable AD, 28.5% possible AD	4.14 (2.47)	MMSE= 17.28 (6.50)	72.46 (7.91)	141	NR	75	NR	NR	Caregiver coping	Time to death
Shroff 2015[52]	USA	NR	NR	NR	NR	NR	84.78 (7.78)	83	85.5	54.3	NR	62.38 (18.21)	family coping and coherence	PwD Qol
Snyder 2016[53]	USA	233	56.7	NR	3.49 (1.80)	NR	86.08 (5.83)	233	78.5	37.3	53.6	66.27 (13.21)	carer coping strategies	incidence of severe dementia, time to institutionalisation, mortality

AD= Alzheimer's dementia; BPSD= Behavioural and Psychological Symptoms of Dementia; CAMCOG= Cambridge Cognition score; CBA= controlled before and after study; DRS= Dementia Rating Scale; GDS= Global Deterioration Scale; MMSE= mini mental state examination; N= numbers in analysis; NR= Not reported; SD= standard deviation; PwD= person with dementia; QoL= quality of life; RCT= randomised controlled trial; NHP= nursing home placement; VD= vascular dementia

*The exposures and outcomes listed in table refer only to the ones relevant to our review question

Study design: All studies were prospective cohorts except for Godwin 2014 (randomized trial) and Scroff 2015 (controlled before & after intervention study) - both were analysed as prospective cohorts disregarding intervention status as the focus of the review were associations of relationship risk factors rather than interventions effectiveness.

For peer review only - http://bmjopen.bmj.com/site/about/guidelines.xhtml

Risk of bias in included studies (Table 2)

None of the studies met all eight NOS criteria. All studies had adequate ascertainment of exposure as these were all based on structured interviews. All but one also had adequate 'demonstration that the outcome of interest was not present at start of study' and 'follow up long enough for outcome to occur'. Blind independent outcome assessment was not possible in many studies with institutionalisation as the outcome because this outcome tended to be self-reported by the carer.

In total, 60 separate analyses were included in this review, of which 40 (two thirds) did not control for any potential confounding factors. In the 20 analyses that did include some adjustment, only a minority adjusted for any of the factors we identified as key potential confounders. None of the studies adjusted for all four key confounding factors. Three studies each adjusted for three out of the four key confounding factors.[33 50 53] A further two studies adjusted for two of the four identified key confounders.[46 47]

Seven studies had inadequate follow up of the cohort (loss to follow up). [37 47 48 50-53] Many studies presented insufficient information to make a clear judgment on some of the NOS criteria. In addition to potential risk of bias, many studies had reporting problems. Of the 60 included results, eight (13%) neither reported effect size estimates nor confidence intervals, 40 (67%) reported an effect size with no confidence intervals, and 23 (38%) did not report specific p-values. Only six analyses (10%) fully reported their results with effect size, confidence intervals, and specific p-values.

For peer review only - http://bmjopen.bmj.com/site/about/guidelines.xhtml

Table 2. Risk of bias assessment based on Newcastle –Ottawa scale

Study Author Year	Country	Newcastle-Ottawa Scale items addressing different methodological components of the study and potential sources of bias							
		Representativeness of the exposed cohort	Selection of the non-exposed cohort	Ascertainment of exposure	Outcome of interest was not present at start of study	Comparability of cohorts on the basis of the design/analysis (confounding)	Assessment of outcome blind/ record linkage	Follow-up long enough for outcome to occur	Adequacy of follow up of cohorts (attrition)
Pruchno 1990 [50]	USA	✓	✓	✓	✓	×[1]	×	✓	×
Vitaliano 1993 [44]	USA	✓	✓	✓	✓	×	×	✓	✓
Wright 1994 [42]	USA	✓	✓	✓	✓	×	✓	✓	✓
Markiewicz 1997 [49]	Canada	✓	✓	✓	✓	×	✓	✓	✓
Bannister 1998 [45]	UK	✓	✓	✓	✓	×	×	✓	✓
Wells 1998 [47]	Australia	✓	✓	✓	✓	×	×	✓	×
Wright 1998 [43]	USA	×	×	✓	✓	×	×	✓	✓
Caron 1999 [36]	USA	×	×	✓	✓	×	×	✓	✓
Fisher 1999 [39]	USA	✓	✓	✓	✓	×	×	✓	✓
Torrossian 1999 [48]	USA	×	✓	✓	✓	×	×	✓	×
Spruytte 2001 [38]	Belgium	✓	✓	✓	✓	×	×	×	✓
Burgener 2002 [20]	USA	✓	✓	✓	✓	×	×	✓	✓
de Vugt 2004 [46]	Netherlands	✓	✓	✓	×	×	×	✓	✓
McClendon 2004 [51]	USA	×	✓	✓	✓	×	✓	✓	×
Stevens 2004 [40]	USA	✓	✓	✓	✓	×	×	✓	✓
Perren 2007 [41]	Switzerland	✓	✓	✓	✓	×	×	✓	✓
Kunik 2010 (4 papers) [32-35]	USA	✓	✓	✓	✓	×[1]	×	✓	✓
Clare 2014 [37]	UK	×	✓	✓	✓	×	×	✓	×
Shroff 2015[52]	USA	✓	✓	✓	✓	×	×	×	×
Snyder 2016[53]	USA	✓	✓	✓	✓	×[1]	✓	✓	×

✓= study dealt with this adequately. ×= study was at risk of bias in this area, or provided no information to demonstrate otherwise.

[1.] These studies adjusted for three out of the four key confounders

For peer review only - http://bmjopen.bmj.com/site/about/guidelines.xhtml

Primary outcome: risk factors for institutionalisation (Table 3)

Ten studies examined 25 different relationship quality factors as potential risk factors for institutionalisation. Follow-up ranged from 6 to 24 months. The majority of studies found no association between the risk factors investigated and the incidence of institutionalisation. Although some individual studies reported associations between relationship quality and institutionalisation, there were no consistent findings across risk factors, and the lack of appropriate adjustment for basic confounding factors makes interpreting the results very difficult.

For peer review only

For peer review only - http://bmjopen.bmj.com/site/about/guidelines.xhtml

Table 3. Associations between relationship factors and institutionalisation (10 studies)

Risk factor	Study	N	Follow-up	Results (95% CI; p-value)	Analysis adjusted for[1]
**Factors relating to the interaction between the person with dementia and their caregiver**					
Quality of relationship	Spruytte[38]	144	6-9m	OR 0.92 (p=0.02)	NR
	Pruchno[50]	220	12m	OR 1.31 (p>0.05)	a,g,s,r,t,c,bp,ADL, ci,m,hs,cb
Marital happiness At baseline	Wright[42]	29	24m	PoV 0.313 (p<0.01)	NR
Before dementia onset				PoV 0.045 (p>0.05)	NR
Emotional closeness	Fisher[39]	164	24m	OR 1.64 (1.09, 2.46; p=0.02)	d,ba,nf, oc,fe
Emotional distancing /boundary ambiguity	Fisher[39]	164	24m	OR 1.25 (0.85, 1.83; p=0.26)	d,ba,nf,oc,fe
	Wells[47]	93	12-18m	OR 1.3 (p>0.05)	a,d,t
Cohesion	Torossian[48]	197	24m	MD NR (p>0.05)	NR
	Wright[42]	29	24m	PoV 0.472 (p<0.001)	NR
Affection	Wright	29	24m	PoV 0.144 (p>0.05)	NR
Warmth*	Spruytte[38]	144	6-9m	NR (p<0.05)	NR
Anxious-ambivalent attachment (carer)	Markiewicz[49]	108	24m	OR 1.27 (p=0.36)	NR
Avoidant attachment (carer)	Markiewicz[49]	108	24m	OR 2.39 (p<0.001)	NR
	Stevens[40]	215	24m	HR 1.011 (p=0.37)	NR
High levels of tension	Wright[42]	29	24m	PoV 0.162 (p>0.05)	NR
Excessive criticism*	Spruytte[38]	144	6-9m	NR (p>0.05)	NR
**Factors mainly relating to the caregiver**					
Limited adaptability	Torossian[48]	197	24m	MD NR (p>0.05)	NR
Approach coping	Stevens[40]	215	24m	HR 0.997 (p=0.77)	NR
Directing relative's behaviour	Bannister[45]	116	12m	MD 0.2 (p=0.40)	NR
Keeping relative busy	Bannister[45]	116	12m	MD 0.5 (p=0.02)	NR
Learning about the illness	Bannister[45]	116	12m	MD 0.3 (p=0.42)	NR
Prioritizing	Bannister[45]	116	12m	MD 0.1 (p=0.52)	NR
Reducing expectations	Bannister[45]	116	12m	MD 0.4 (p=0.19)	NR
Consistent larger sense of the illness	Bannister[45]	116	12m	MD 0.4 (p=0.35)	NR
Positivity	Bannister[45]	116	12m	MD 0.5 (p=0.57)	NR
	Wells[47]	93	12-18m	OR 1.03 (p>0.05)	a,d
Seeking social support	Wells[47]	93	12-18m	OR 1.91 (p<0.05)	a,d
	Snyder [53]	233	NR	HR 1.159 (0.718, 1.87; p=0.55)	a,d,g,nc
Accepting responsibility	Wells[47]	93	12-18m	OR 0.28 (p<0.01)	a,d
Confrontational	Wells[47]	93	12-18m	OR 2.12 (p<0.05)	a,d
Negative feelings	Fisher[39]	164	24m	OR 1.47 (0.99, 2.19; p=0.05)	d,ba,oc,ec,fe

Legend: **OR** = odds ratio. OR>1 indicates increased risk, OR<1 indicates decreased risk. **HR** = hazard ratio, interpreted the same as OR. **PoV** = proportion of variance. This is a number between 0-1 indicating the proportion of variance in the outcome explained by the exposure. Higher numbers indicate greater explanatory power of the exposure. **Corr** = correlation. This is a number between -1 to +1 and indicates the degree to which the exposure and outcome vary together. Positive numbers indicate that exposure and outcome increase together, negative numbers indicate that the outcome increases as the exposure decreases. Larger numbers indicate stronger correlation. **MD** = mean difference in outcome. Large differences between groups suggests that the exposure might affect the outcome. **NR** = information not reported (where possible results and 95%CIs were calculated from raw data)

1. Pre-specified key confounders: **a** = age; **g** = gender; **s** = socioeconomic status; **d** = dementia severity.

All other confounders: ADL= Activities of Daily Living; ba=Boundary ambiguity; bp= Behaviour Problems; c=number of children; ci= caregiver illness; hs=help services used; ,cb= caregiver burden; ec= Emotional Closeness; fe= Family efficiency;

For peer review only - http://bmjopen.bmj.com/site/about/guidelines.xhtml

m= medication; nc= non-coresidency; nf=negative feeling; oc= Organised cohesiveness; r= religion; t= time spent (duration of) caregiving;
*These are the subscales of the relationship quality scale

Risk factors for challenging behaviour (BPSD) (Table 4)

Under the umbrella term of challenging behaviour four main types of outcome were evaluated: global challenging behaviour scores; psychotic symptoms; depression; and other BPSD outcomes. Eight studies examined nine different relationship quality factors as potential risk factors for these aspects of challenging behaviour. The length of follow-up ranged from 6 to 24 months.

There was a suggestion of an association between relationship factors and global challenging behaviour. All studies evaluating global challenging behaviour provided statistical evidence of an association (most p-values below 0.02). However, one study that reported two analyses did not report effect sizes.[46] For another the reported effect size was very small (mean difference of 0.23 in a scale of 1-144).[41] A larger effect size was seen for the association between expressed emotion and global challenging behaviours (mean difference of 1.9 in a scale of 0-8).[44] None of these analyses adjusted for our pre-specified confounding factors.

Most studies found no evidence of an association between relationship factors and either psychotic symptoms, depression or other BPSD outcomes. However, some of these were small studies that may have been underpowered to detect an association. One study adjusted for three out of four pre-specified potential confounders.[33] This was also one of the largest studies (n=171). It found no evidence for an association between couple mutuality and psychotic symptoms and a very weak, likely clinically negligible effect of this factor on depression (mean difference -0.43 points on a scale of 0-68).[33]

For peer review only - http://bmjopen.bmj.com/site/about/guidelines.xhtml

Table 4. Associations between relationship factors and Challenging behaviour (BPSD) (8 studies)

Risk factor	Study	N	Follow-up	Results (95% CI; p-value)	Analyses adjusted for[1]	Outcome assessment tool, scale range[2]
**Outcome: Global challenging behaviour (BPSD), or problem behaviour (4 studies)**						
Emotional distancing /boundary ambiguity	Caron[36]	60	12m	Corr 0.27 (p<0.001) (overall)	NR	BEHAVE-AD, 0-75
Non-adaptive coping	deVugt[46]	69	6m	MD NR (p=0.007)	g,s,ct	NPI, 1-144
		69	12m	MD NR (p=0.019)	g,s,ct	
Avoidant attachment	Perren[41]	68	24m	MD 0.23 (p<0.05)	NR	NPI, 1-144
Expressed emotion	Vitaliano[44]	77	15-18m	MD 1.9 (0.77, 3.04; p<0.001)	NR	SCB, 0-8
**Outcome: Psychotic symptoms (3 studies)**						
Mutuality	Ball[33]	171	24m	MD -0.1 (-0.24, 0.04; p>0.05) (delusions)	a,g,d,e	NPI, 1-12
	Ball[33]	171	24m	MD -0.04 (-0.16, 0.08; p>0.05) (hallucinations)	a,g,d,e	
Emotional distancing /boundary ambiguity	Caron[36]	60	12m	Corr 0.30 (p<0.001) (paranoia)	NR	BEHAVE-AD, 0-75
Non-adaptive coping	deVugt[46]	69	12m	MD NR (p>0.05) (psychosis)	g,s	NPI, 1-24
**Outcome: Depression (5 studies)**						
Mutuality	Ball[33]	171	24m	MD -0.43 (-0.80, 0.06; p<0.05)	a,g,d,e	Hamilton, 0-68
Quality of relationship	Burgener[20]	70	18m	Corr 0.22 (p>0.05)	NR	Cornell, 0-38
Emotional distancing /boundary ambiguity	Caron[36]	60	12m	Corr 0.01 (p>0.05)	NR	BEHAVE-AD, 0-75
Expressed emotion	Vitaliano[44]	77	15-18m	MD 2.00 (-0.31, 4.31; p>0.05)	NR	Hamilton, 0-68
Cohesion quantity	Wright[43]	14	6m	Corr -0.302 (p>0.05)	NR	Zung, 0-270
quality		14	6m	Corr -0.387 (p>0.05)	NR	
Affection quantity	Wright[43]	14	6m	Corr -2.41 (p>0.05)	NR	
quality		14	6m	Corr -0.038 (p>0.05)	NR	
Tension quantity	Wright[43]	14	6m	Corr 0.533 (p<0.01)	NR	
quality		14	6m	Corr -0.288 (p>0.05)	NR	
**Outcome: Other**						
Non-adaptive coping	deVugt[46]	69	12m	MD NR (p=0.512) (apathy)	g,s,ct	NPI, 1-48
Non-adaptive coping	deVugt[46]	69	12m	MD NR (p=0.005) (hyperactivity)	g,s,ct	NPI, 1-60
Mutuality	Morgan[34] & Kunik[32]	171	24m	MD -0.42 (-0.64, 0.20; p<0.001) HR 0.63 (0.45, 2.87; p=0.006) (aggression)	NR	CMAI, 0-156
Emotional distancing /boundary ambiguity	Caron[36]	60	6m	Corr 0.15 (p>0.05) (anxiety)	NR	BEHAVE-AD, 0-75

Legend: **OR** = odds ratio. OR>1 indicates increased risk, OR<1 indicates decreased risk. **HR** = hazard ratio, interpreted the same as OR. **PoV** = proportion of variance. This is a number between 0-1 indicating the proportion of variance in the outcome explained by the exposure. Higher numbers indicate greater explanatory power of the exposure. **Corr** = correlation. This is a number between -1 to +1 and indicates the degree to which the exposure and outcome vary together. Positive numbers indicate that exposure and outcome increase together, negative numbers indicate that the outcome increases as the exposure decreases. Larger numbers indicate stronger correlation. **MD** = mean difference in outcome. Large differences between groups suggests that the exposure might affect the outcome. **NR** = information not reported (where possible results and 95%CIs were calculated from raw data)

1. Pre-specified key confounders: **a** = age; **g** = gender; **s** = socioeconomic status; **d** = dementia severity.
All other confounders: ct=carer type; e= ethnicity.

2. NPI = Neuropsychiatric Inventory; CMAI = Cohen-Mansfield Agitation Inventory; Cornell = Cornell Scale for Depression in Dementia; SCB = Screen for Caregiver Burden (selection of items); Zung = Short Zung Interviewer Assisted Depression Rating Scale;

For peer review only - http://bmjopen.bmj.com/site/about/guidelines.xhtml

For peer review only

Risk factors for hospitalisation, quality of life, and death (Table 5)

The outcomes of hospitalisation,[35] were examined in one study and quality of life[37,52] and death[51, 53] were each examined in two studies. . The small number of poor quality studies means it is not possible to draw conclusions regarding the association of relationship factors with these outcomes.

Table 5. Associations between relationship factors and hospitalisation, quality of life, and time to death

Risk factor	Study	N	Follow-up	Results (95% CI; p-value)	Analyses adjusted for[1]
**Hospitalisation (1 study)**					
Relationship strain	Godwin[35]	296	12m	OR 1.03 (0.92, 1.14; 0.637)	NR
**Quality of life (2 studies)**					
Quality of relationship (patient view)	Clare[37]	51	20m	MD 0.31 (p=0.06)	pqol,pd, cs, cqor
	Shroff[52]	83	NR	R^2 0.179 (p<0.001)	NR
Quality of relationship (carer view)	Clare[37]	51	20m	MD -0.13 (p=0.89)	pqol,pd, cs, cqor
**Time to death (2 studies)**					
Instrumental coping	McClendon[51]	141	5-9 years	HR 0.99 (p=0.915)	NR
Acceptance coping	McClendon[51]	141	5-9 years	HR 1.09 (p=0.644)	NR
Wishful thinking	McClendon[51]	141	5-9 years	HR 1.41 (p=0.019)	NR
	Snyder[53]	233	NR	HR 0.88 (0.673, 1.171; 0.4)	a,d,g,nc
Problem Focused coping	Snyder[53]	233	NR	HR 0.80 (0.571, 1.128; 0.2)	a,d,g,nc
Seeking Social Support	Snyder[53]	233	NR	HR 1.056 (0.787-1.416; 0.7)	a,d,g,nc
Blaming Self	Snyder[53]	233	NR	HR 0.967 (0.768-1.218; 0.7)	a,d,g,nc
Avoidance coping	Snyder[53]	233	NR	HR1.021 (0.720-1.448; 0.9)	a,d,g,nc
Blaming Others	Snyder[53]	233	NR	HR 0.867 (0.632-1.190; 0.3)	a,d,g,nc
Counting Blessings	Snyder[53]	233	NR	HR 0.648 (0.454-0.926; 0.017)	a,d,g,nc
Religiosity	Snyder[53]	233	NR	HR 0.882 (0.682-1.142; 0.341)	a,d,g,nc

Legend: OR = odds ratio. OR>1 indicates increased risk, OR<1 indicates decreased risk. HR = hazard ratio, interpreted the same as OR. MD = mean difference in outcome. Large differences between groups suggests that the exposure might affect the outcome. NR = information not reported (where possible results and 95%CIs were calculated from raw data)
1. Pre-specified key confounders:
a = age; **g** = gender; **s** = socioeconomic status; **d** = dementia severity.
All other confounders: Pqol=PWD quality of life; pd= PWD depression; cqor=carer quality of relationship; cs=carer stress; nc= non-coresidency

For peer review only - http://bmjopen.bmj.com/site/about/guidelines.xhtml

DISCUSSION

This systematic review assessed the evidence on the role of relationship factors on outcomes in dementia. Although it is plausible that relationship factors could increase the risk of institutionalisation, challenging behaviour and other outcomes, there is currently no robust evidence to establish which, or to what extent, elements of the caring relationship affect specific outcomes.

The majority of studies found no association between specific risk factors and the outcomes of interest. However, this is not necessarily evidence of a lack of association, as some of the studies will have been underpowered to detect differences, and most were at risk of confounding as they failed to adjust for even the most basic confounding factors. There was a suggestion of an association between factors related to the emotional withdrawal of the caregiver and subsequent increased risk of challenging behaviour in the person with dementia. All studies in this category found statistical evidence of an association. However, the methodological quality of these studies was poor. For example many did not report effect sizes while in others the effect sizes were small, suggesting that associations may not be clinically important. This could also be because the sample was too small to detect a difference. No study reported justification for their sampling or the sample sizes. There was also a potential for confounding in studies that did report effect sizes.

Amongst the strengths of this review were that we conducted a thorough, sensitive search which will have minimised the chance of missing relevant studies. By limiting inclusion to cohort studies only, we avoided interpretive difficulties from recall bias (systematic differences in how people remember risk factors) and reverse causation (when a purported risk factor is in fact a result of the outcome). The double-screening of each record by two reviewers working independently also minimised the possibility of errors and selection bias in the identification of eligible reports. All data extracted was checked in full by a second reviewer working independently.

For peer review only - http://bmjopen.bmj.com/site/about/guidelines.xhtml

A limitation of our review is that we were not able to assess for publication bias or selective reporting of results. It is known that studies with null findings are less likely to be published, and authors can selectively report their 'more interesting' findings.[54] This means it is possible there are other relevant results that have not been reported, and so would not be available to a review. We attempted to minimise the risk of publication bias by including a grey literature search.

Our ability to answer the research question was limited due to unclear and incomplete reporting, few studies assessing similar risk factors, and the potential for bias in included studies. We considered the appropriateness of using meta-analysis to produce summary estimates. However, many studies did not report full details of effect sizes and confidence intervals that are required for meta-analysis. Additionally most risk factor-outcome relationships were only reported in one or two studies (with conflicting results in the latter cases) which is insufficient for meta-analyses. It would not have been meaningful or appropriate to combine different risk factors or outcomes in pooled analysis. We assessed the potential for bias in included studies and took these assessments into consideration when reaching conclusions, thus avoiding over-interpretation of findings from studies with potentially serious problems.

Very few studies adjusted their analyses for key pre-specified confounding factors (age, gender, socioeconomic status, and dementia severity). Two thirds of included analyses did not adjust for any confounding factors. This is an important limitation as it means any findings could be explained by differences in these factors across the exposure groups – differences that are very likely in a non-randomised study where participants self-select into exposure groups. A small number of studies adjusted for other factors, but these were factors such as quality of life and emotional withdrawal that may well be on the causal pathway between the relationship factor studied and the outcome (so not a true confounder). Reporting of results was incomplete with many studies only reporting p-values, or just that an association was 'statistically significant' / 'not significant' with no effect sizes

For peer review only

For peer review only - http://bmjopen.bmj.com/site/about/guidelines.xhtml

or confidence intervals. This is another important limitation as in these cases we have no information about the probable magnitude of effect, and it is impossible to interpret the potential clinical implications of any statistical difference. Inadequate reporting of methods and results made it difficult to assess risk of bias. Very few studies evaluated the same risk factors, so there is also very little evidence on any individual factor. Duration of follow-up (typically 6-24 months), although theoretically long enough for an outcome to have occurred, may not have been sufficient to detect outcomes in the majority of the sample in a study. As dementia is typically a slowly progressing disease this may not be sufficient for long-term outcomes such as institutionalisation.

Finally, majority of the studies identified were published before 2000. The most recent study is from 2016, with only three studies reported in the last five years. The reasons for this apparent diminishing of literature are unclear, but the relative absence of more recent studies may have implications for applicability of the findings of this review. New long-term studies that are well conducted and fully reported are needed to reliably answer questions regarding effect of family relationship quality on institutionalisation risks.

The current evidence does not provide a basis by which GPs or other health professionals could reliably identify people at risk of poor outcome on the basis of relationship factors. However, the lack of robust evidence about the role of relationship factors does not imply that personal relationships are not important factors in dementia outcomes; many professionals working with families consider these to be important.[55 56] One plausible mechanism is that factors such as emotional withdrawal of the caregiver might prompt 'challenging behaviour' in the person with dementia as an attempt to elicit an emotional connection.[41] For instance, care-giver coping strategies such as constantly 'correcting' the person with dementia, rather than accepting their cognitive challenges, could be construed as potentially undermining the individual's personhood or self-esteem, both of which are seen as key elements in good dementia care. In this way, any 'challenging behaviour' might be construed as behavioural expressions of underlying frustration or distress. It is also possible that challenging behaviours are themselves on the causal pathway

For peer review only - http://bmjopen.bmj.com/site/about/guidelines.xhtml

between relationship factors and institutionalisation – the latter plausibly becoming more likely as the caregiver becomes less able to cope with challenging behaviours. If this is the case then longer follow-up may be necessary to detect associations between relationship factors and institutionalisation. In addition, the association between quality of relationships, challenging behaviour and subsequent institutionalisation is likely to be complex. A recent study[54] found that relationship quality was one of a number of psychosocial factors associated with caregiver distress at challenging behaviour independently of the frequency of that behaviour. Similarly changes in the meaning of their relationship, and in particular the belief that their relative had lost, or would inevitably lose, their identity to dementia, is a fundamental reason why family carers experienced behaviour as challenging[55]. It may be, then, that the quality of relationship acts as a confounding variable in the association between challenging behaviour and institutionalisation.

Lack of evidence on what relationship factors predict outcomes in people with dementia should not preclude further evaluation of psychosocial interventions targeting people with dementia and their carers. Such studies could use experimental designs to identify which interventions work and in which settings. It may also be useful to look to qualitative research, exploring the views of people with dementia and their family carers on what they consider important for continued living at home, and the challenges in the relationship that they face.

CONCLUSIONS

There is currently no strong or consistent evidence on the effects of relationship factors on institutionalisation, hospitalisation, death or quality of life for people with dementia. There was a suggestion of an association between relationship factors and challenging behaviour, although the evidence for this was weak. As the current focus of dementia care is ‘person-centred’, which prioritises interpersonal relationships, this lack of evidence about the role of relationships in

For peer review only - http://bmjopen.bmj.com/site/about/guidelines.xhtml

dementia outcomes is striking. To improve our ability to support those with dementia and their families this evidence gap needs to be addressed.

FIGURES

Figure 1. PRISMA flow diagram

For peer review only

For peer review only - http://bmjopen.bmj.com/site/about/guidelines.xhtml

Funding statements and acknowledgements

This research is supported by the National Institute for Health Research (NIHR) Collaboration for Leadership in Applied Health Research and Care West at University Hospitals Bristol NHS Foundation Trust. The views expressed are those of the author(s) and not necessarily those of the NHS, the NIHR or the Department of Health. Authors thank Rebecca Beynon for support screening papers.

Competing interests

The authors declare that they have no competing interests.

Authors' contributions

HE was the lead researcher, contributed to the study design, carried out screening, data extraction, data analysis, and prepared the manuscript. SI carried out screening, data extraction, the risk of bias assessment, and revisions of the manuscript after the update search in Summer 2017. VL and JS contributed to the screening and data extraction. JS and PW led the study design, supervised the review and contributed to drafting the manuscript. AR developed the search strategy and conducted the searches. RC and SC conceived the original study idea, and contributed to the study design. RC provided clinical advice throughout the review and made critical revisions to the manuscript. All authors read and approved the final manuscript.

Data sharing statement

There is no additional unpublished data from this study. Any requests for access to the original data extracted from included papers should be sent to the corresponding author.

For peer review only

References

1. Fineberg NA, Haddad PM, Carpenter L, et al. The size, burden and cost of disorders of the brain in the UK. *J Psychopharm* 2013 doi: 10.1177/0269881113495118

2. Luengo-Fernandez R, Leal J, Gray AM. UK research expenditure on dementia, heart disease, stroke and cancer: are levels of spending related to disease burden? *Eur J Neurol* 2012;19(1):149-54. doi: 10.1111/j.1468-1331.2011.03500.x

3. World Health Organization. Dementia (Fact sheet no. 362). 2015. Available from: http://wwwwhoint/mediacentre/factsheets/fs362/en/ accessed 04/09/2017.

4. Prince M, Bryce R, Albanese E, et al. The global prevalence of dementia: A systematic review and metaanalysis. *Alzheimers Dement* 2013;9(1):63-75.e2. doi: 10.1016/j.jalz.2012.11.007

5. Prince M, Knapp M, Guerchet M, et al. Dementia UK Update (Second Edition): Alzheimer's Society, 2014.

6. Alzheimer's Disease International. World Alzheimer Report 2014: Dementia and Risk Reduction. An analysis of protective and modifiable factors, 2014.

7. Byrne-Davis LMT, Bennett PD, Wilcock GK. How are Quality of Life Ratings Made? Toward a Model of Quality of Life in People with Dementia. *Qual Life Res* 2006;15(5):855-65. doi: 10.1007/s11136-005-5416-9

8. Donnelly L. Care Quality Commission finds 'appalling' failings in elderly care. The Telegraph, October 2014.

9. Sawer P, Ross, T. Dozens of care homes failing the most vulnerable. The Telegraph, December 2014.

10. Nelson C. Care home concerns: YouGov UK; 6 March 2013. Available from: https://yougov.co.uk/news/2013/03/06/care-home-concerns/ accessed 04/09/2017.

11. Department of Health. Prime Minister's challenge on dementia 2020. Department of Health; February 2015. Available from:

For peer review only - http://bmjopen.bmj.com/site/about/guidelines.xhtml

https://www.gov.uk/government/uploads/system/uploads/attachment_data/file/414344/pm-dementia2020.pdf accessed 04/09/2017.

12. Tschanz JT, Piercy K, Corcoran CD, et al. Caregiver Coping Strategies Predict Cognitive and Functional Decline in Dementia: The Cache County Dementia Progression Study. *Am J Geriatr Psychiatry* 2013;21(1):57-66. doi: 10.1016/j.jagp.2012.10.005

13. García-Alberca JM, Cruz B, Lara JP, et al. The experience of caregiving: the influence of coping strategies on behavioral and psychological symptoms in patients with Alzheimer's disease. *Aging Ment Health* 2013;17(5):615-22. doi: 10.1080/13607863.2013.765833

14. Luppa M, Riedel-Heller SG, Stein J, et al. Predictors of Institutionalisation in Incident Dementia – Results of the German Study on Ageing, Cognition and Dementia in Primary Care Patients (AgeCoDe Study). *Dement Geriatr Cogn Disord* 2012;33(4):282-88.

15. Fauth E, Hess K, Piercy K, et al. Caregivers' relationship closeness with the person with dementia predicts both positive and negative outcomes for caregivers' physical health and psychological well-being. *Aging Ment Health* 2012;16(6):699-711. doi: 10.1080/13607863.2012.678482

16. Quinn C, Clare L, Woods B. The impact of the quality of relationship on the experiences and wellbeing of caregivers of people with dementia: A systematic review. *Aging Ment Health* 2009;13(2):143-54. doi: 10.1080/13607860802459799

17. Norton MC, Piercy KW, Rabins PV, et al. Caregiver–Recipient Closeness and Symptom Progression in Alzheimer Disease. The Cache County Dementia Progression Study. *J Gerontol B Psychol Sci Soc Sci* 2009;64B(5):560-68. doi: 10.1093/geronb/gbp052

18. Spitznagel MB, Tremont G, Davis JD, et al. Psychosocial Predictors of Dementia Caregiver Desire to Institutionalize: Caregiver, Care Recipient, and Family Relationship Factors. *J Geriatr Psychiatry Neurol* 2006;19(1):16-20. doi: 10.1177/0891988705284713

19. Banerjee S, Murray J, Foley B, et al. Predictors of institutionalisation in people with dementia. *J Neurol Neurosurg Psychiatry* 2003;74(9):1315-16. doi: 10.1136/jnnp.74.9.1315

For peer review only

For peer review only - http://bmjopen.bmj.com/site/about/guidelines.xhtml

20. Burgener S, Twigg P. Relationships among caregiver factors and quality of life in care recipients with irreversible dementia. *Alzheimer Dis Assoc Disord* 2002;16(2):88-102.

21. Bergmann K, Manchee V, Woods RT. Effect of family relationships on psychogeriatric patients. *J R Soc Med* 1984;77(10):840-44.

22. Benbow SM, Sharman V. Review of family therapy and dementia: twenty-five years on. *Int Psychogeriatr* 2014;26(Special Issue 12):2037-50.

23. Cheston R, Ivanecka A. Individual and group psychotherapy with people diagnosed with dementia: a systematic review of the literature. *Int J Geriatr Psychiatry* 2016:n/a-n/a. doi: 10.1002/gps.4529

24. Edwards HB, Savović J, Whiting P, et al. Quality of relationships as predictors of outcomes in people with dementia: a systematic review protocol. *BMJ Open* 2016;6(4) doi: 10.1136/bmjopen-2015-010835

25. Reynolds J, Houlson C, Coleman L. Understanding relationship quality London: One plus One; 2014. Available from: http://www.oneplusone.org.uk/wp-content/uploads/2014/02/UnderstandingRelationship-Quality-by-Jenny-Reynolds-Dr-Catherine-Houlston-and-Dr-Lester-Coleman.pdf accessed 04/09/2017.

26. Hazan C, Shaver P. Romantic love conceptualized as an attachment process. *J Pers Soc Psychol* 1987;52(3):511-24. doi: 10.1037/0022-3514.52.3.511

27. Hazan C, Shaver PR. Attachment as an Organizational Framework for Research on Close Relationships. *Psychol Inq* 1994;5(1):1-22.

28. Kuipers L, Bebbington P. Expressed emotion research in schizophrenia: theoretical and clinical implications. *Psychol Med* 1988;18(4):893-909. doi: 10.1017/S0033291700009831

29. Raune D, Kuipers E, Bebbington PE. Expressed emotion at first-episode psychosis: investigating a carer appraisal model. *Br J Psychiatry* 2004;184(4):321-26. doi: 10.1192/bjp.184.4.321

For peer review only - http://bmjopen.bmj.com/site/about/guidelines.xhtml

30. Wells GA, Shea B, O'Connell D, et al. The Newcastle-Ottawa Scale (NOS) for assessing the quality of nonrandomised studies in meta-analyses. 2014. Available from: http://www.ohri.ca/programs/clinical_epidemiology/oxford.asp accessed 04/09/2017.

31. Jüni P, Witschi A, Bloch R, et al. The hazards of scoring the quality of clinical trials for meta-analysis. *JAMA* 1999;282(11):1054-60. doi: 10.1001/jama.282.11.1054

32. Kunik ME, Snow AL, Davila JA, et al. Causes of aggressive behavior in patients with dementia. *J Clin Psychiatry* 2010;71(9):1145-52. doi: 10.4088/JCP.08m04703oli

33. Ball V, Snow AL, Steele AB, et al. Quality of Relationships as a Predictor of Psychosocial Functioning in Patients With Dementia. *J Geriatr Psychiatry Neurol* 2010;23(2):109-14. doi: 10.1177/0891988710363709

34. Morgan RO, Sail KR, Snow AL, et al. Modeling Causes of Aggressive Behavior in Patients With Dementia. *Gerontologist* 2013;53(5):738-47. doi: 10.1093/geront/gns129

35. Godwin KM, Morgan RO, Walder A, et al. Predictors of Inpatient Utilization among Veterans with Dementia. *Curr Gerontol Geriatr Res* 2014;2014:10. doi: 10.1155/2014/861613

36. Caron W, Boss P, Mortimer J. Family boundary ambiguity predicts Alzheimer's outcomes. *Psychiatry* 1999;62(4):347-56.

37. Clare L, Woods RT, Nelis SM, et al. Trajectories of quality of life in early-stage dementia: individual variations and predictors of change. *Int J Geriatr Psychiatry* 2014;29(6):616-23. doi: 10.1002/gps.4044

38. Spruytte N, Van Audenhove C, Lammertyn F. Predictors of institutionalization of cognitively-impaired elderly cared for by their relatives. *Int J Geriatr Psychiatry* 2001;16(12):1119-28. doi: 10.1002/gps.484

39. Fisher L, Lieberman MA. A Longitudinal Study of Predictors of Nursing Home Placement for Patients With Dementia: The Contribution of Family Characteristics. *Gerontologist* 1999;39(6):677-86. doi: 10.1093/geront/39.6.677

For peer review only - http://bmjopen.bmj.com/site/about/guidelines.xhtml

40. Stevens A, Owen J, Roth D, et al. Predictors of time to nursing home placement in White and African American individuals with dementia. *J Aging Health* 2004;16(3):375-97.

41. Perren S, Schmid R, Herrmann S, et al. The impact of attachment on dementia-related problem behavior and spousal caregivers' well-being. *Attach Hum Dev* 2007;9(2):163-78. doi: 10.1080/14616730701349630

42. Wright LK. Alzheimer's disease afflicted spouses who remain at home: can human dialectics explain the findings? *Soc Sci Med* 1994;38(8):1037-46.

43. Wright LK, Hickey JV, Buckwalter KC, et al. Spousal interactions in Alzheimer's disease and stroke caregiving: Relationship to care recipients' functional abilities and physical and emotional health. *J Am Psychiatr Nurses Assoc* 1998;4(6):169-81. doi: 10.1016/S1078-3903%2898%2990028-3

44. Vitaliano PP, Young HM, Russo J, et al. Does Expressed Emotion in Spouses Predict Subsequent Problems Among Care Recipients With Alzheimer's Disease? *J Gerontol* 1993;48(4):P202-P09. doi: 10.1093/geronj/48.4.P202

45. Bannister C, Ballard C, Lana M, et al. Placement of dementia sufferers in residential and nursing home care. *Age Ageing* 1998;27(2):189-93.

46. de Vugt ME, Stevens F, Aalten P, et al. Do caregiver management strategies influence patient behaviour in dementia? *Int J Geriatr Psychiatry* 2004;19(1):85-92. doi: 10.1002/gps.1044

47. Wells Y, Over R. Institutional Placement of a Dementing Spouse: The Influence of Appraisal, Coping Strategies, and Social Support. *Aust J Psychol* 1998;50(2):100-05. doi: 10.1080/00049539808257540

48. Torossian CL, Ruffins S. Relationship Between Family Dynamics of Caregivers, Depression, and the Likelihood of Institutionalization of Alzheimer's Patients. *J Contemp Psychother* 1999;29(2):127-42. doi: 10.1023/a:1021956715387

49. Markiewicz D, Reis M, Gold DP. An exploration of attachment styles and personality traits in caregiving for dementia patients. *Int J Aging Hum Dev* 1997;45(2):111-32.

For peer review only - http://bmjopen.bmj.com/site/about/guidelines.xhtml

50. Pruchno RA, Michaels JE, Potashnik SL. Predictors of Institutionalization Among Alzheimer Disease Victims With Caregiving Spouses. *J Gerontol* 1990;45(6):S259-S66. doi: 10.1093/geronj/45.6.S259

51. McClendon MJ, Smyth KA, Neundorfer MM. Survival of Persons With Alzheimer's Disease: Caregiver Coping Matters. *Gerontologist* 2004;44(4):508-19. doi: 10.1093/geront/44.4.508

52. Shroff HB. Family resiliency, sense of coherence, social support and psychosocial interventions: Reducing caregiver burden and determining the quality of life in persons with alzheimer's disease [Doctor of Philosophy]. Florida Atlantic University, 2014.

53. Snyder CM. Dementia caregiver personality traits and coping strategies: Association with care recipient outcomes [Doctor of Philosophy]. Utah State University, 2015.

54. Dwan K, Gamble C, Williamson PR, et al. Systematic Review of the Empirical Evidence of Study Publication Bias and Outcome Reporting Bias — An Updated Review. *PLoS One* 2013;8(7):e66844. doi: 10.1371/journal.pone.0066844

55. Kitwood T. Dementia Reconsidered: The Person Comes First Buckingham: Open University Press 1997.

56. Cheston R, Bender M. Understanding Dementia: The Man with the Worried Eyes. London: Jessica Kingsley Press 1999.

For peer review only

For peer review only - http://bmjopen.bmj.com/site/about/guidelines.xhtml

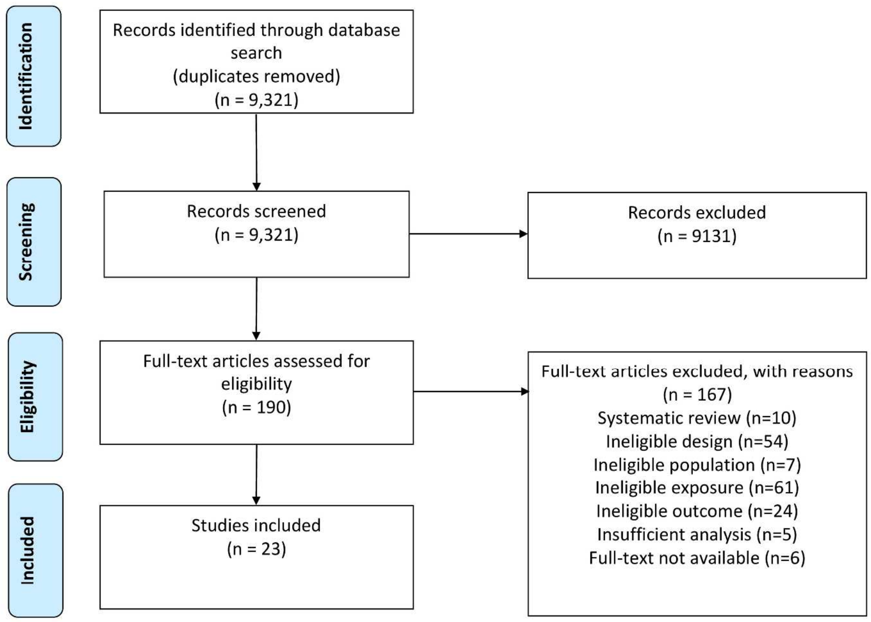

Figure 1. PRISMA flow diagram

254x190mm (300 x 300 DPI)

PRISMA 2009 Checklist

Section/topic	#	Checklist item	Reported on page #
**TITLE**			
Title	1	Identify the report as a systematic review, meta-analysis, or both.	1
**ABSTRACT**			
Structured summary	2	Provide a structured summary including, as applicable: background; objectives; data sources; study eligibility criteria, participants, and interventions; study appraisal and synthesis methods; results; limitations; conclusions and implications of key findings; systematic review registration number.	2
**INTRODUCTION**			
Rationale	3	Describe the rationale for the review in the context of what is already known.	3-4
Objectives	4	Provide an explicit statement of questions being addressed with reference to participants, interventions, comparisons, outcomes, and study design (PICOS).	4
**METHODS**			
Protocol and registration	5	Indicate if a review protocol exists, if and where it can be accessed (e.g., Web address), and, if available, provide registration information including registration number.	4
Eligibility criteria	6	Specify study characteristics (e.g., PICOS, length of follow-up) and report characteristics (e.g., years considered, language, publication status) used as criteria for eligibility, giving rationale.	4-6
Information sources	7	Describe all information sources (e.g., databases with dates of coverage, contact with study authors to identify additional studies) in the search and date last searched.	6
Search	8	Present full electronic search strategy for at least one database, including any limits used, such that it could be repeated.	6
Study selection	9	State the process for selecting studies (i.e., screening, eligibility, included in systematic review, and, if applicable, included in the meta-analysis).	6
Data collection process	10	Describe method of data extraction from reports (e.g., piloted forms, independently, in duplicate) and any processes for obtaining and confirming data from investigators.	7
Data items	11	List and define all variables for which data were sought (e.g., PICOS, funding sources) and any assumptions and simplifications made.	6-7
Risk of bias in individual studies	12	Describe methods used for assessing risk of bias of individual studies (including specification of whether this was done at the study or outcome level), and how this information is to be used in any data synthesis.	7-8
Summary measures	13	State the principal summary measures (e.g., risk ratio, difference in means).	6
Synthesis of results	14	Describe the methods of handling data and combining results of studies, if done, including measures of consistency (e.g., I^2) for each meta-analysis.	6

For peer review only

For peer review only - http://bmjopen.bmj.com/site/about/guidelines.xhtml

PRISMA 2009 Checklist

Section/topic	#	Checklist item	Reported on page #
Risk of bias across studies	15	Specify any assessment of risk of bias that may affect the cumulative evidence (e.g., publication bias, selective reporting within studies).	7
Additional analyses	16	Describe methods of additional analyses (e.g., sensitivity or subgroup analyses, meta-regression), if done, indicating which were pre-specified.	7
**RESULTS**			
Study selection	17	Give numbers of studies screened, assessed for eligibility, and included in the review, with reasons for exclusions at each stage, ideally with a flow diagram.	8
Study characteristics	18	For each study, present characteristics for which data were extracted (e.g., study size, PICOS, follow-up period) and provide the citations.	Table 1
Risk of bias within studies	19	Present data on risk of bias of each study and, if available, any outcome level assessment (see item 12).	Table 2
Results of individual studies	20	For all outcomes considered (benefits or harms), present, for each study: (a) simple summary data for each intervention group (b) effect estimates and confidence intervals, ideally with a forest plot.	Tables 3-5
Synthesis of results	21	Present the main results of the review. If meta-analyses are done, include for each, confidence intervals and measures of consistency	Tables 3-5 plus page 11,13,15
Risk of bias across studies	22	Present results of any assessment of risk of bias across studies (see Item 15).	Not done (see p20)
Additional analysis	23	Give results of additional analyses, if done (e.g., sensitivity or subgroup analyses, meta-regression [see Item 16]).	n/a
**DISCUSSION**			
Summary of evidence	24	Summarize the main findings including the strength of evidence for each main outcome; consider their relevance to key groups (e.g., healthcare providers, users, and policy makers).	20
Limitations	25	Discuss limitations at study and outcome level (e.g., risk of bias), and at review-level (e.g., incomplete retrieval of identified research, reporting bias).	21-22
Conclusions	26	Provide a general interpretation of the results in the context of other evidence, and implications for future research.	23
**FUNDING**			
Funding	27	Describe sources of funding for the systematic review and other support (e.g., supply of data); role of funders for the systematic review.	26

For peer review only - http://bmjopen.bmj.com/site/about/guidelines.xhtml

PRISMA 2009 Checklist

From: Moher D, Liberati A, Tetzlaff J, Altman DG, The PRISMA Group (2009). Preferred Reporting Items for Systematic Reviews and Meta-Analyses: The PRISMA Statement. PLoS Med 6(7): e1000097. doi:10.1371/journal.pmed1000097

For more information, visit: **www.prisma-statement.org**.

For peer review only
